# An Integrated, Case-Based Approach to Teaching Medical Students How to Locate the Best Available Evidence for Clinical Care

**DOI:** 10.15766/mep_2374-8265.10531

**Published:** 2017-01-19

**Authors:** Stephanie M. Swanberg, Misa Mi, Keith Engwall

**Affiliations:** 1 Assistant Professor and Information Literacy and eLearning Librarian, Department of Biomedical Sciences, Oakland University William Beaumont School of Medicine; 2 Associate Professor and Information Literacy and eLearning Librarian, Department of Biomedical Sciences, Oakland University William Beaumont School of Medicine; 3 Assistant Professor and Web and Emerging Technologies Librarian, Department of Biomedical Sciences, Oakland University William Beaumont School of Medicine

**Keywords:** Core Entrustable Professional Activities, Evidence-Based Medicine, Active Learning, Instructional Methods, Information Literacy

## Abstract

**Introduction:**

A major step of the evidence-based medicine (EBM) process is to locate the most current evidence in support of clinical care. This requires identifying and searching appropriate evidence-based resources. Medical library faculty at the Oakland University William Beaumont School of Medicine teach these skills as part of a dedicated EBM course at the end of the second year of the medical school curriculum.

**Methods:**

A 3-hour “Locating the Best Available Evidence” session is divided into two major components: an optional 50-minute didactic lecture followed by a mandatory 2-hour interactive lab. Students formulate a PICO (patient, intervention, comparison, outcome) question from a case, develop search strategies, and gather evidence. Formative feedback is provided to the students to help them prepare for a final case presentation.

**Results:**

Session effectiveness is assessed using course evaluations and the case presentation grade. Course evaluations indicate that students find this session structure to be especially helpful in learning the breadth of available EBM resources, preparing for their course case presentations, and acquiring skills for clinical clerkships. Quality of the case presentations also indicates students have acquired the necessary skills to be successful in practicing EBM skills in clerkship rotations and residency.

**Discussion:**

Whether institutions have a dedicated EBM course or integrate EBM skills into the medical school curriculum, this session could easily be adapted and implemented. It could also be tailored for graduate or continuing medical education environments in any specialty.

## Educational Objectives

By the end of this session, learners will be able to:

1. Formulate a clear, answerable clinical question based on a patient case.
2. Identify the hierarchy of evidence.
3. Access, search for, and evaluate the evidence using preappraised information resources.

## Introduction

In 1991, Gordon Guyatt introduced the term *evidence-based medicine* (EBM) to showcase the importance of not only physician expertise but also current research evidence and patient values in clinical decision-making.^1^ A five-step EBM process emerged soon after to guide clinicians in evidence-based care: (1) asking clear, searchable clinical questions; (2) acquiring the evidence; (3) appraising the quality of evidence; (4) applying that evidence to care; and (5) assessing the effectiveness of the intervention.^2^ EBM is now recognized as essential for everyday clinical practice and a core competency for learners at the undergraduate and graduate medical education levels.^3,4^ Teaching of EBM in undergraduate medical training has been well documented in the literature, with several reviews and systematic reviews published in the last 5 years.^5–9^ EBM instruction has taken many forms, including integration into clinical clerkship rotations or courses, dedicated short courses or electives, stand-alone seminars or workshops, journal clubs, online learning, and problem-based learning.^5,7,8^ Instruction in acquiring the evidence typically uses a combination of teaching methods, including didactic lecture, hands-on instruction, small-group work, and online learning.^7–9^ However, the most effective training method and the long-term effectiveness of EBM training at this level are still unknown.^5,8,9^

At the Oakland University William Beaumont School of Medicine (OUWB), medical library faculty developed a 3-hour instructional session as part of a dedicated EBM course. This course is a 2-week curriculum covering all aspects of the EBM process from developing searchable clinical questions using the PICO (patient, intervention, comparison, and outcome) format^10^ to locating current evidence via online information resources to then critically appraising the evidence and applying it to clinical case scenarios. This session, entitled “Locating the Best Available Evidence,” is the second of eight in the course, is taught to medical students at the end of their second year just prior to starting clinical clerkships, and focuses on the first two steps of the EBM process. Though previous MedEdPORTAL publications have described similar methods for training residents and medical students in developing PICO questions and searching the literature,^11–13^ the current session is distinct in that it has students learn and practice searching multiple EBM resources in a team-based environment and uses a student case presentation as a major assessment method. It is best implemented by clear and sequential integration with other EBM instructional content.

## Methods

The “Locating the Best Available Evidence” session is divided into two major components: an optional 50-minute didactic lecture followed by a mandatory 2-hour interactive lab. To encourage independent, self-directed learning, students may choose to prepare for the lab by attending the lecture or by reviewing online instruction available as either a brief instructional video or a text-based online tutorial.^14^

The lecture (Appendices A & B) reviews the first two steps of the EBM process: forming a clear, searchable clinical question using the PICO question format and searching preappraised, evidence-based resources presented through the 6S pyramid of evidence devised by DiCenso, Bayley, and Haynes.^15^ As many evidence-based resources require a paid subscription for access, please check the availability of resources at your institution through your library or department. A breakdown of subscription and free EBM resources at each tier of the 6S pyramid of evidence is provided in the Table.

**Table.** Overview of Evidence-Based Resources

Tier of Evidence	Subscription Resources	Free Resources
All tiers		ACCESSSS: https://plus.mcmaster.ca/ACCESSSS Trip Database: https://www.tripdatabase.com
Summaries	UpToDate: www.uptodate.com DynaMed/DynaMed Plus: www.dynamed.com	National Guideline Clearinghouse: https://guideline.gov
Synopses of syntheses	ACP Journal Club: http://annals.org/journalclub.aspx	Database of Abstracts of Reviews of Effects (DARE) via PubMed Health: http://www.ncbi.nlm.nih.gov/pubmedhealth/s/dare_reviews_medrev/a/
Syntheses	Cochrane Database of Systematic Reviews: http://www.cochranelibrary.com/cochrane-database-of-systematic-reviews/	PubMed: www.ncbi.nlm.nih.gov/pubmed/ PubMed Health: http://www.ncbi.nlm.nih.gov/pubmedhealth/
Synopses of studies	ACP Journal Club: http://annals.org/journalclub.aspx	
Studies	Embase: https://www.embase.com Scopus: https://www.scopus.com https://www.webofknowledge.com	PubMed: www.ncbi.nlm.nih.gov/pubmed/

The lab portion provides students with an opportunity to practice the first two steps of the EBM process. It is best taught in a flat classroom with movable furniture where students can easily work in teams. Large class sizes should be split into smaller groups of approximately 25 students per instructor, subdivided into teams of four to five students each. As searching skills are best learned through practice, students should also have access to computers and the internet so they may actively search resources for evidence as part of the lab. Therefore, students should be instructed to bring laptops, or the session should be held in a computer lab. In the most recent cohort at OUWB, a group of 100 students, preassigned in the EBM course to teams of four to five students each, was split among four library faculty members, who taught them simultaneously in separate breakout rooms and also facilitated team activities. Students used laptops to conduct searches.

The facilitator guide (Appendix C) provides a recommended time line for structuring the lab portion of the session. The lab begins with a short, three-question quiz (Appendix D) reviewed as a group. Students are then given a lab worksheet (Appendix E) that they complete as a team. The lab worksheet provides a sample therapy case and an empty PICO table, which teams use to develop a clinical question, search the literature, and record a conclusion about the patient case on the worksheet. The instructor reviews the worksheet with the teams using an instructor key (Appendix F). As part of the EBM course, students must select a topic related to a threaded clinical case and work through the EBM process, culminating in a final team case presentation. Therefore, the second half of the lab is dedicated time for students to work through the first three steps of the EBM process for this case presentation, with library faculty on hand to guide them through framing their topic in the PICO format, developing a clinical question, and searching appropriate resources. A second blank lab worksheet and accompanying key with a diagnosis case (Appendices G & H) have also been included for institutions that wish to use a second sample case instead of having students select their own case.

At the end of the lab, students submit their team worksheets for review, and library faculty provide formative feedback about each team's PICO question, search strategies, and evidence found. Summative evaluation of these elements is included in the evaluation of a team's final case presentation at the end of the course. The final case presentation is graded against a faculty-generated rubric (Appendix I) of six items, three of which directly relate to the skills taught in the lab: a clear, well-built clinical question; a list of databases searched and description of searches performed; and a summary of evidence from each database searched. Each component is graded on a scale of Fails, Below, Meets, or Exceeds Expectations. All course faculty, consisting of an interdisciplinary team of physicians, librarians, and biomedical science faculty with expertise in statistics and epidemiology, serve as judges for the presentations, and presentation grades are discussed and determined through a consensus among judges.

## Results

Since the EBM course debuted in 2013, its structure and requirements, including the “Locating the Best Available Evidence” session, have changed considerably based on student feedback. This session began as a two-part didactic lecture with demonstration and short exercises totaling 4 hours in the first year and was not rated very highly by students. In 2014, the session was revised to the current lecture and lab structure and has been delivered as such for the past three iterations of the course (2014–2016) to a total of 275 second-year medical students. Student evaluations and grading of relevant final case presentation components (PICO questions, search strategies, and overview of evidence found) in 2015 and 2016 have been used to assess the success and effectiveness of this session.

When this session was first offered in 2014, a final case presentation was not included as part of the course requirements, and there was no formal evaluation of the session skills other than a few multiple-choice questions on the final exam. At that time, course evaluations revealed that although students appreciated the interactivity of the session, they did not fully recognize the value of including a searching skills session in the EBM course. There was a general sentiment that the session was redundant with the databases and searching skills taught in their research course during their first year. However, after the implementation of the final case presentation as a graded component of the course in 2015 and 2016, with direct evaluation of the quality of PICO questions, search strategies, and evidence found, student comments on the session were extremely positive. The following themes were identified as things that worked well in the session:

• Library faculty expertise and teaching style.
• Clear and organized introduction to higher evidence resources (6S pyramid).
• Ability to choose to attend the optional lecture or review material at students' leisure prior to the lab.
• Smaller breakout groups that facilitated individualized interactions with library faculty and reduced the distractions of a large class.
• Structure of the lab as an interactive workshop rather than a passive lecture.
• Practical application of searching skills learned in the lab in preparing for the final case presentation as well as for upcoming clerkships the next month.
• Structured, dedicated time during the lab to prepare for the case presentations.
• Prompt formative feedback received from library faculty on each team's PICO question and search strategies well in advance of the final case presentation.

In the last 2 years, students have consistently scored the categories directly related to the lecture and lab session at Meets or Exceeds Expectations. Upon reflection, library faculty have been extremely satisfied with the overall quality of PICO questions, search strategies, and evidence presented in the case presentations, with students demonstrating a level of skill appropriate for entering clerkships or even residency. This assignment has also provided an excellent indicator of students' learned knowledge and skills.

## Discussion

The “Locating the Best Available Evidence” session gets at the heart of the seventh core entrustable professional activity (EPA) outlined by the Association of American Medical Colleges^4^—to prepare medical students in quickly and effectively locating existing evidence to apply to patient care at the time of entering residency. With the revision of the structure of the “Locating the Best Available Evidence” session from a primarily didactic 4-hour lecture into an optional lecture followed by a mandatory 2-hour interactive lab, students have displayed progress toward achieving this EPA through increased awareness and interest in searching EBM resources in the pursuit of evidence-based clinical practice and lifelong learning as future physicians.

Although student feedback indicated that a variety of teaching methods (in-person didactic, online videos, interactive lab) is important, true appreciation of this session only emerged when students perceived an immediate benefit to their learning. More tightly integrating this session into the broader structure of the EBM course and tying it to a graded component of that course ultimately influenced the success of the session. When taught without these connections and with no formal evaluation after the fact, students did not recognize the relevance of the content or value the skills as highly as when they had to present their PICO question and search process as part of a final case presentation. Although the session could be implemented as a stand-alone one, we cannot imagine it being as effective and well received without being tied to other relevant EBM knowledge, skills, and practice. Thus, for the best outcome, it is recommended that this session be delivered with other EBM content, whether in a dedicated course or through a series of integrated sessions across or within courses.

In the future, we would like to conduct a formal evaluation of change in students' knowledge, skills, and attitudes toward EBM resources and EBM searching skills prior to and following this educational intervention. These data, along with analysis of student case presentations and course evaluations, would provide another mechanism for library faculty to continuously evaluate and improve the session.

One additional point of consideration for the design of this session is the PICO model itself. There are a number of variations on this model being used in EBM instruction. In 2006, Haynes, Sackett, Guyatt, and Tugwell expanded the model to include time (T) to account for intervention time frame (PICOT).^16^ The model was expanded further by Samson and Schoelles in 2012 to include study design (S; hence, PICOTS) in the development of systematic reviews for medical tests.^17^ At our institution, the EBM course is the first formal introduction to developing clinical questions in the context of EBM for medical students. Therefore, while creating this session, we decided to use the simpler PICO model and follow the primary source, *Evidence-Based Medicine: How to Practice and Teach It.*^2^ However, other institutions may consider use of the expanded models as they deem appropriate.

No matter whether institutions have a dedicated EBM course or integrate EBM skills throughout the medical school curriculum, this session could easily be adapted and implemented. The overall structure could remain the same, with cases being modified or swapped out to accommodate different specialties or more difficulty for graduate or continuing medical education learners.

## Appendices

A. Locating the Best Available Evidence Lecture-Text.docx
B. Locating the Best Available Evidence Lecture.pptx
C. Lab Facilitator Guide.docx
D. Lab Review Questions.pptx
E. Lab Worksheet Case 1-Blank.docx
F. Lab Worksheet Case 1-Answer Key.docx
G. Lab Worksheet Case 2-Blank.docx
H. Lab Worksheet Case 2-Answer Key.docx
I. Case Presentation Evaluation Rubric.docx All appendices are peer reviewed as integral parts of the Original Publication.
