# Supplementary material for: An Integrated, Case-Based Approach to Teaching Medical Students How to Locate the Best Available Evidence for Clinical Care
Source: MedEdPORTAL. 2017 Jan 19;13:10531. doi: 10.15766/mep_2374-8265.10531 (PMC6342155; doi:10.15766/mep_2374-8265.10531)
Supplement: Supplementary file 1 — A. Locating the Best Available Evidence Lecture-Text.docx B. Locating the Best Available Evidence Lecture.pptx C. Lab Facilitator Guide.docx D. Lab Review Questions.pptx E. Lab Worksheet Case 1-Blank.docx F. Lab Worksheet Case 1-Answer Key.docx G. Lab Worksheet Case 2-Blank.docx H. Lab Worksheet Case 2-Answer Key.docx I. Case Presentation Evaluation Rubric.docx [file mep-13-10531-s001.zip › A. Locating the Best Available Evidence Lecture-Text.docx]

**Locating the Best Available Evidence for Clinical Care**

*Online version available at:* [*https://research.library.oakland.edu/sp/subjects/tutorial.php?faq_id=194*](https://research.library.oakland.edu/sp/subjects/tutorial.php?faq_id=194)

This tutorial walks you through the first two steps of the evidence-based medicine process of 1) **ASK** a pertinent and answerable clinical question and 2) **ACQUIRE** the evidence by selecting and searching the best resource for your question.

--------------------------------------------------------------------------------------------------------------------

**STEP 1: CREATING A SEARCHABLE**

**CLINICAL QUESTION USING PICO**

The reasons for searching the literature and resources appropriate for clinical questions are completely different from what you would use to conduct a literature search for a research project.

Clinical questions are broken down into two types^1^:

- **Background Questions** simply look for background information on a disorder, test, treatment, etc.
  - For example, what is appendicitis?
- **Foreground Questions** are related to a specific patient case or population and are broken down into four components -- PICO:
  - P = Patient or Population
  - I  = Intervention or Exposure
  - C = Comparison intervention or exposure
  - O = Outcome
    - You can use PICO to pick apart a patient case and develop a relevant clinical foreground question such as: How effective is a CT scan compared to ultrasound in diagnosing suspected appendicitis in adult patients?

**STEP 2: USING THE 6S MODEL TO LOCATE THE BEST AVAILABLE EVIDENCE**

Once you have a clear clinical question, it's time to find the best available evidence to answer that question. The 6S Model of pre-appraised, evidence-based medicine resources was developed by DiCenso, Bayley, and Haynes in 2009 to aid clinicians in organizing and remembering the various evidence-based medicine resources available.^2^ This pyramid is divided into 6 tiers, each tier representing a particular type of study and the appropriate resources to find that type of study.


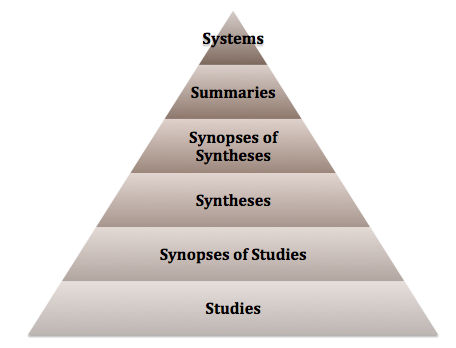


As you move up the pyramid, the level and quality of evidence increases as does the relevance to clinical practice. Since you are most familiar with using PubMed, which falls at the bottom of the evidence pyramid, we'll start at the bottom and work our way up with Studies.

**Studies**


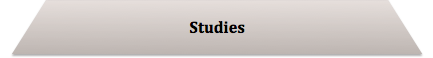
**DEFINITION** Studies are individual studies or original journal articles (aka primary literature)
 **THE EVIDENCE** Studies have not been critically appraised or evaluated by someone else for validity and quality of evidence. Therefore, you must be the one to read it, analyze it, and critique it! It is therefore very difficult to answer your clinical question based on the information you find in Studies --- you get too many results and have no time to critically appraise all of them.
 **KEY RESOURCES FOR LOCATING STUDIES** Many of the databases you use to search for literature for research projects fall into this tier, including PubMed, Web of Science, Scopus, Embase, and even Google Scholar.

The only resource we'll look in this tier is PubMed Clinical Queries (http://www.ncbi.nlm.nih.gov/pubmed/clinical). Clinical Queries provides a quick and easy way to search for clinical studies based on your question type (is it a therapy, diagnosis, etiology, or prognosis question?). It does the work for you in finding relevant AND methodologically sound studies by using pre-set search limits.

*Search Strategies:*The only strategies to searching Clinical Queries are:

1) Make your searches simple (probably no more than 3 - 4 terms)
2) After clicking search, select your category/question type (therapy, diagnosis, etiology, etc) and start your scope as broad


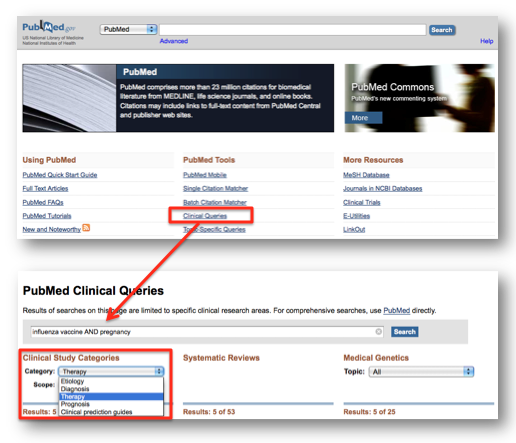


**Synopses of Studies**

**DEFINITION** Synopses o
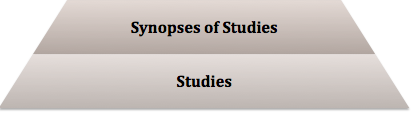
f studies are usually a one-page, structured summary of an individual study with expert commentary. These are only created for a small portion of the literature and are selected to be critically appraised based on their clinical relevance and interest. Therefore, it is good to look at them, but you may not find a synopsis of a study if you have a particular article in mind.

**THE EVIDENCE** Moving up the 6S pyramid, Synopses of Studies provide some expert critical appraisal on the methodology used and the relevance of the article to the field, and therefore a higher level of evidence than Studies, but we're still only looking at a single study.

**KEY RESOURCES FOR LOCATING SYNOPSES OF STUDIES** These types of articles are best found in ACP Journal Club (http://annals.org/journalclub.aspx) and individual evidence-based medicine journals, such as *Evidence-Based Medicine, Evidence-Based Healthcare*, and *Evidence-Based Mental Health*.

**Syntheses**


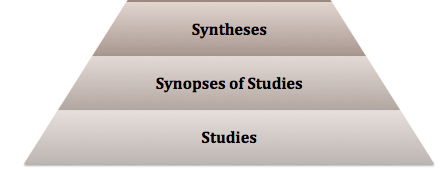
**DEFINITION** Syntheses are systematic reviews or meta-analyses of the literature. According to the Centre for Evidence-Based Medicine in Toronto: "A systematic review is a summary of the medical literature that uses explicit methods to perform a comprehensive literature search and critical appraisal of individual studies and that uses appropriate statistical techniques to combine these valid studies."^3^ Meta-analyses are often considered a type of systematic review where the quantitative data are combined and examined across all single studies included in the study. There are specific and detailed guidelines set by the premier publisher of systematic reviews, the Cochrane Collaboration, on how to appropriately conduct a systematic review of the literature.^4^

**THE EVIDENCE** Since Syntheses systematically search and critically analyze the evidence found across multiple studies, they contain a higher level of evidence than a single study or a commentary of a single study (synopses of studies). With this tier, critical appraisal of the validity and evidence in all the studies contained in a systematic review has already been done for you. Just remember that systematic reviews typically look at a very narrow subset of a particular topic and are not overarching summaries of the literature (we'll be looking at this in Summaries).

**SYSTEMATIC REVIEWS V. NARRATIVE REVIEWS V. LITERATURE REVIEWS** Systematic reviews are often confused with narrative reviews (review articles) or literature reviews. Narrative reviews generally examine the literature from the author's perspective and include author commentary with no criteria for searching and including literature. Literature reviews usually try to identify trends across a subject, do not have a reproducible search or methodology, and do not attempt to appraise the validity of the articles.

**Syntheses, cont.**

**KEY RESOURCES FOR LOCATING SYSTEMATIC REVIEWS**

| **Resource** | **Search Strategies** |
| --- | --- |
| **Cochrane Database of  Systematic Reviews**  *via Cochrane Library* (<http://www.cochranelibrary.com/cochrane-database-of-systematic-reviews/>) | To search just for Cochrane Reviews and not all resources in Cochrane Library:  1. Go to Advanced Search  2. Select Search Limits 3. Check Cochrane Reviews 4. Enter Search Terms |
| **PubMed Health**  (<http://www.ncbi.nlm.nih.gov/pubmedhealth/>) | PubMed Health is the National Library of Medicine's premier database for searching for systematic reviews and includes not only Cochrane reviews, but also reviews from other national and international organizations, such as Agency for Healthcare Research and Quality (AHRQ) and the UK's National Health Service. To search:   1. Enter a few simple search terms into the search box 2. On the right hand side, you can narrow your search with limits such as:    - 'Article Types'    - 'Content Providers' to find reviews published by a specific organization. |
|  |  |

**Synopses of Syntheses**

**DEFINITION** Synopses of Syntheses are exa
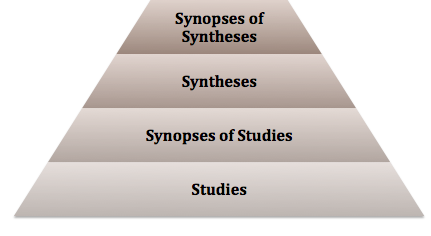
ctly the same as Synopses of Studies, just with systematic reviews instead of single articles. As such, they are usually a one-page, structured summary of a systematic review with expert commentary. Again, not every systematic review or meta-analysis will have been reviewed and evaluated by an expert.

**THE EVIDENCE** Since synopses of syntheses provide some expert critical appraisal on the methodology used and the relevance of a systematic review or meta-analysis to the field, they have a higher level of evidence than systematic reviews.

**KEY RESOURCES FOR LOCATING SYNOPSES OF SYNTHESES** Just like synopses of studies, ACP Journal Club is one resource, however we're going to focus on the Database of Abstracts of Reviews of Effects or DARE. DARE is available via Cochrane Library and freely available in PubMed Health (<http://www.ncbi.nlm.nih.gov/pubmedhealth/>).

*Search Strategies*: Again, there's not a lot of strategy to searching DARE in PubMed Health, except:

1) Make your search simple (probably no more than 3 - 4 terms)
2) Use the limits on the right hand side to select DARE under 'Content Providers' or Reviews from DARE under 'Additional Filters'


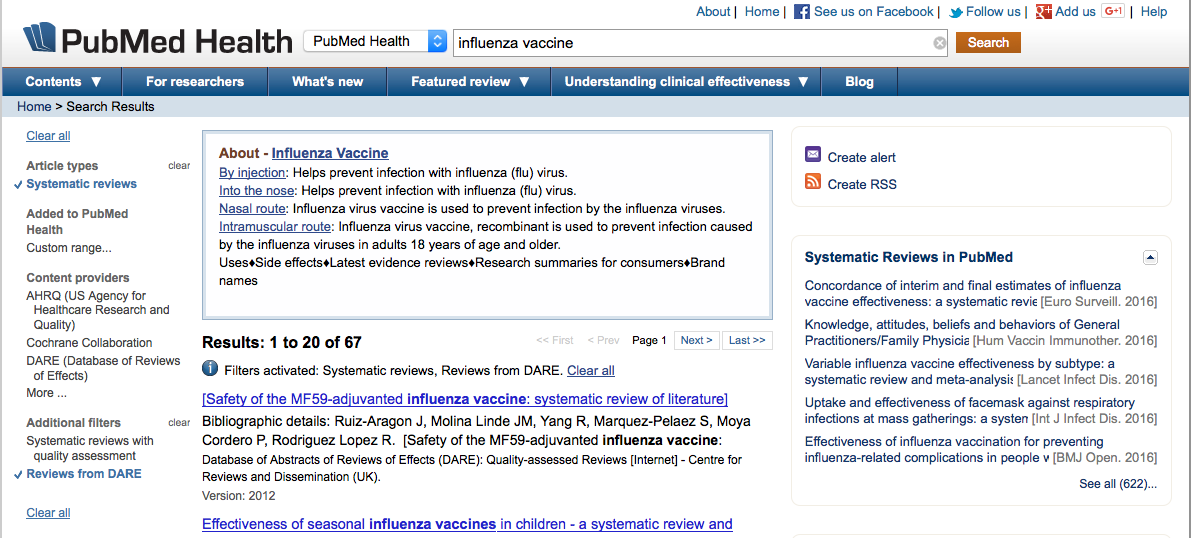


**Summaries**


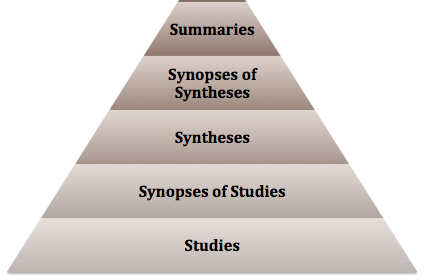
**DEFINITION** Summaries are so-called 'textbook-like' resources that summarize the evidence found in the first four bottom tiers of the 6S Pyramid. As such, they provide a higher level of evidence than those resources below them.
 **THE EVIDENCE** Summaries are probably your best bet for finding the highest levels of evidence for common disease topics as Systems are still evolving (we'll look at this in the Systems module next). However, for new clinical findings and rare cases, you probably will have to search down the pyramid to locate the evidence as either there is not yet enough evidence to warrant a summary or it is too new to have been critically appraised at the various levels.
 **KEY RESOURCES FOR LOCATING SUMMARIES:** Resources that fall into the Summaries category do not have particular search strategies as most are a single Google-like search box. These are marketed and used as point-of-care resources and are, therefore, meant to be quick and easy to search. The two major resources we'll look at are: DynaMed/DynaMed Plus (www.dynamed.com) and UpToDate (www.uptodate.com).

| **Resource** | **Evidence Grading Systems** | **Key Features** |
| --- | --- | --- |
| 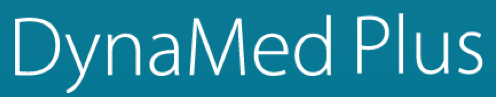 | DynaMed Plus uses a 3-tier evidence grading system to critically  appraise and organize clinical recommendations:   - **Level 1 = Likely reliable evidence** that meet an extensive set of rigorous criteria with minimal bias - **Level 2 = Mid level evidence** that meet some of the rigorous scientific criteria - **Level 3  = Lacking direct evidence** usually representing case reports, case series, and expert opinion   Check out DynaMed's Levels of Evidence Guide (http://dynamed.ebscohost.com/content/LOE) | - Includes 3,200 critically-appraised, evidence-based disease summaries - Claims to update daily (seems unlikely) - All evidence is graded - information presented in bullet form - Mobile app available |
| 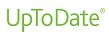 | UpToDate uses the GRADE System^5^ which includes a rating of the recommendation and the evidence supporting that recommendation in UpToDate's clinical summaries:   - Two grades for clinical recommendations   - Grade 1 = strong recommendation   - Grade 2 = weak recommendation - Three levels in each grade describing the quality of evidence   - 1 = high quality evidence   - 2 = moderate quality evidence   - 3 = low quality evidence   Examples of how a grade will appear in a particular topic are Grade 2C, Grade 1A, and so forth. For more information, check out UpToDate's Grading Guide (http://www.uptodate.com/home/grading-guide). | - Includes over 10,000 reference topics written by subject experts (not necessarily critically appraised and evidence-based) - Claims to update daily (seems unlikely) - Slowly applying evidence grading to content - Information presented in paragraph/narrative form - Mobile app available |
|  |  |  |

Clinical practice guidelines also fall into Summaries, however, we won't be covering any of those today. If you want to check them out for yourself, try National Guideline Clearinghouse (http://www.guideline.gov/) or professional organization websites.

**Systems**


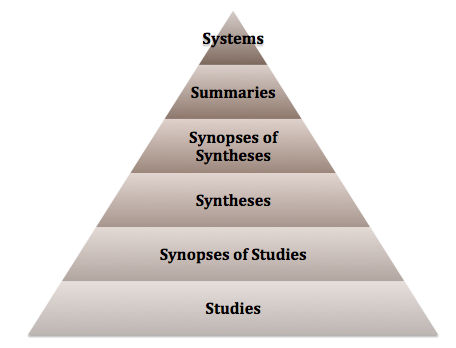
**DEFINITION** Systems are medical information resources that are integrated into electronic medical record systems. For example, UpToDate or Micromedex, a drug information database, are linked right within the patient record so clinicians can simply locate information right from the record rather than accessing these resources externally.

**THE EVIDENCE** Since EMRs are still not universal in the U.S. and many are still in the early implementation stages, it is very rare to find integrated resources. Therefore, although it falls as the highest evidence on the 6S pyramid, most likely you will be starting with Summaries when searching for evidence.

**Conclusion - Locating the Best Evidence**

To conclude, when trying to locate the best available evidence for your patient and clinical care in general, there are numerous resources out there to choose from. The key is using the 6S Pyramid as one means of organizing these resources in a memorable and meaningful way to help you find the evidence quickly and efficiently.

The following image recaps the major resources at each level of the pyramid and reminds you to start from the top when searching for the evidence.


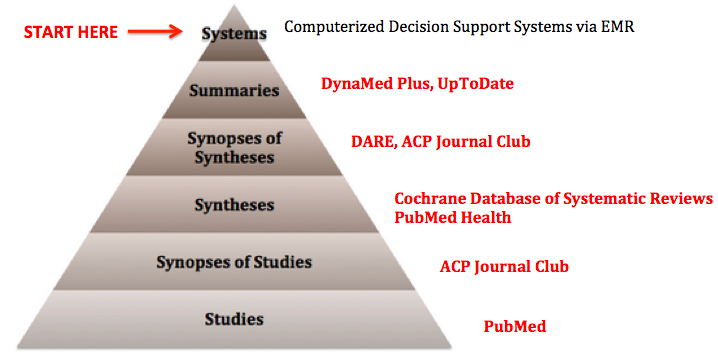


Two emerging resources that attempt to search across all of the tiers of the 6S Pyramid are ACCESSSS (<http://plus.mcmaster.ca/ACCESSSS/>) from McMaster University and the Trip Database (http://www.tripdatabase.com/). Both are freely available online and link to subscription and free online content. These resources are still being developed, but are worth taking a look at when you have a chance.

**References**

^1^ Straus SE, Glasziou P, Richardson WS, Haynes RB. Asking answerable clinical questions. In: *Evidence-based medicine: how to practice and teach it*. 4th ed. Edinburgh, UK: Elsevier Churchill Livingstone; 2011:13–27.

^2^DiCenso A, Bayley L, Haynes RB. Accessing pre-appraised evidence: fine-tuning the 5S model into a 6S model. *Evid Based Nurs*. 2009;12(4):99-101.

^3^Centre for Evidence-Based Medicine. Glossary of EBM Terms. <http://ktclearinghouse.ca/cebm/glossary/> Accessed August 12, 2016.

^4^Cochrane Collaboration. Cochrane Handbook for Systematic Reviews of Interventions. <http://handbook.cochrane.org/> Accessed August 12, 2016

^5^Kavanagh BP. The GRADE system for rating clinical guidelines. *PLoS Med*. 2009;6(9):e1000094.
