# Supplementary material for: An Integrated, Case-Based Approach to Teaching Medical Students How to Locate the Best Available Evidence for Clinical Care
Source: MedEdPORTAL. 2017 Jan 19;13:10531. doi: 10.15766/mep_2374-8265.10531 (PMC6342155; doi:10.15766/mep_2374-8265.10531)
Supplement: Supplementary file 1 — A. Locating the Best Available Evidence Lecture-Text.docx B. Locating the Best Available Evidence Lecture.pptx C. Lab Facilitator Guide.docx D. Lab Review Questions.pptx E. Lab Worksheet Case 1-Blank.docx F. Lab Worksheet Case 1-Answer Key.docx G. Lab Worksheet Case 2-Blank.docx H. Lab Worksheet Case 2-Answer Key.docx I. Case Presentation Evaluation Rubric.docx [file mep-13-10531-s001.zip › B. Locating the Best Available Evidence Lecture.pptx]

## Slide 1
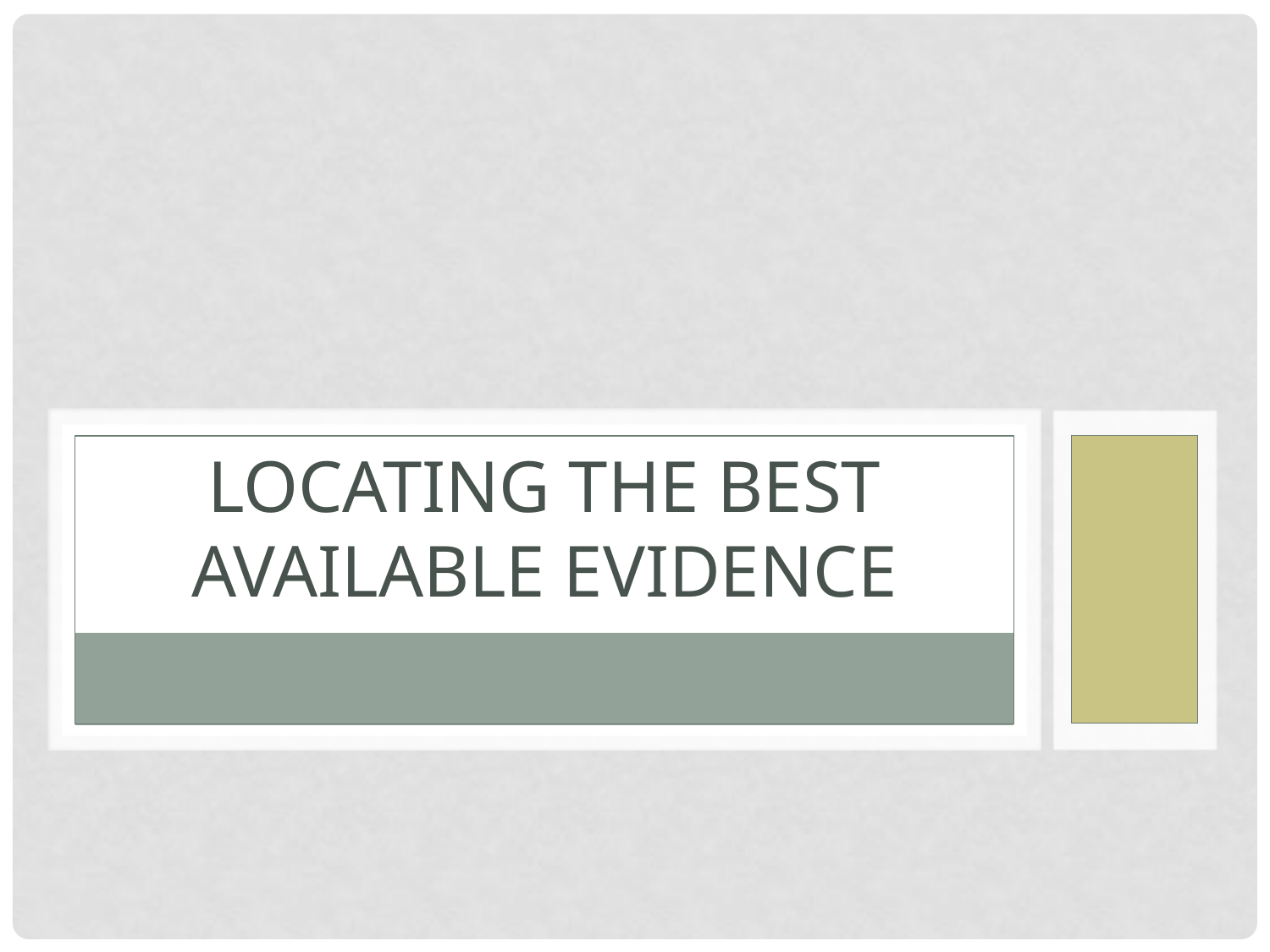

# Locating the Best Available Evidence

## Slide 2
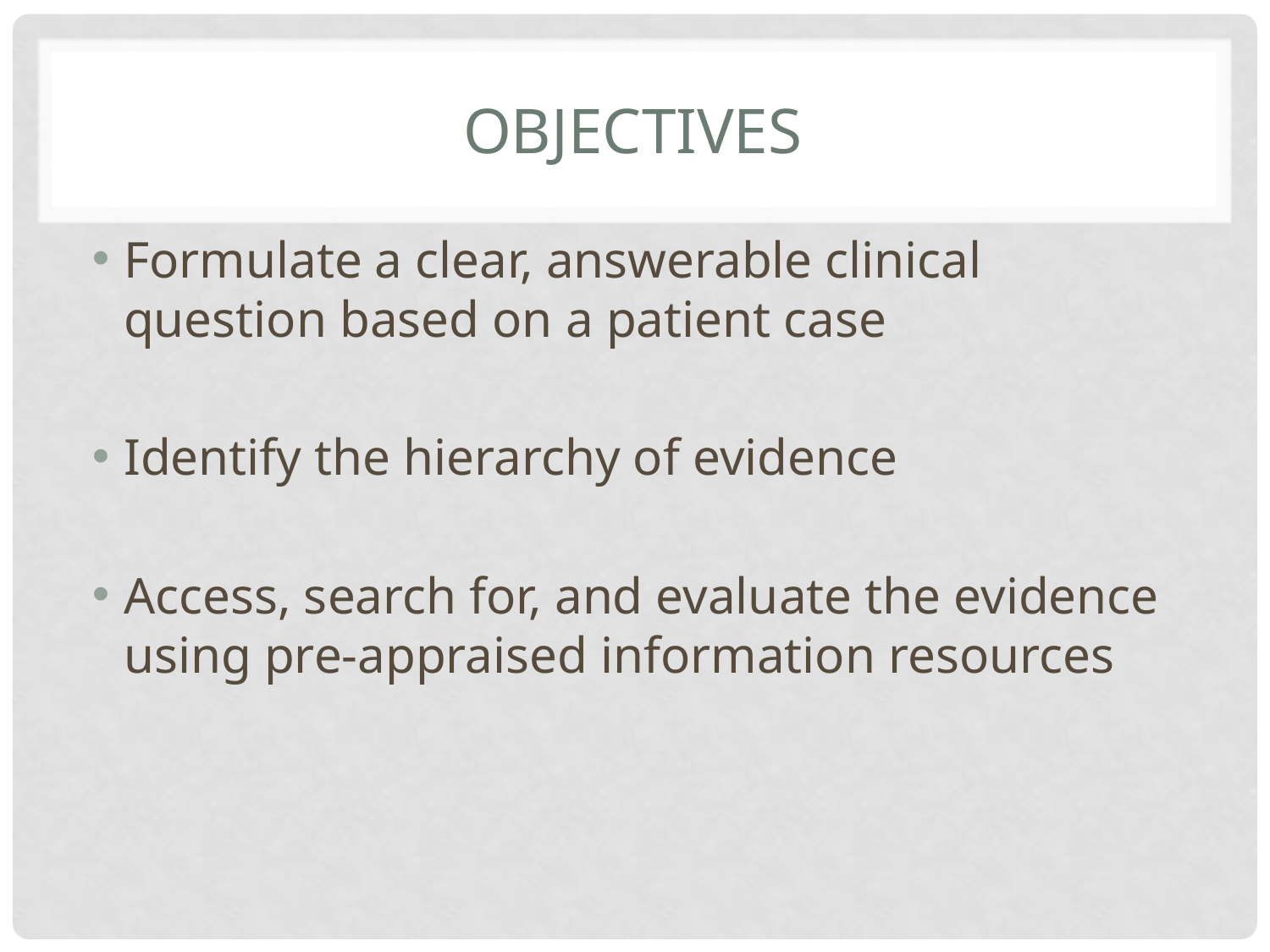

# Objectives
Formulate a clear, answerable clinical question based on a patient case
Identify the hierarchy of evidence
Access, search for, and evaluate the evidence using pre-appraised information resources

## Slide 3
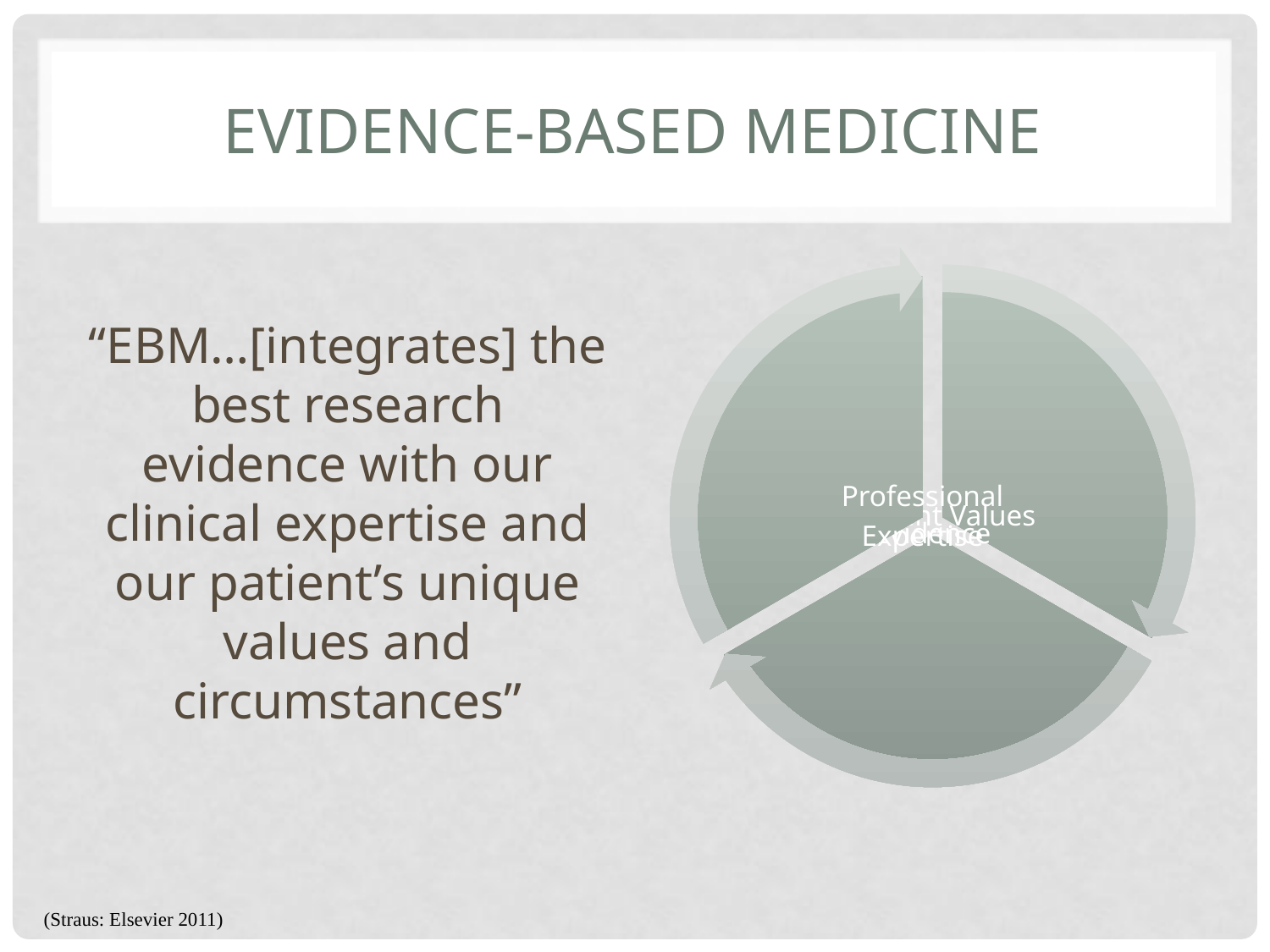

# Evidence-based Medicine
“EBM…[integrates] the best research evidence with our clinical expertise and our patient’s unique values and circumstances”
(Straus: Elsevier 2011)

## Slide 4
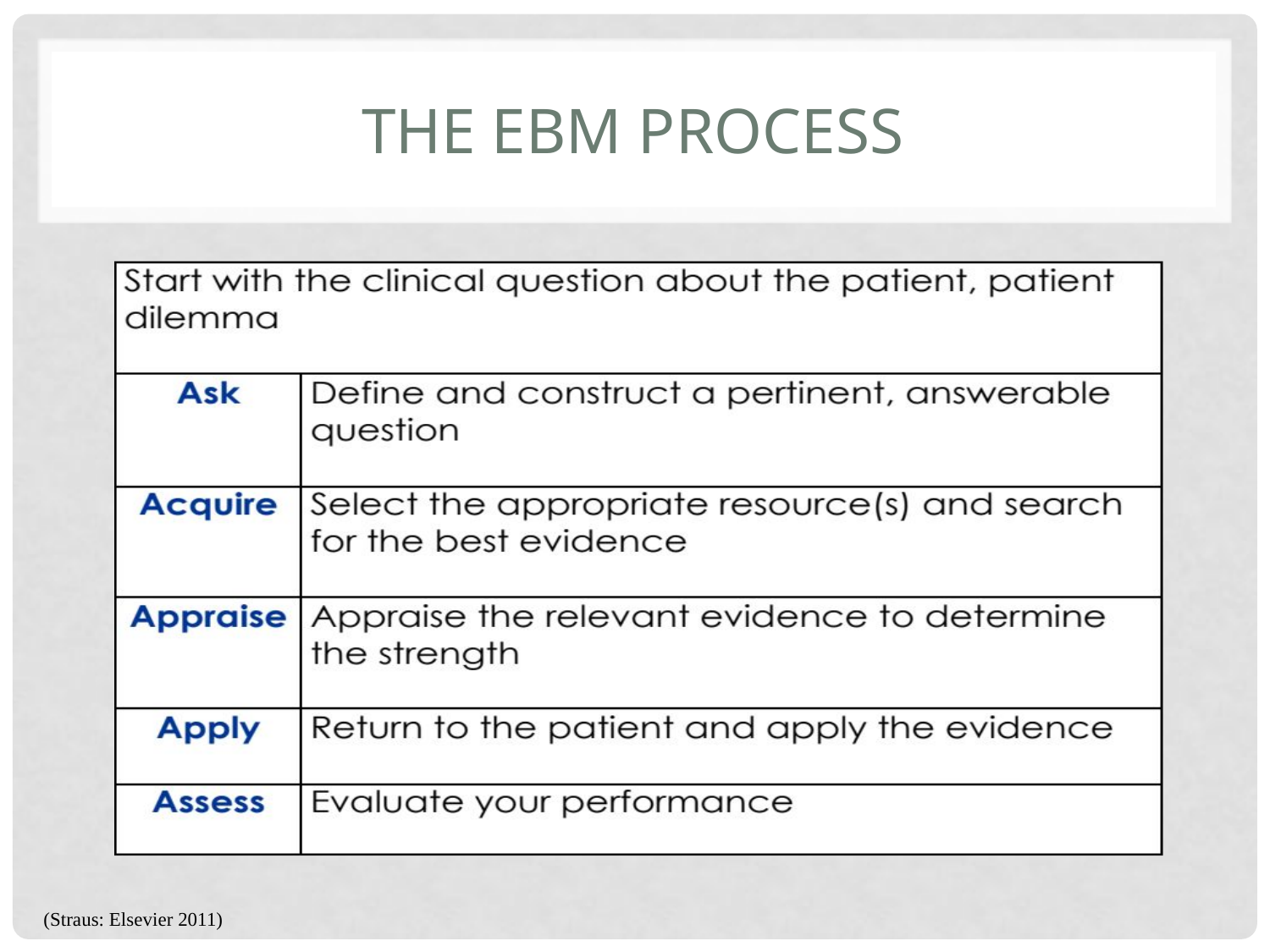

# The EBM Process
(Straus: Elsevier 2011)

## Slide 5
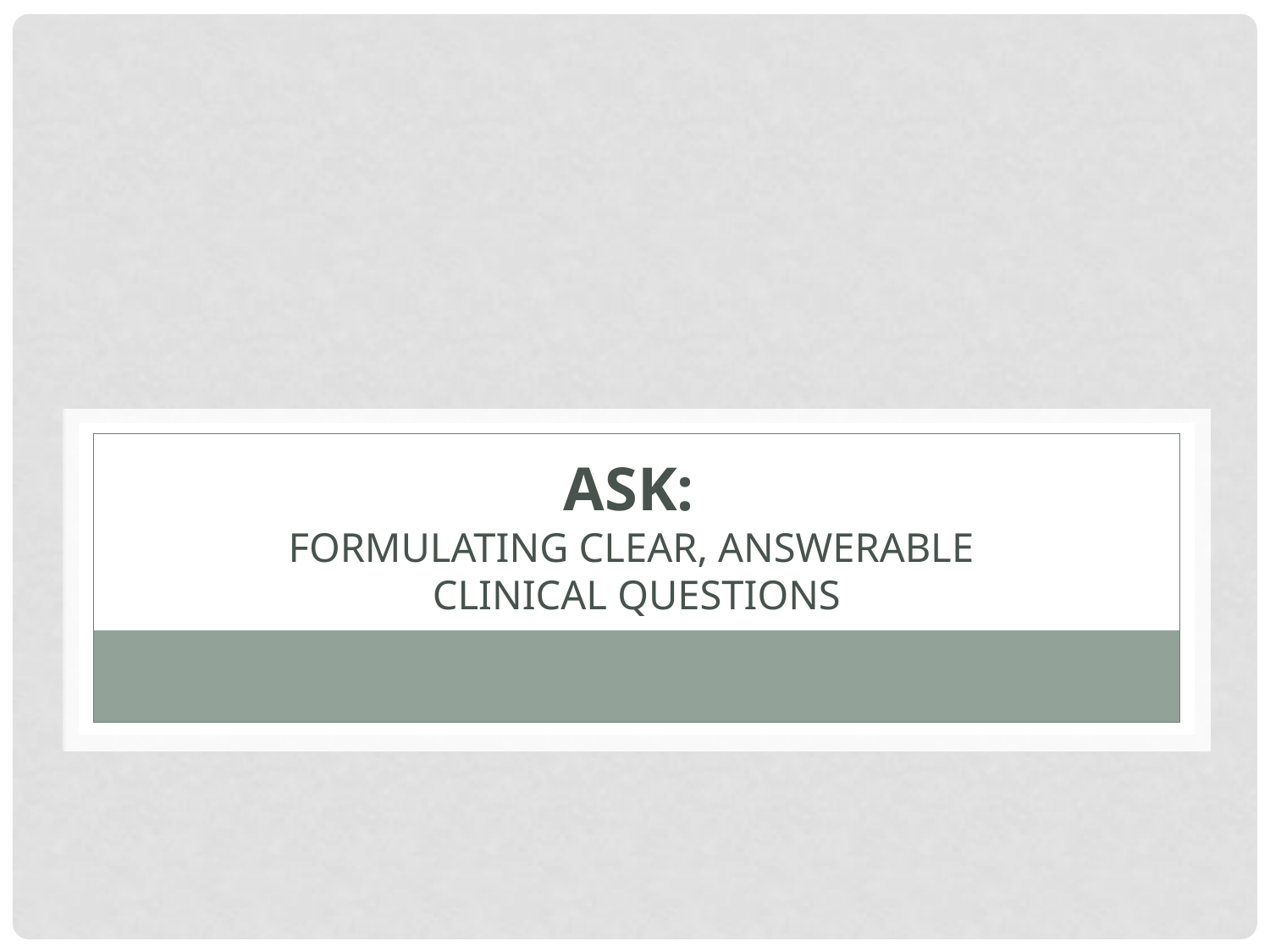

# ASK: formulating clear, answerable Clinical questions

## Slide 6
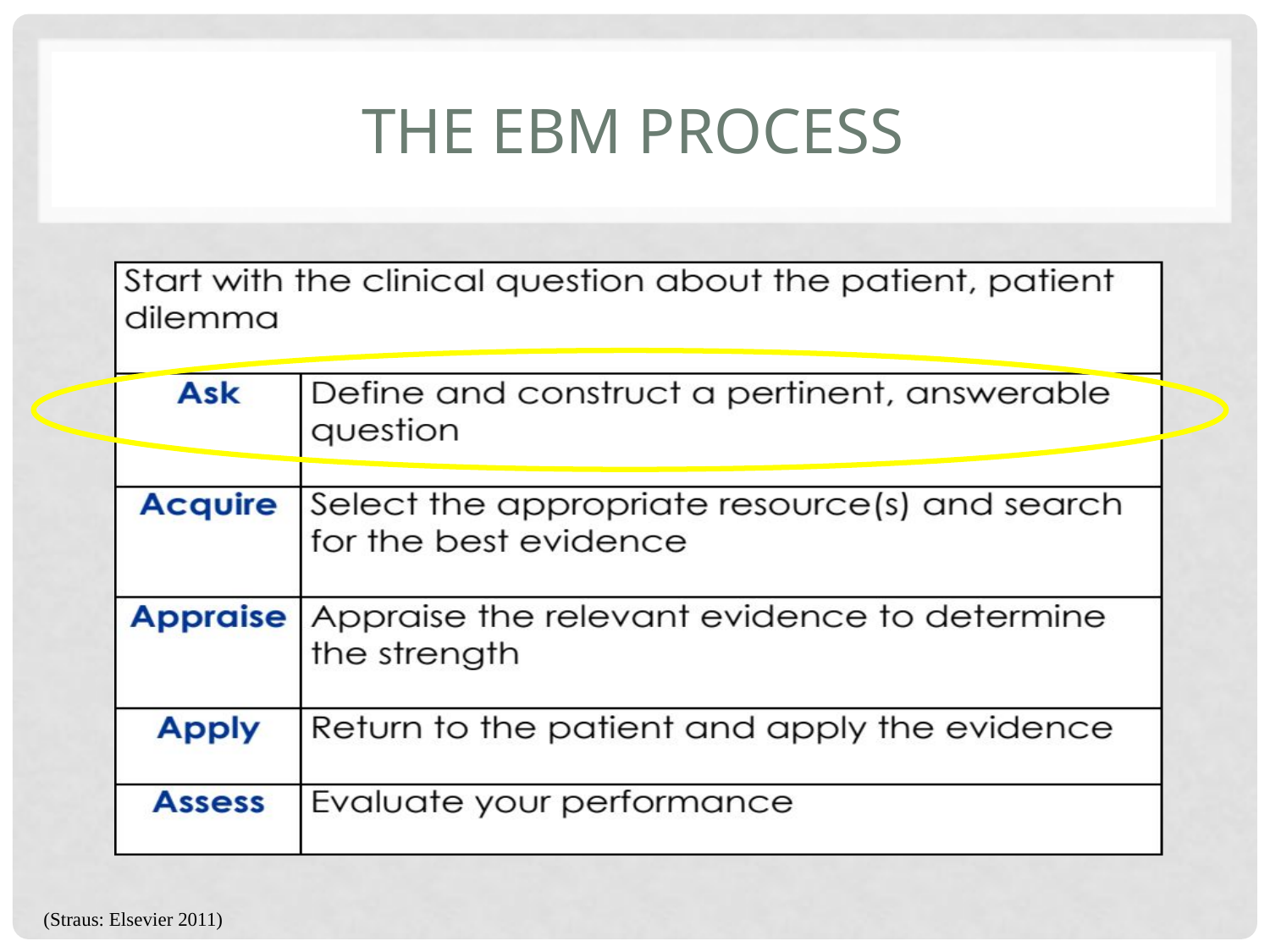

# The EBM Process
(Straus: Elsevier 2011)

## Slide 7
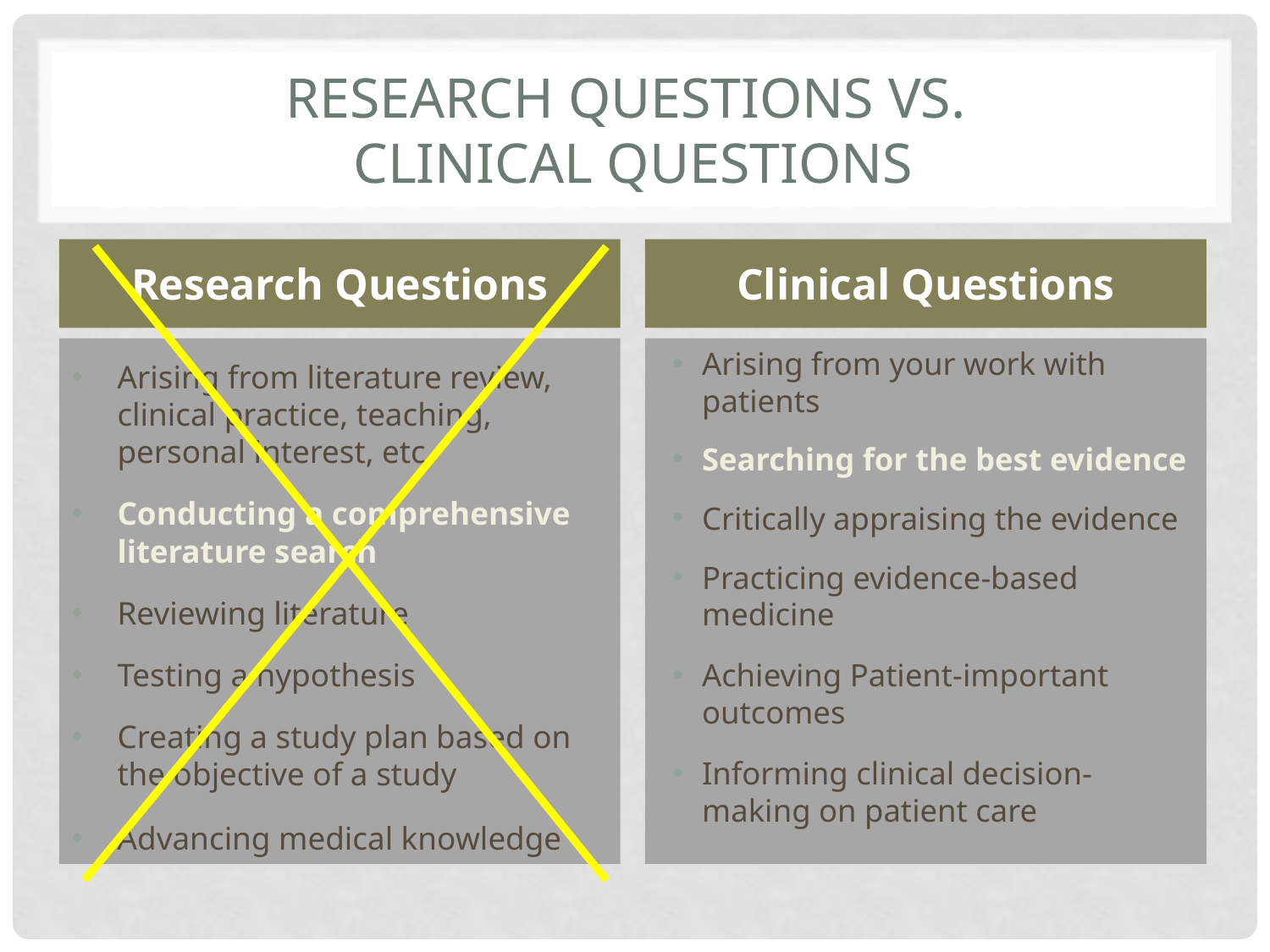

# Research Questions vs. Clinical Questions
Research Questions
Clinical Questions
Arising from your work with patients
Searching for the best evidence
Critically appraising the evidence
Practicing evidence-based medicine
Achieving Patient-important outcomes
Informing clinical decision-making on patient care
Arising from literature review, clinical practice, teaching, personal interest, etc
Conducting a comprehensive literature search
Reviewing literature
Testing a hypothesis
Creating a study plan based on the objective of a study
Advancing medical knowledge

## Slide 8
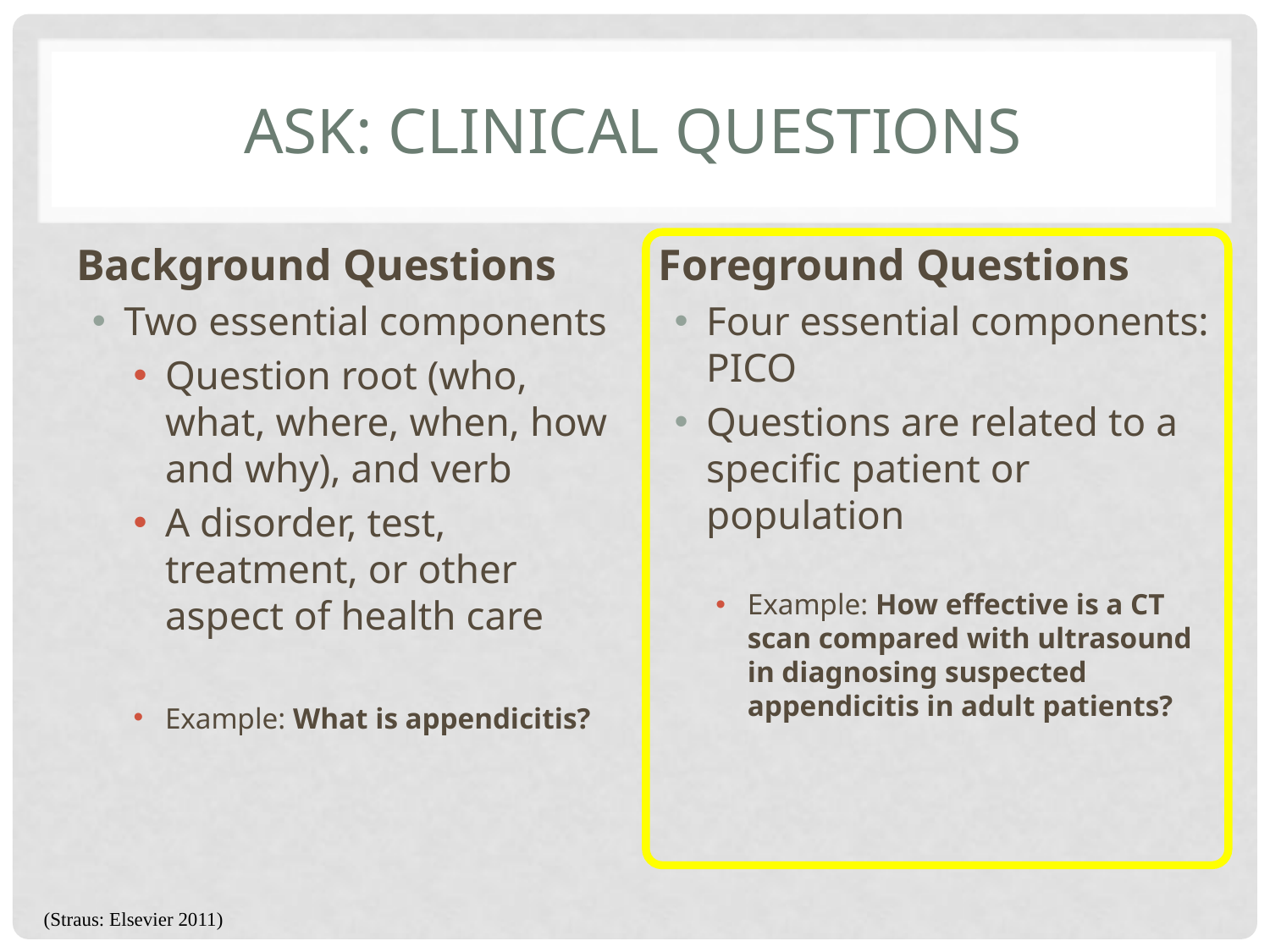

# ASK: Clinical Questions
Background Questions
Two essential components
Question root (who, what, where, when, how and why), and verb
A disorder, test, treatment, or other aspect of health care
Example: What is appendicitis?
Foreground Questions
Four essential components: PICO
Questions are related to a specific patient or population
Example: How effective is a CT scan compared with ultrasound in diagnosing suspected appendicitis in adult patients?
(Straus: Elsevier 2011)

## Slide 9
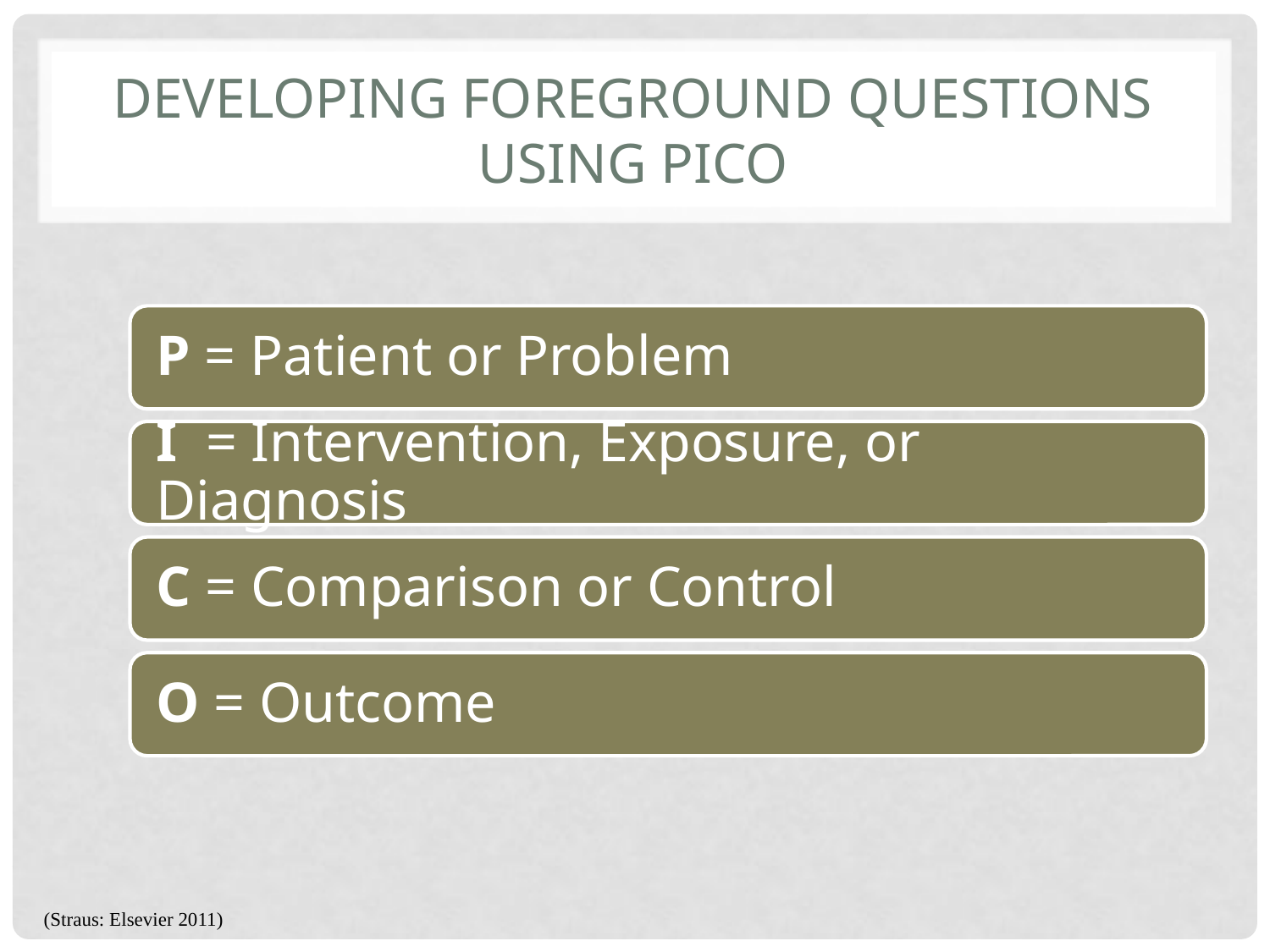

# Developing Foreground Questions using PICO
(Straus: Elsevier 2011)

## Slide 10
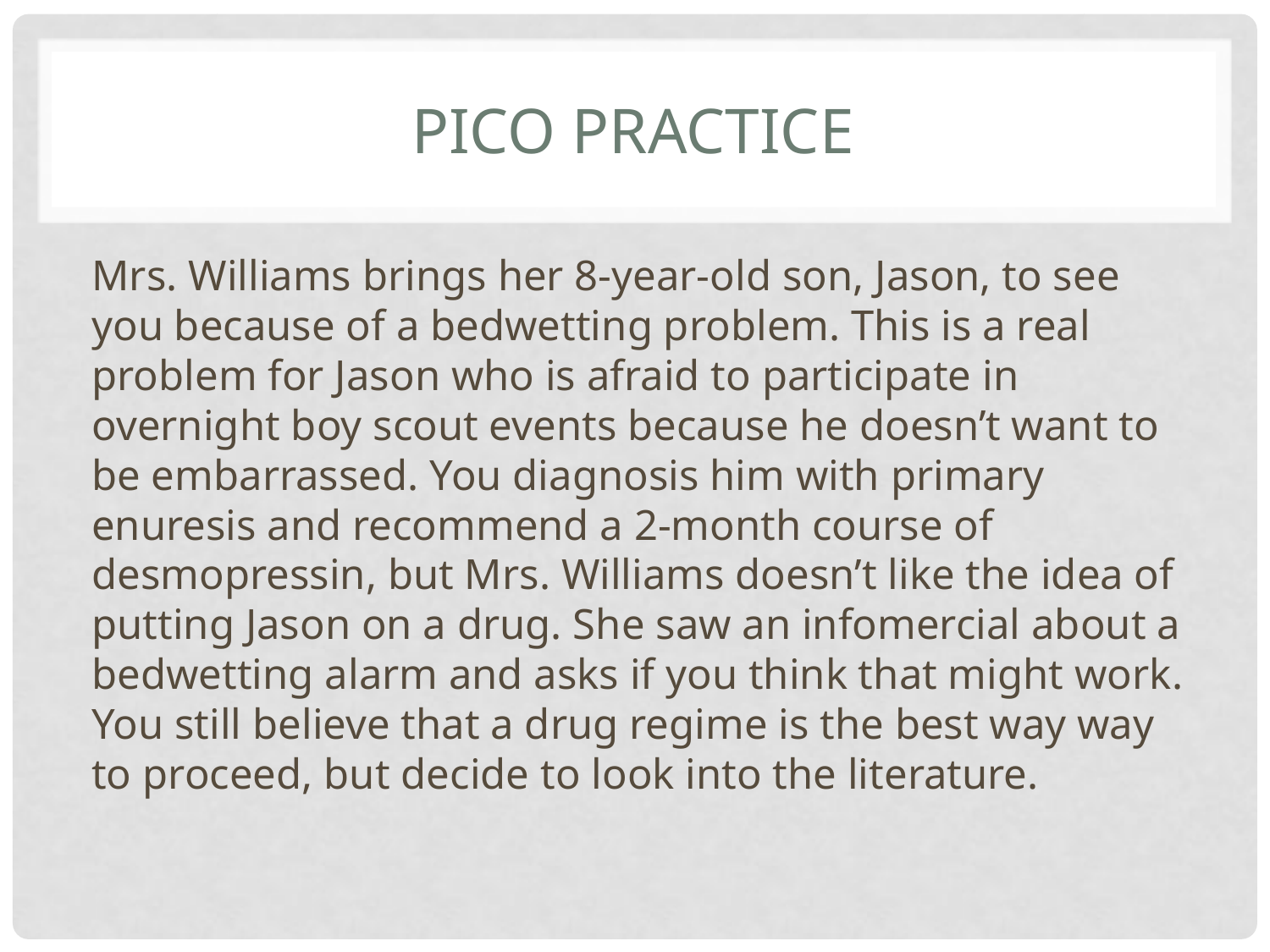

# PICO Practice
Mrs. Williams brings her 8-year-old son, Jason, to see you because of a bedwetting problem. This is a real problem for Jason who is afraid to participate in overnight boy scout events because he doesn’t want to be embarrassed. You diagnosis him with primary enuresis and recommend a 2-month course of desmopressin, but Mrs. Williams doesn’t like the idea of putting Jason on a drug. She saw an infomercial about a bedwetting alarm and asks if you think that might work. You still believe that a drug regime is the best way way to proceed, but decide to look into the literature.

## Slide 11
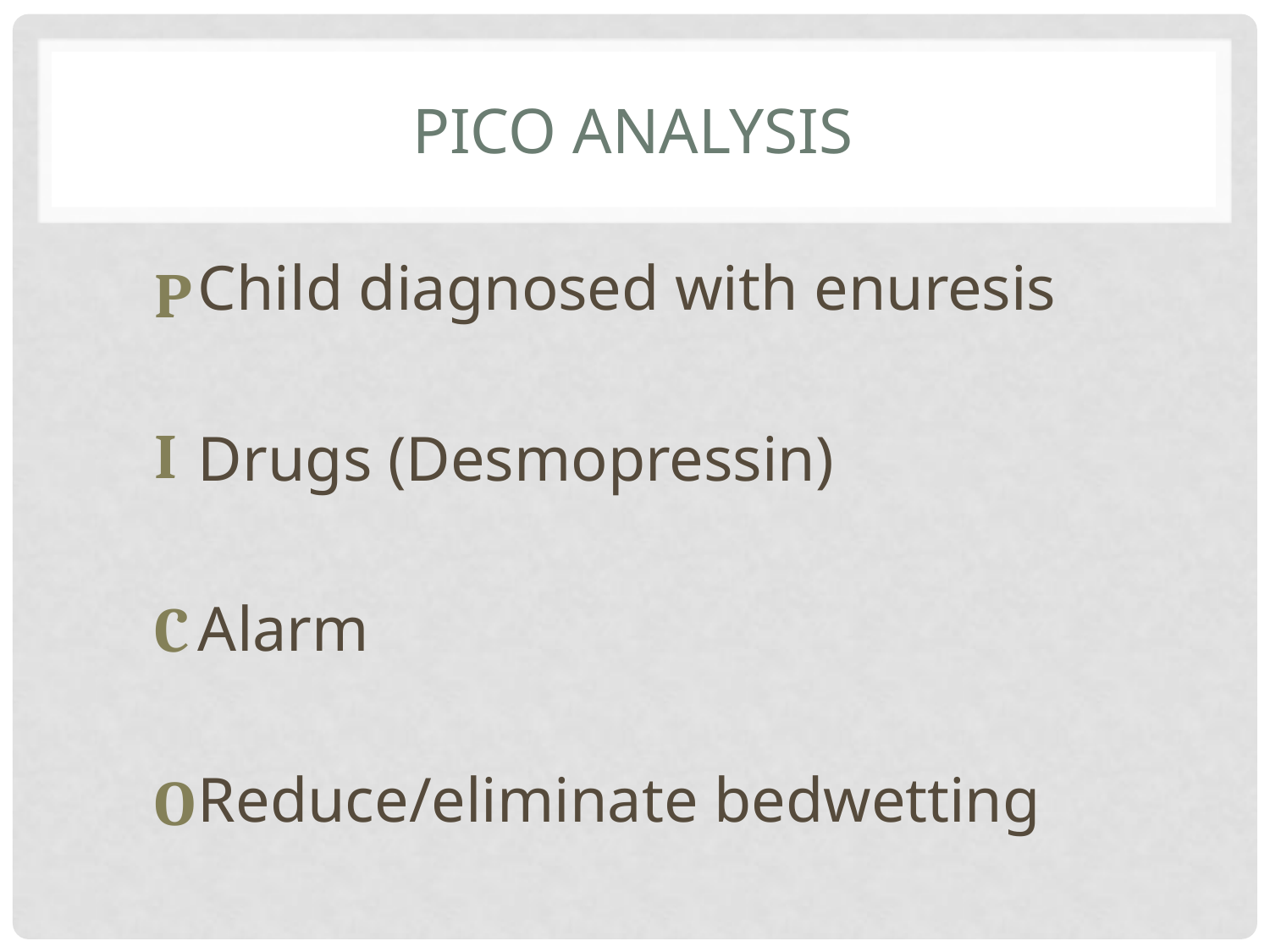

# PICO Analysis
		Child diagnosed with enuresis
		Drugs (Desmopressin)
		Alarm
		Reduce/eliminate bedwetting
P
I
C
O

## Slide 12
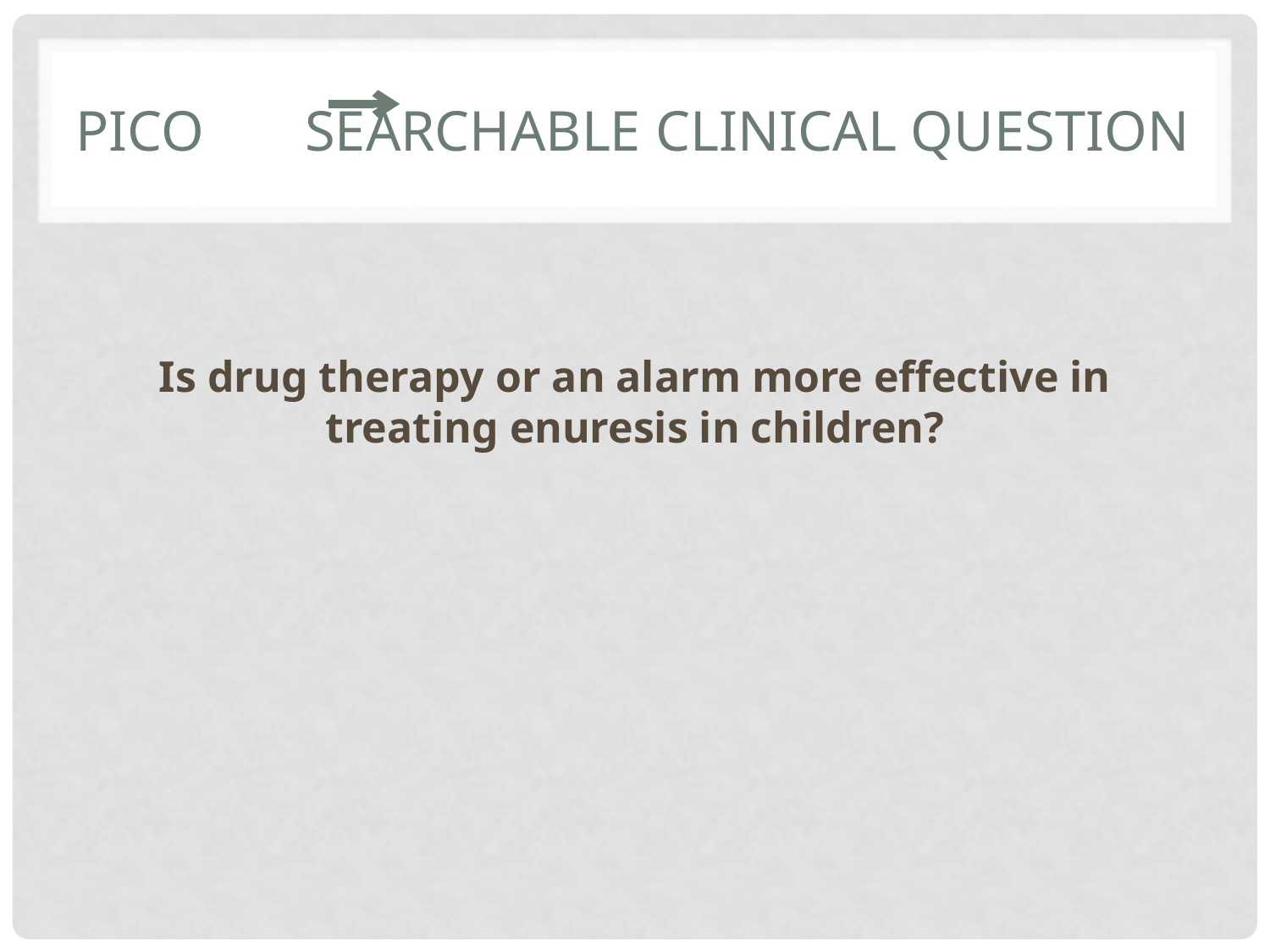

# PICO Searchable Clinical Question
Is drug therapy or an alarm more effective in treating enuresis in children?

## Slide 13
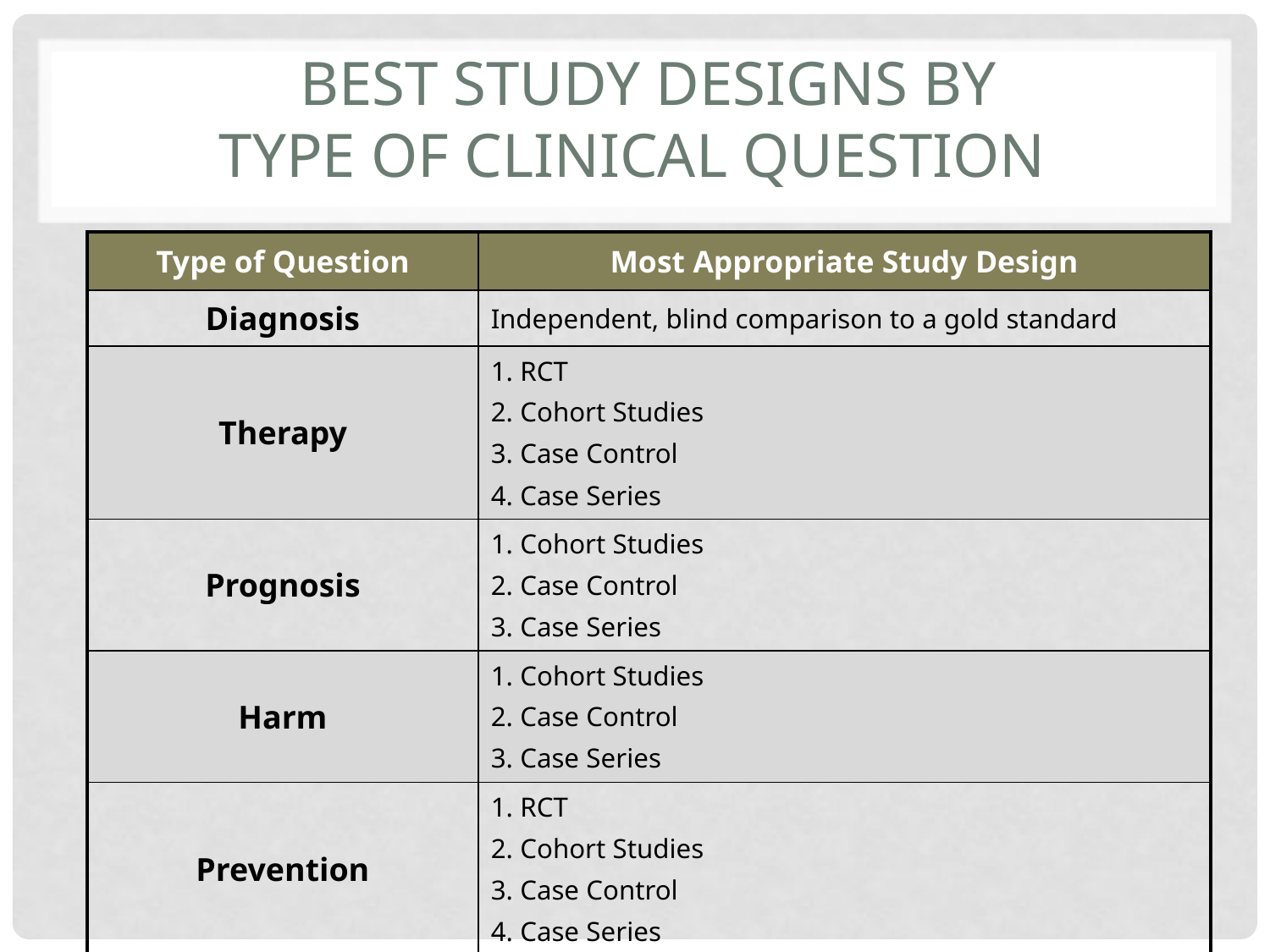

# Best Study Designs By Type of Clinical Question
| Type of Question | Most Appropriate Study Design |
| --- | --- |
| Diagnosis | Independent, blind comparison to a gold standard |
| Therapy | 1. RCT 2. Cohort Studies 3. Case Control 4. Case Series |
| Prognosis | 1. Cohort Studies 2. Case Control 3. Case Series |
| Harm | 1. Cohort Studies 2. Case Control 3. Case Series |
| Prevention | 1. RCT 2. Cohort Studies 3. Case Control 4. Case Series |

## Slide 14
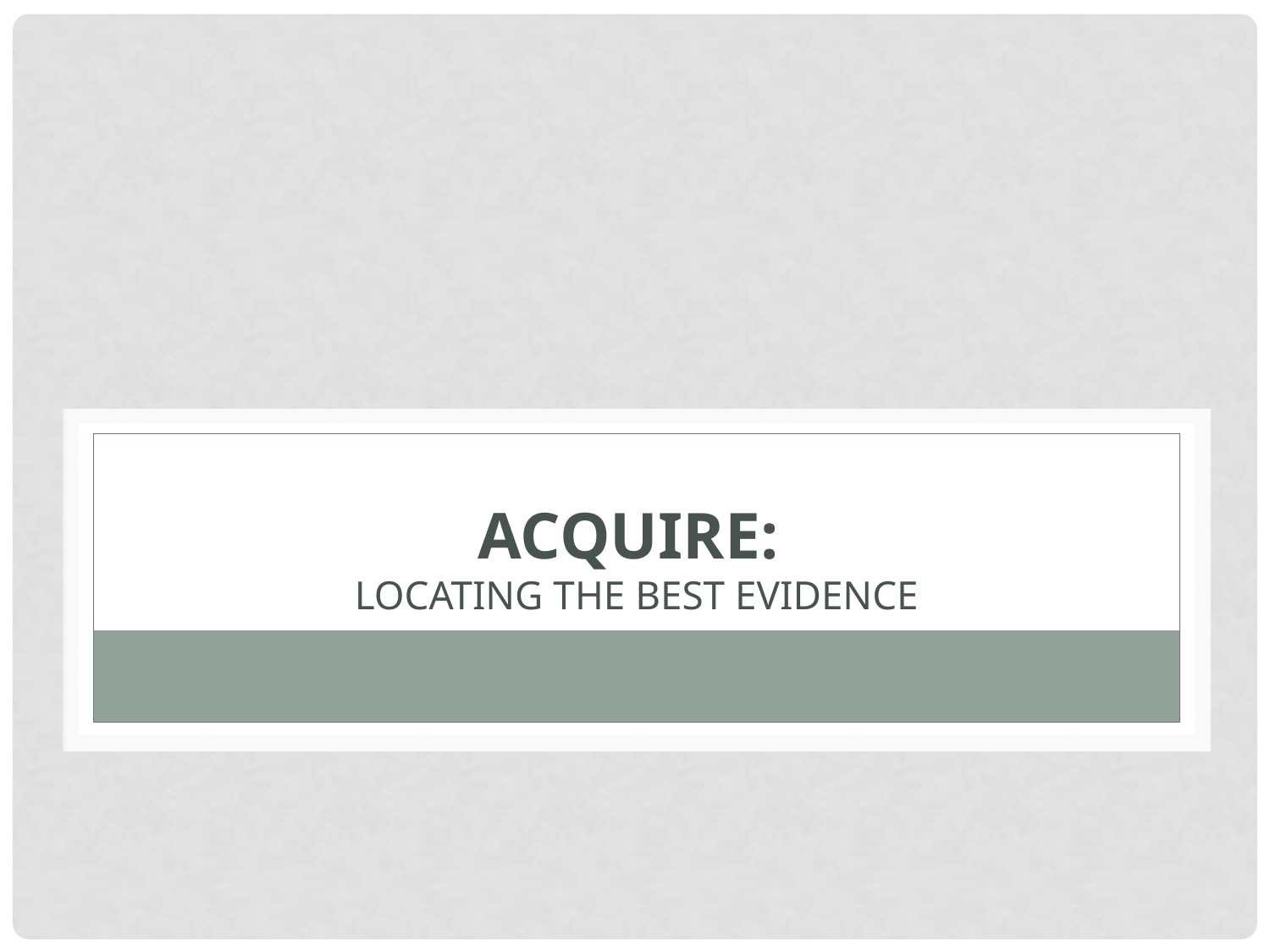

# ACQUIRE: locating the best evidence

## Slide 15
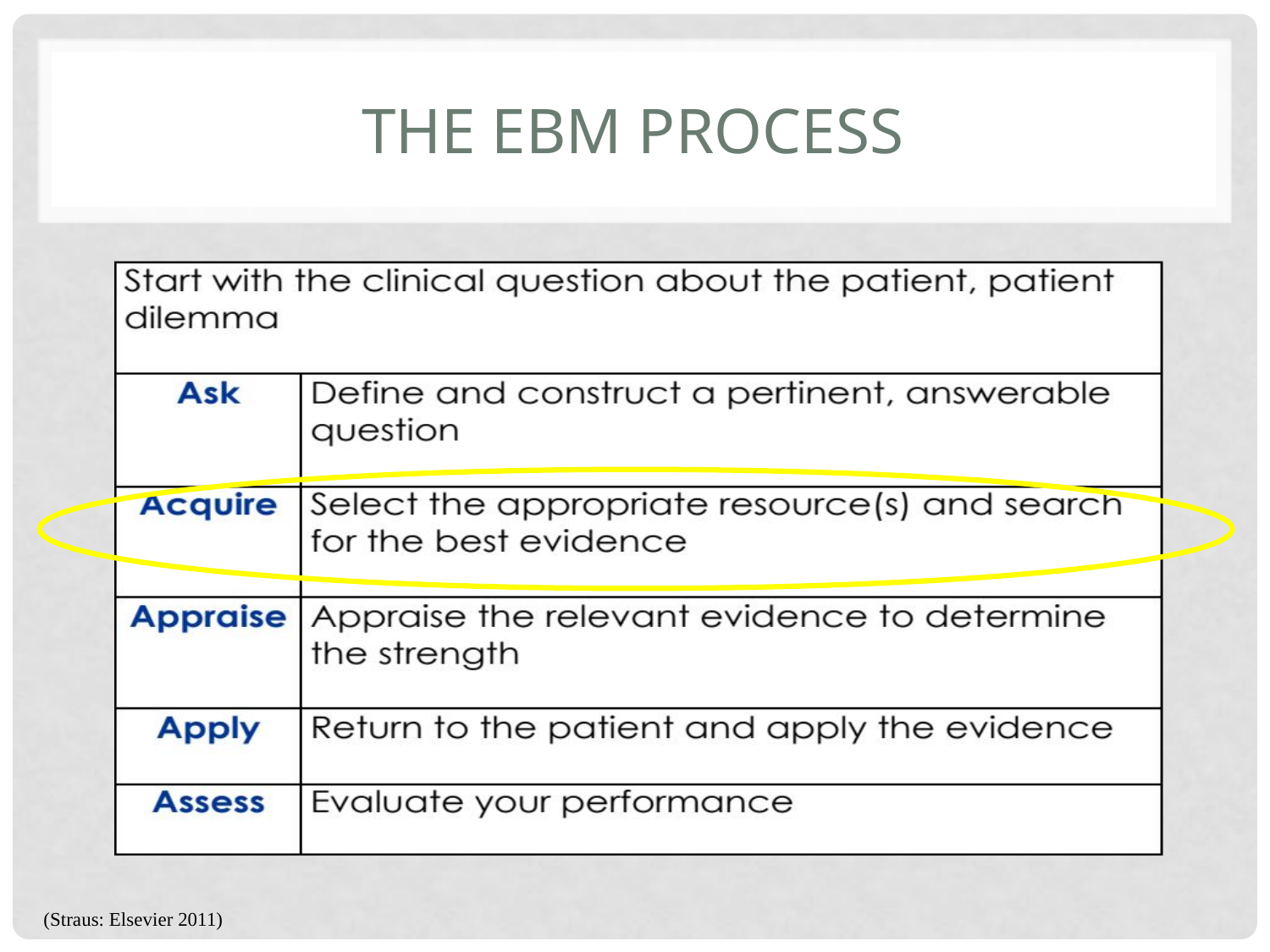

# The EBM Process
(Straus: Elsevier 2011)

## Slide 16
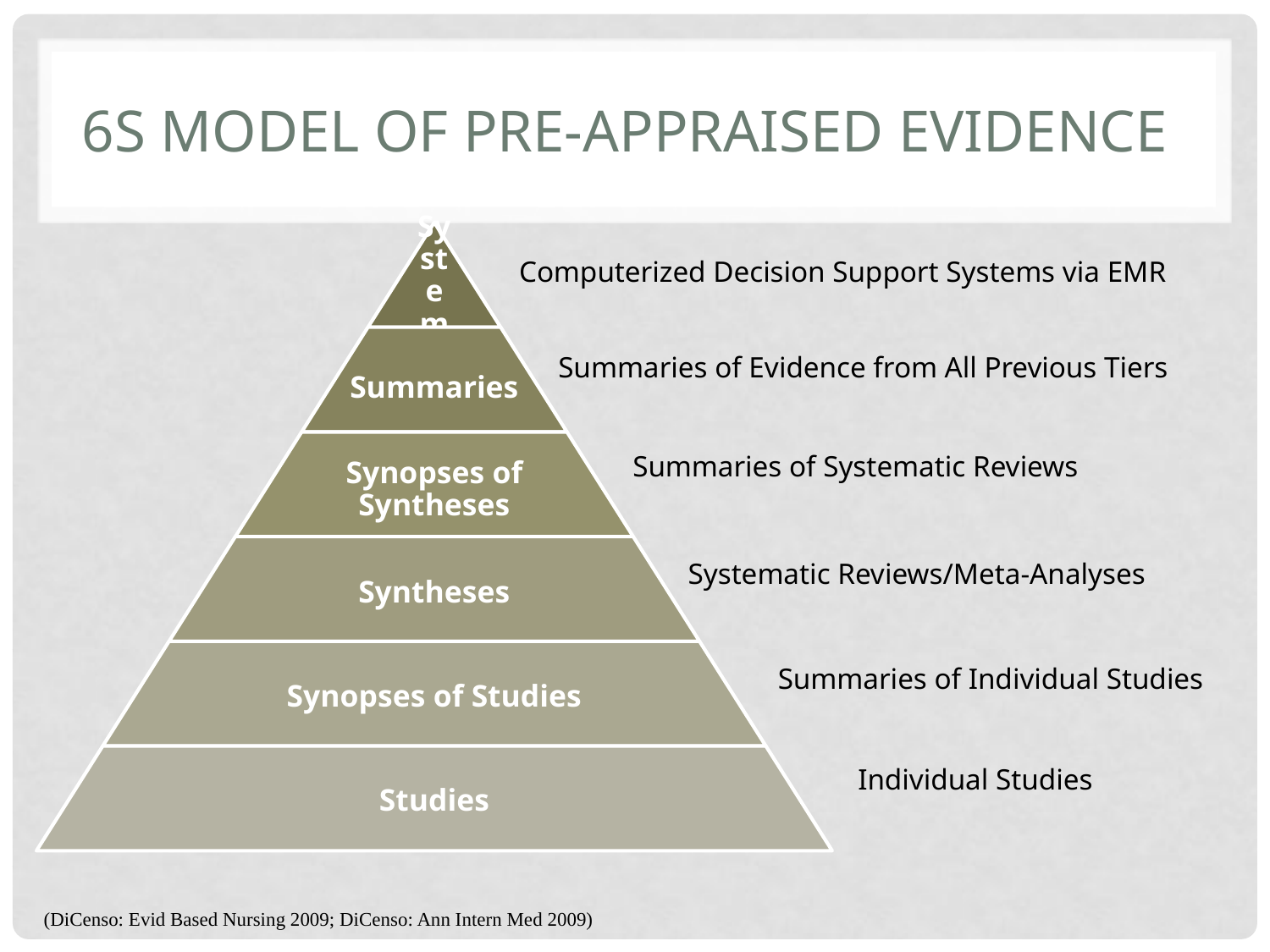

# 6S Model of Pre-Appraised Evidence
Computerized Decision Support Systems via EMR
Summaries of Evidence from All Previous Tiers
Summaries of Systematic Reviews
Systematic Reviews/Meta-Analyses
Summaries of Individual Studies
Individual Studies
(DiCenso: Evid Based Nursing 2009; DiCenso: Ann Intern Med 2009)

## Slide 17
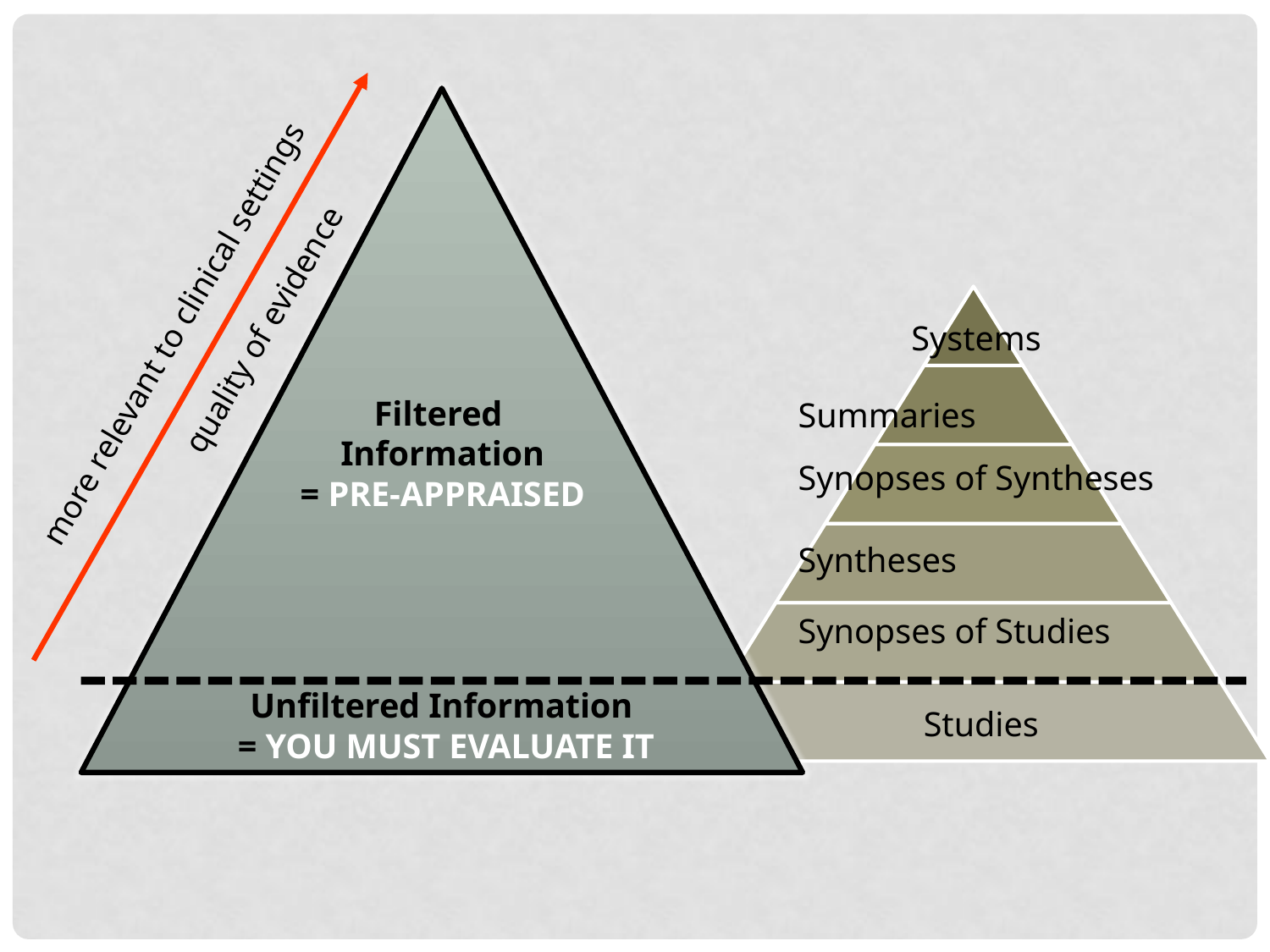

more relevant to clinical settings
 quality of evidence
Systems
Summaries
Synopses of Syntheses
Syntheses
Synopses of Studies
Filtered
 Information
= PRE-APPRAISED
Unfiltered Information
= YOU MUST EVALUATE IT
Studies

## Slide 18
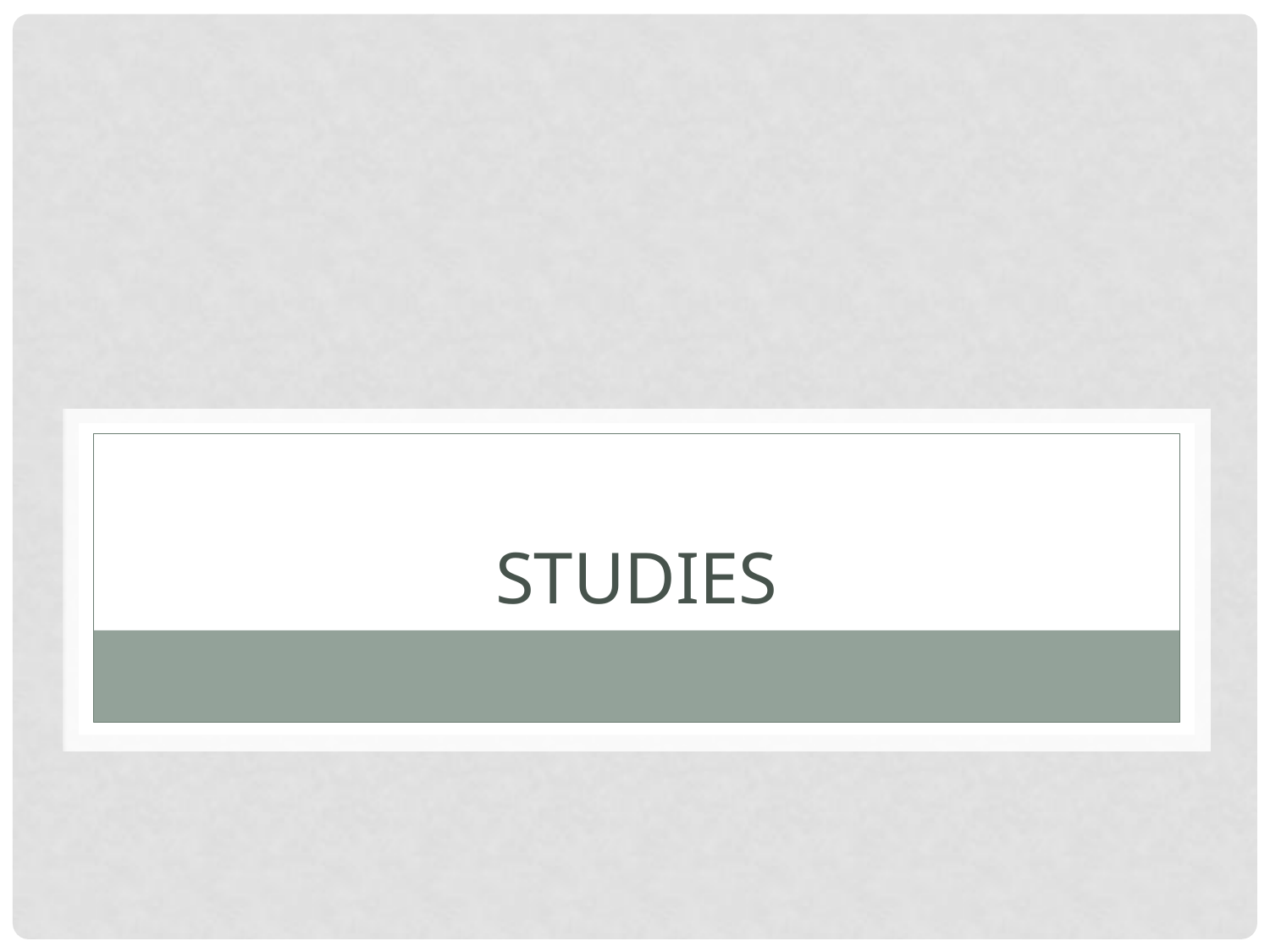

# STUDIES

## Slide 19
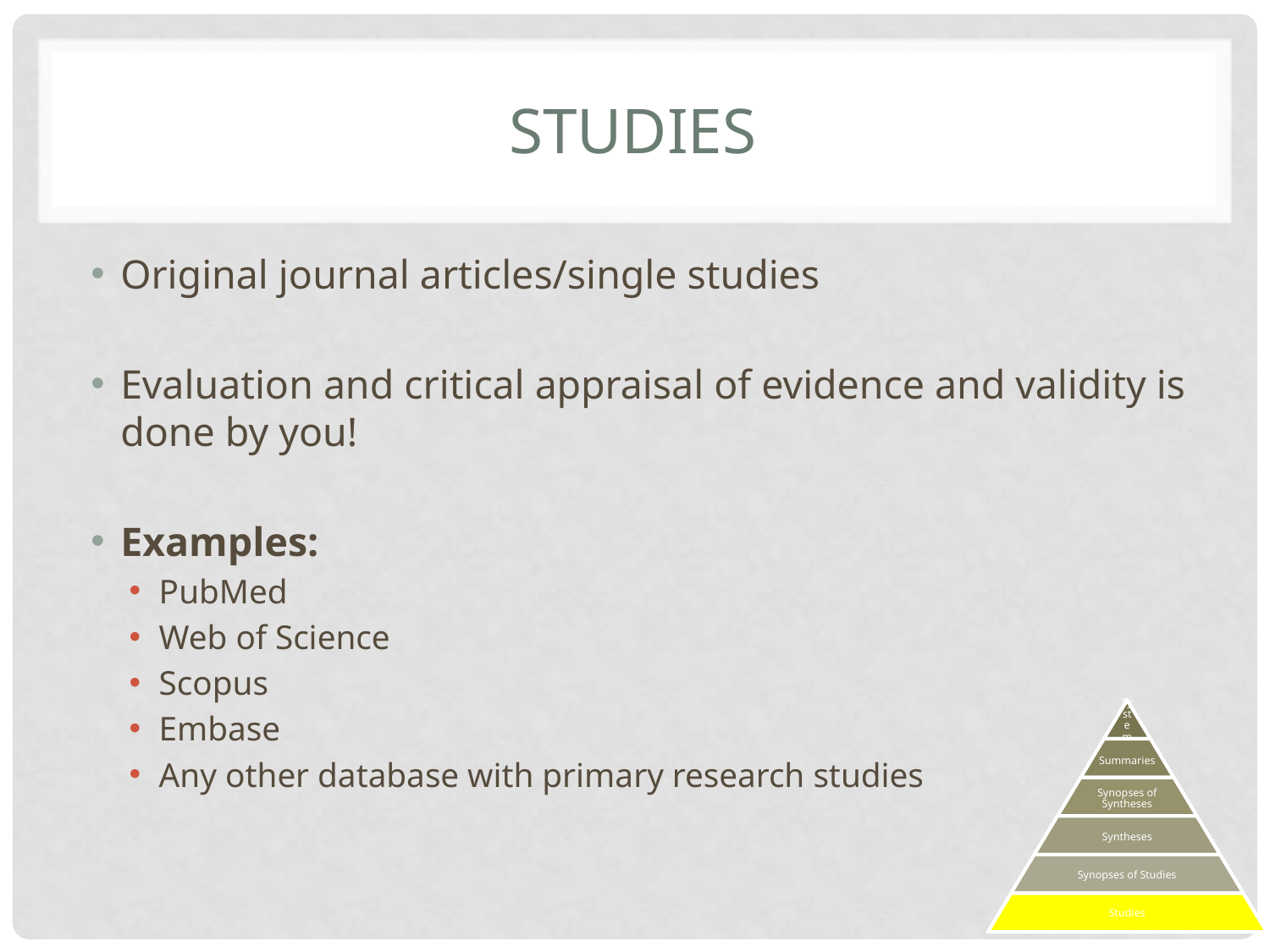

# Studies
Original journal articles/single studies
Evaluation and critical appraisal of evidence and validity is done by you!
Examples:
PubMed
Web of Science
Scopus
Embase
Any other database with primary research studies

## Slide 20
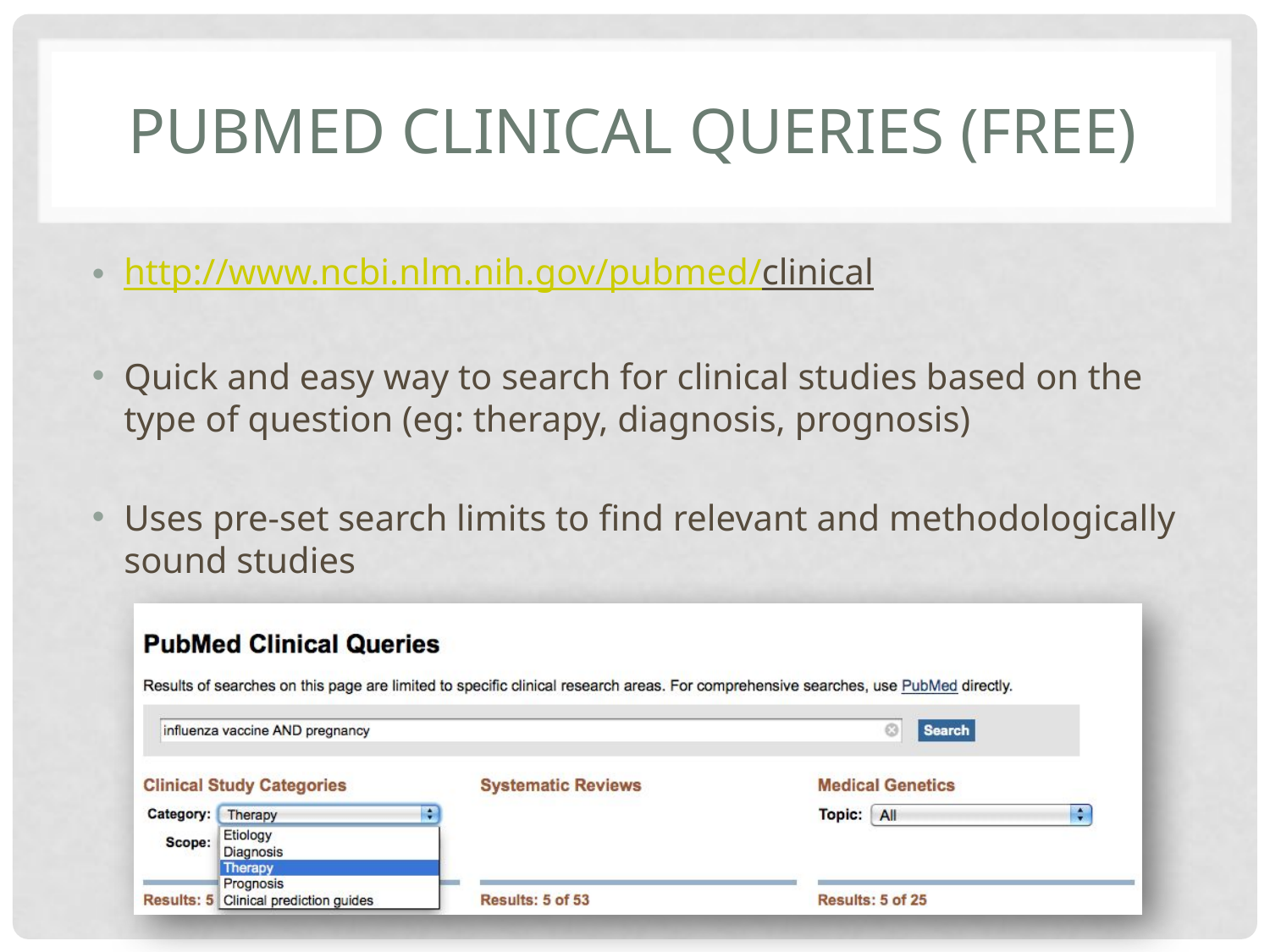

# PubMed Clinical Queries (free)
http://www.ncbi.nlm.nih.gov/pubmed/clinical
Quick and easy way to search for clinical studies based on the type of question (eg: therapy, diagnosis, prognosis)
Uses pre-set search limits to find relevant and methodologically sound studies

## Slide 21
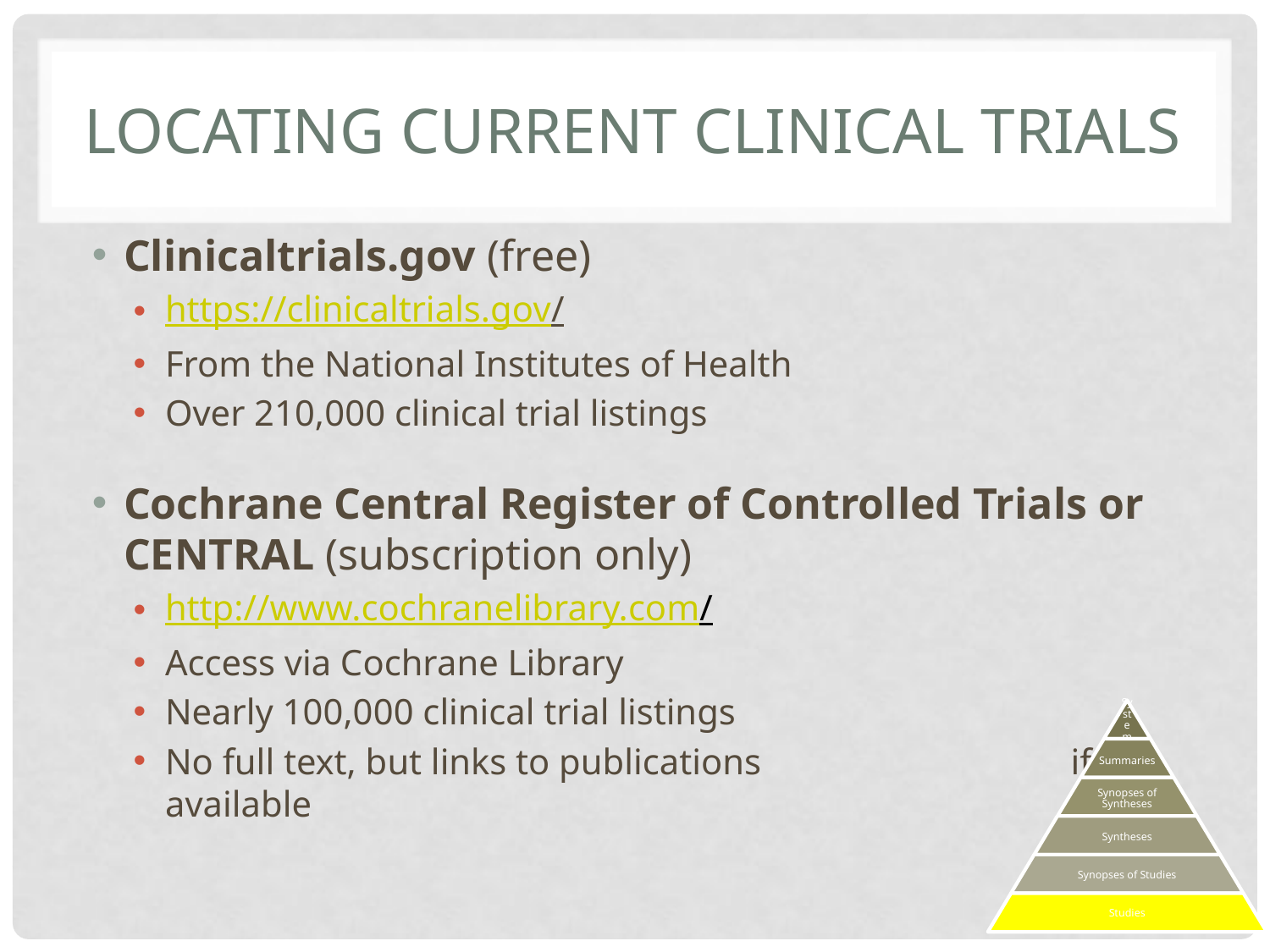

# Locating Current Clinical Trials
Clinicaltrials.gov (free)
https://clinicaltrials.gov/
From the National Institutes of Health
Over 210,000 clinical trial listings
Cochrane Central Register of Controlled Trials or CENTRAL (subscription only)
http://www.cochranelibrary.com/
Access via Cochrane Library
Nearly 100,000 clinical trial listings
No full text, but links to publications if available

## Slide 22
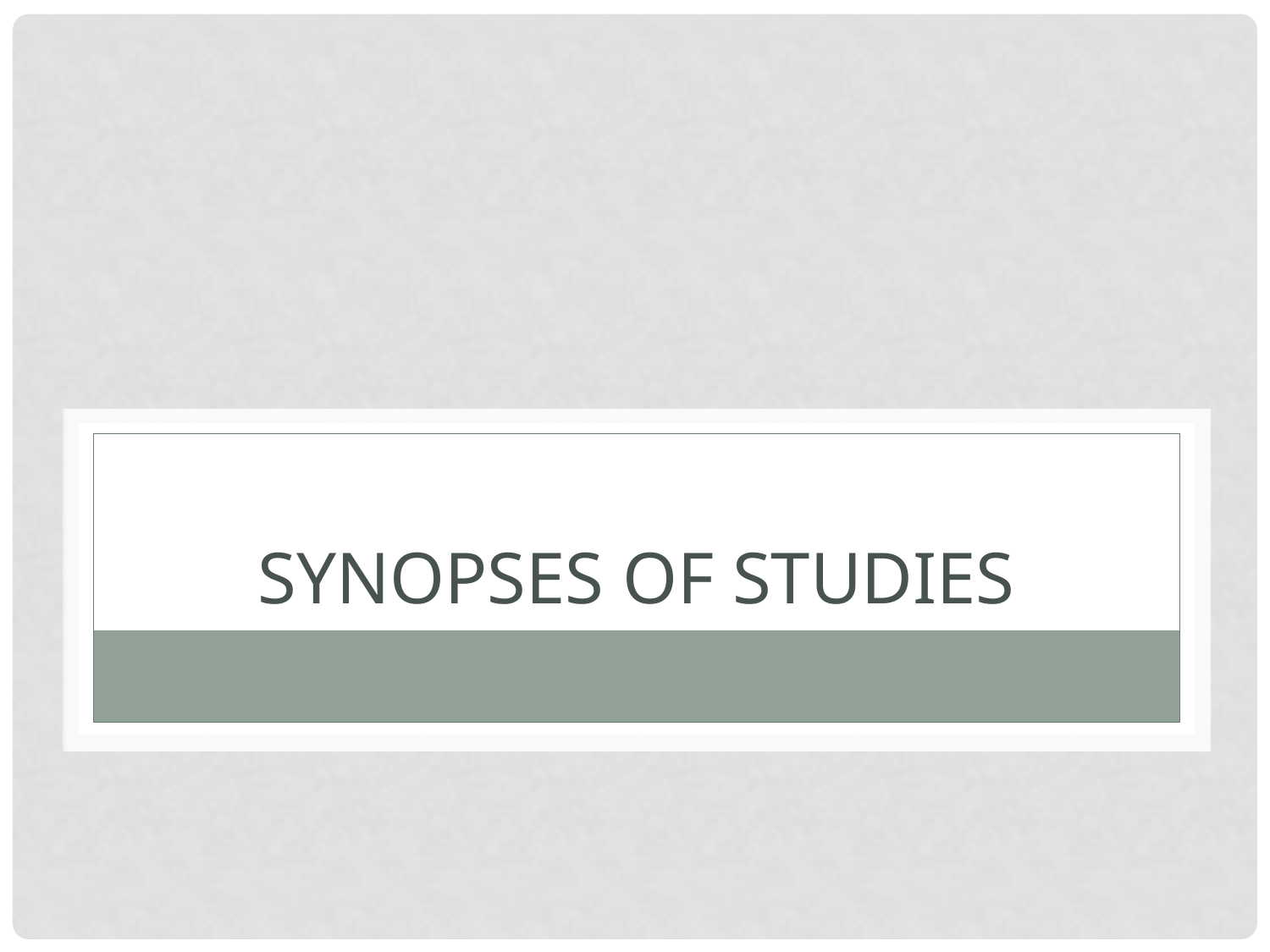

# SYNOPSES OF STUDIES

## Slide 23
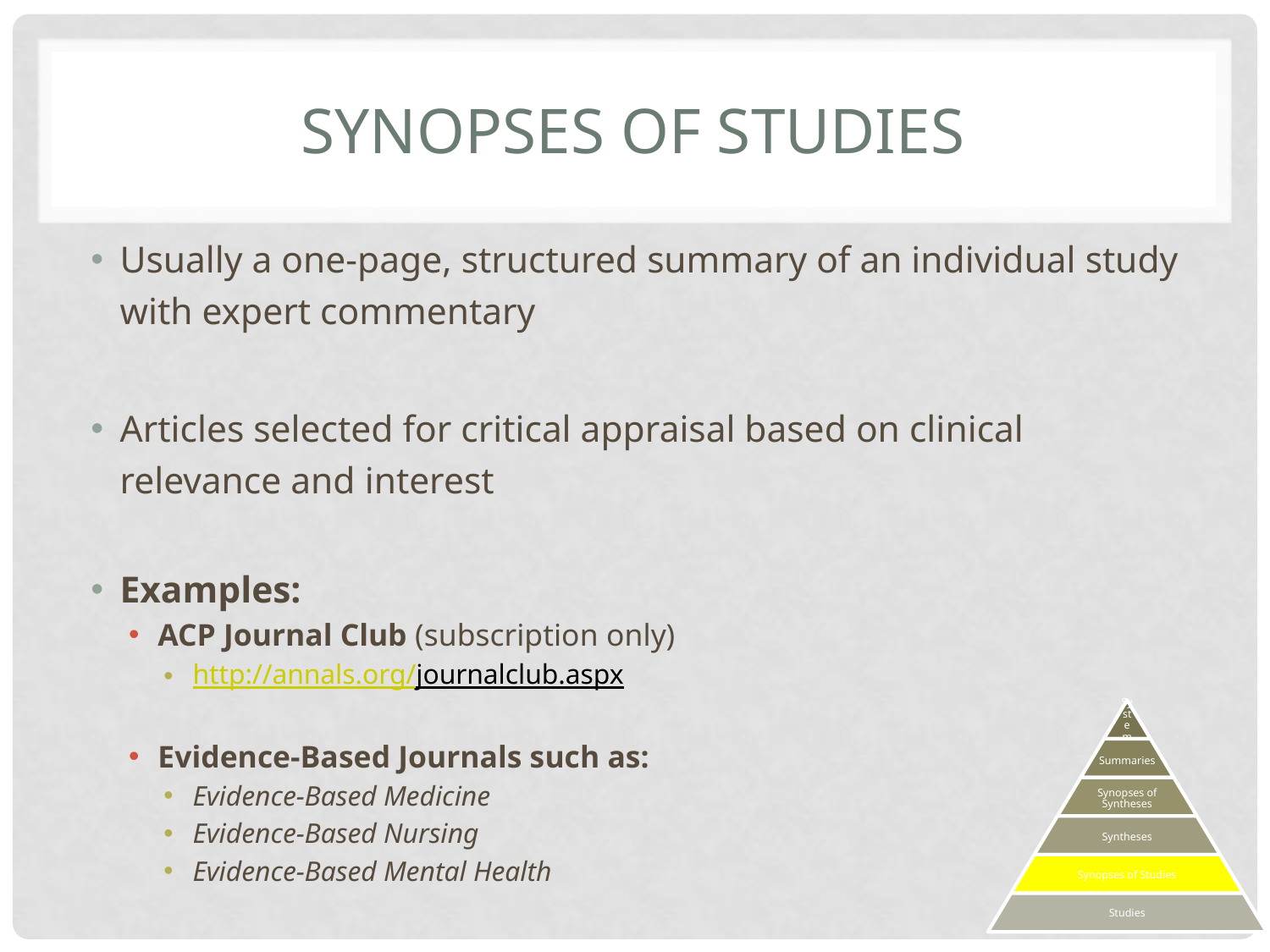

# Synopses of Studies
Usually a one-page, structured summary of an individual study with expert commentary
Articles selected for critical appraisal based on clinical relevance and interest
Examples:
ACP Journal Club (subscription only)
http://annals.org/journalclub.aspx
Evidence-Based Journals such as:
Evidence-Based Medicine
Evidence-Based Nursing
Evidence-Based Mental Health

## Slide 24
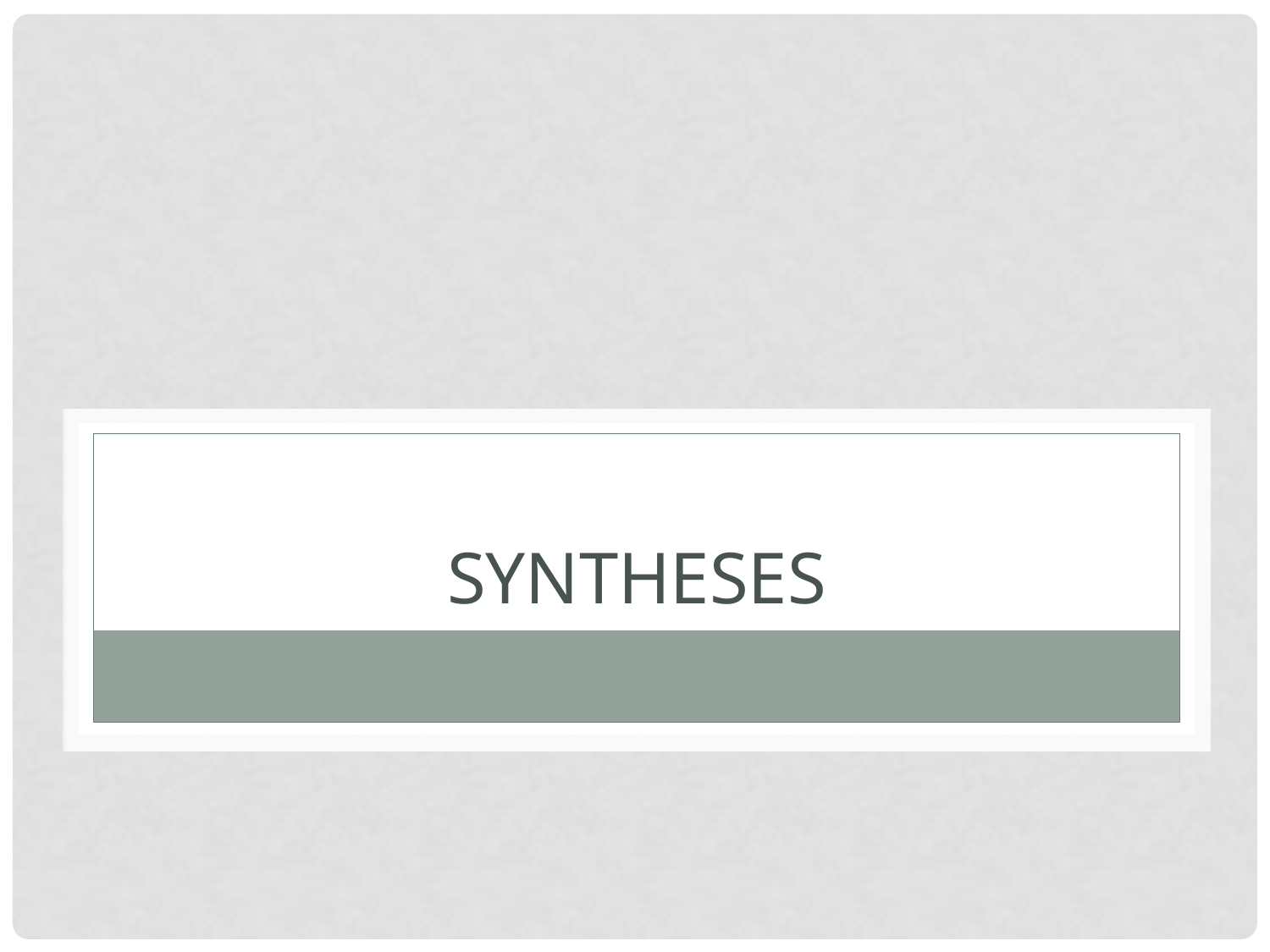

# SYNTHESES

## Slide 25
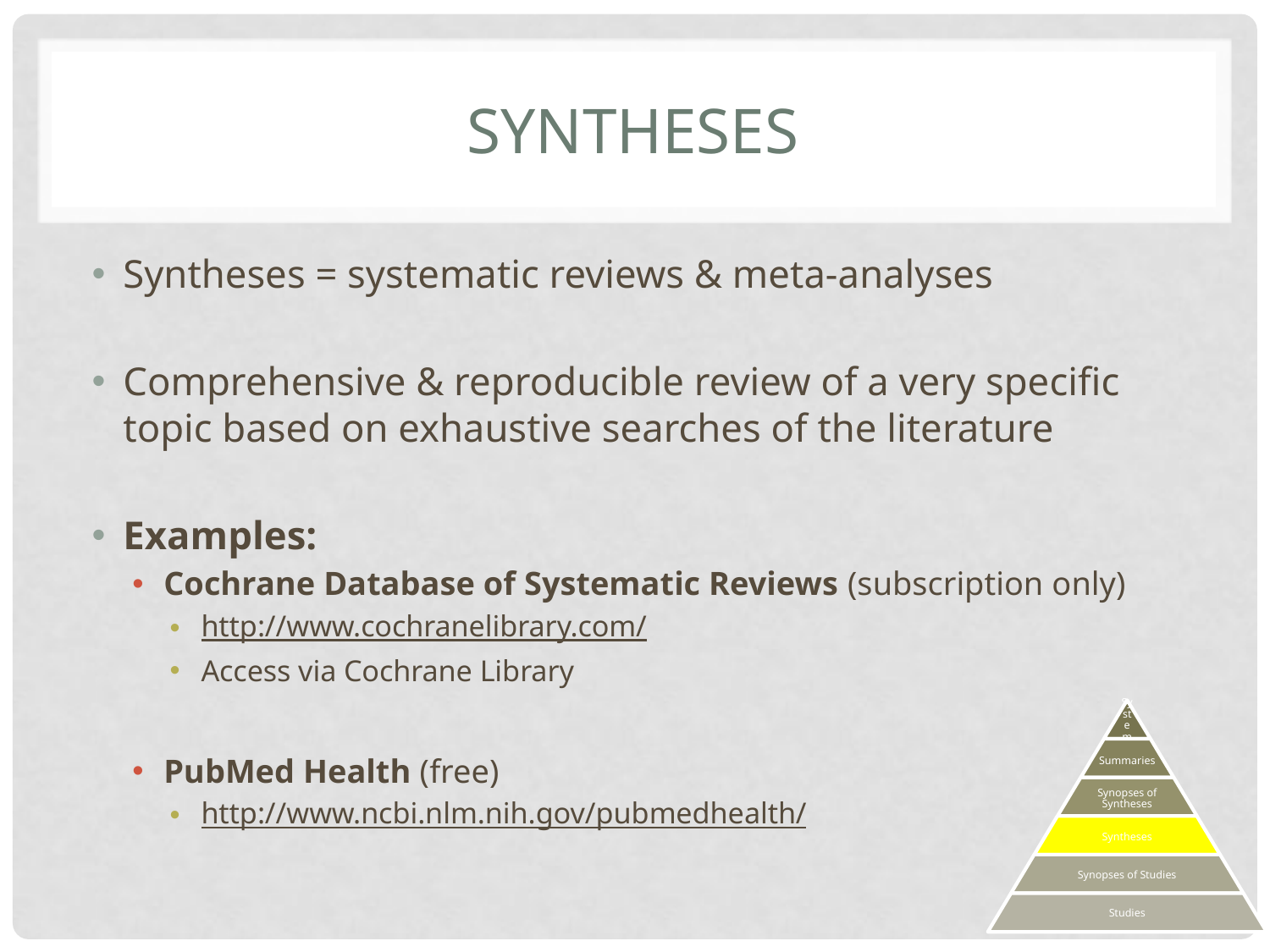

# Syntheses
Syntheses = systematic reviews & meta-analyses
Comprehensive & reproducible review of a very specific topic based on exhaustive searches of the literature
Examples:
Cochrane Database of Systematic Reviews (subscription only)
http://www.cochranelibrary.com/
Access via Cochrane Library
PubMed Health (free)
http://www.ncbi.nlm.nih.gov/pubmedhealth/

## Slide 26
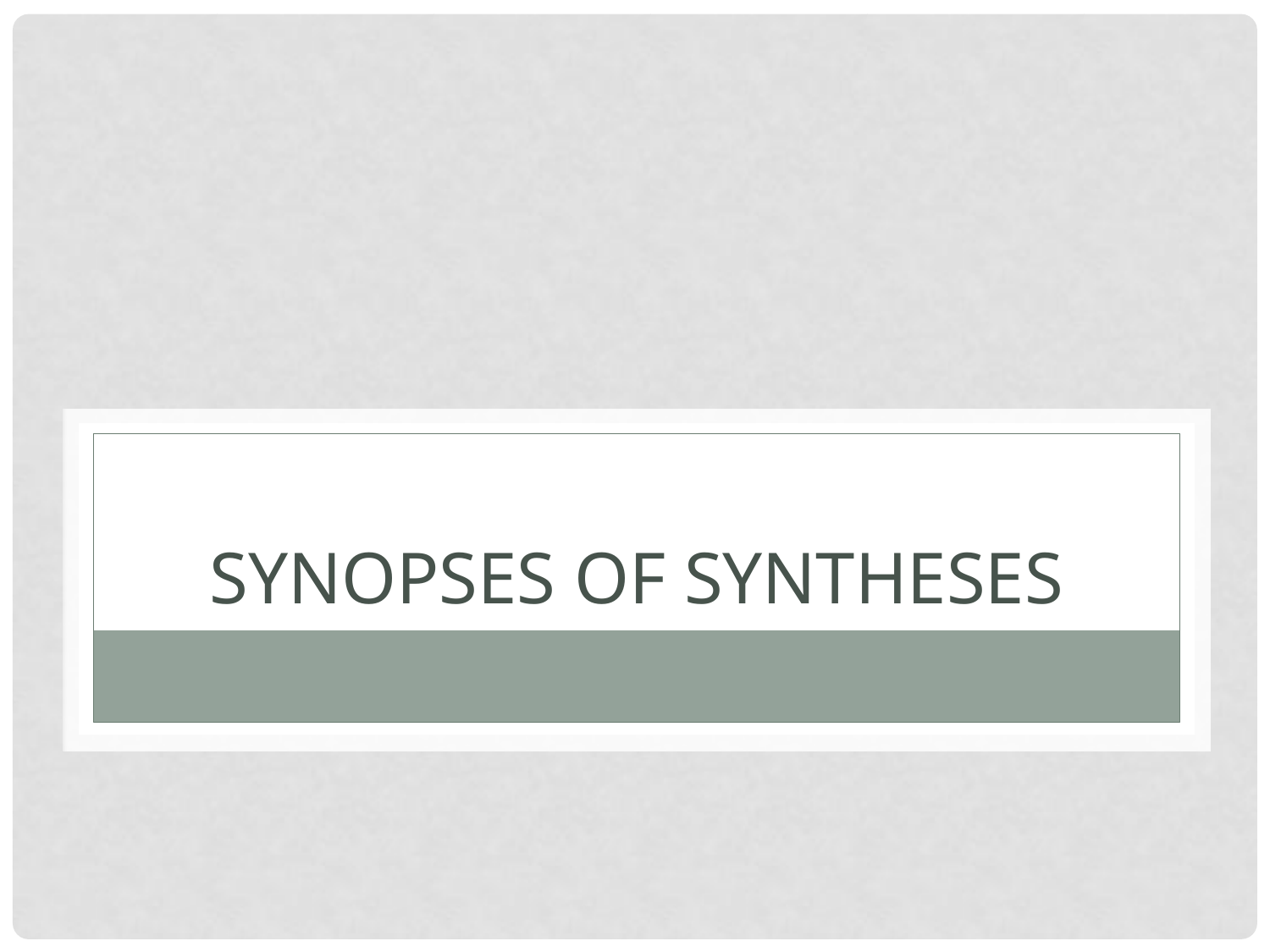

# SYNOPSES of syntheses

## Slide 27
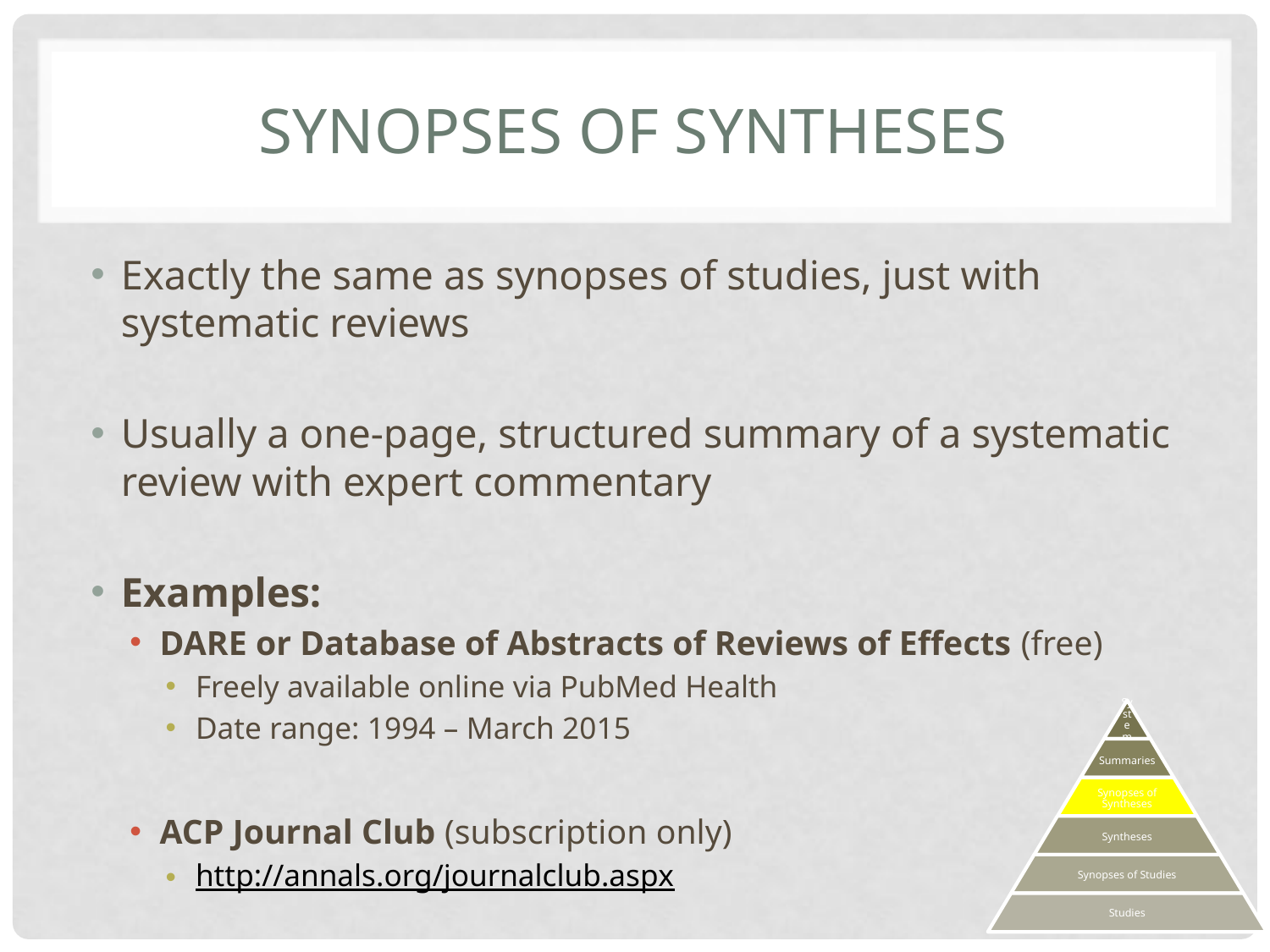

# Synopses of Syntheses
Exactly the same as synopses of studies, just with systematic reviews
Usually a one-page, structured summary of a systematic review with expert commentary
Examples:
DARE or Database of Abstracts of Reviews of Effects (free)
Freely available online via PubMed Health
Date range: 1994 – March 2015
ACP Journal Club (subscription only)
http://annals.org/journalclub.aspx

## Slide 28
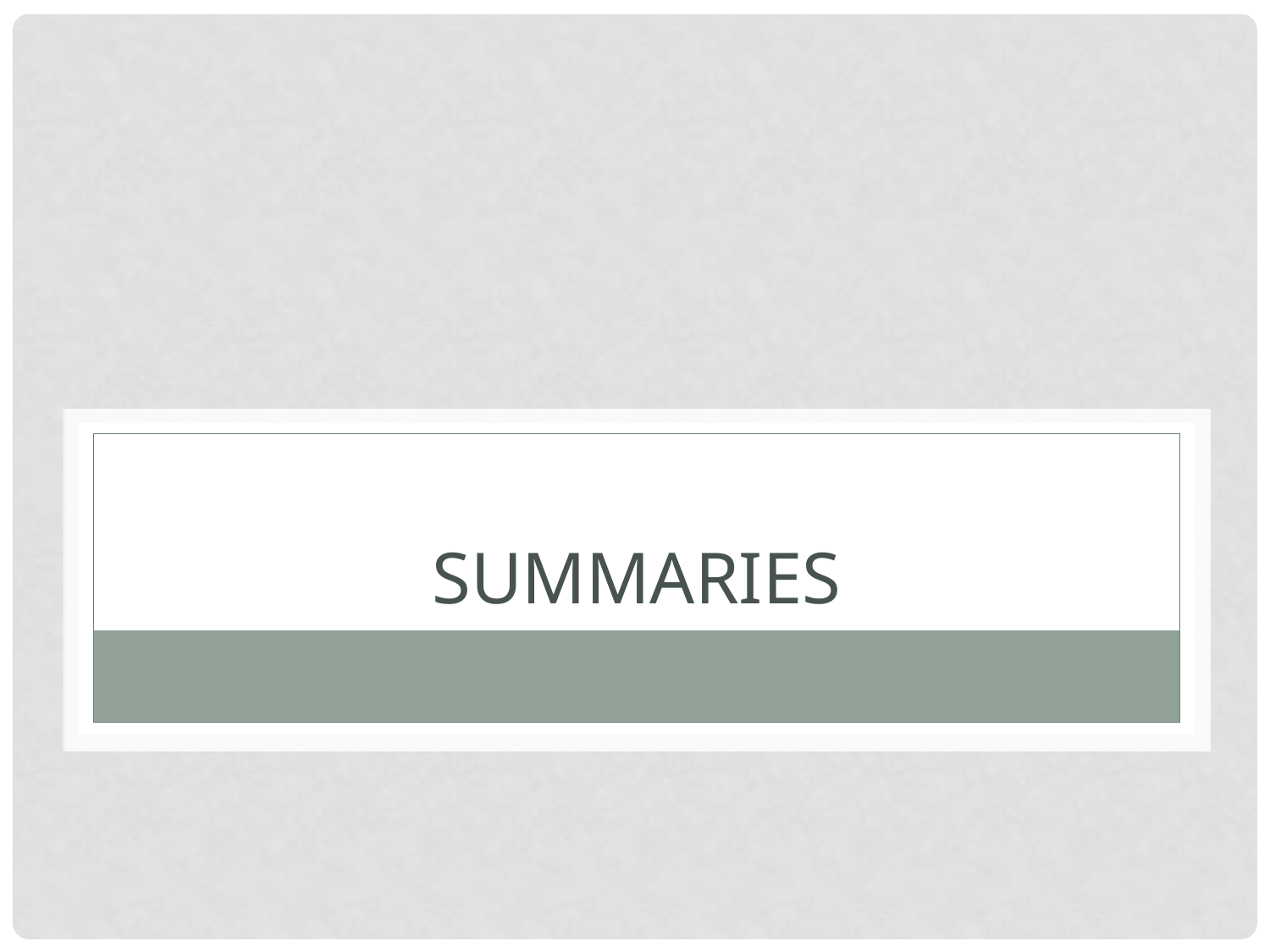

# Summaries

## Slide 29
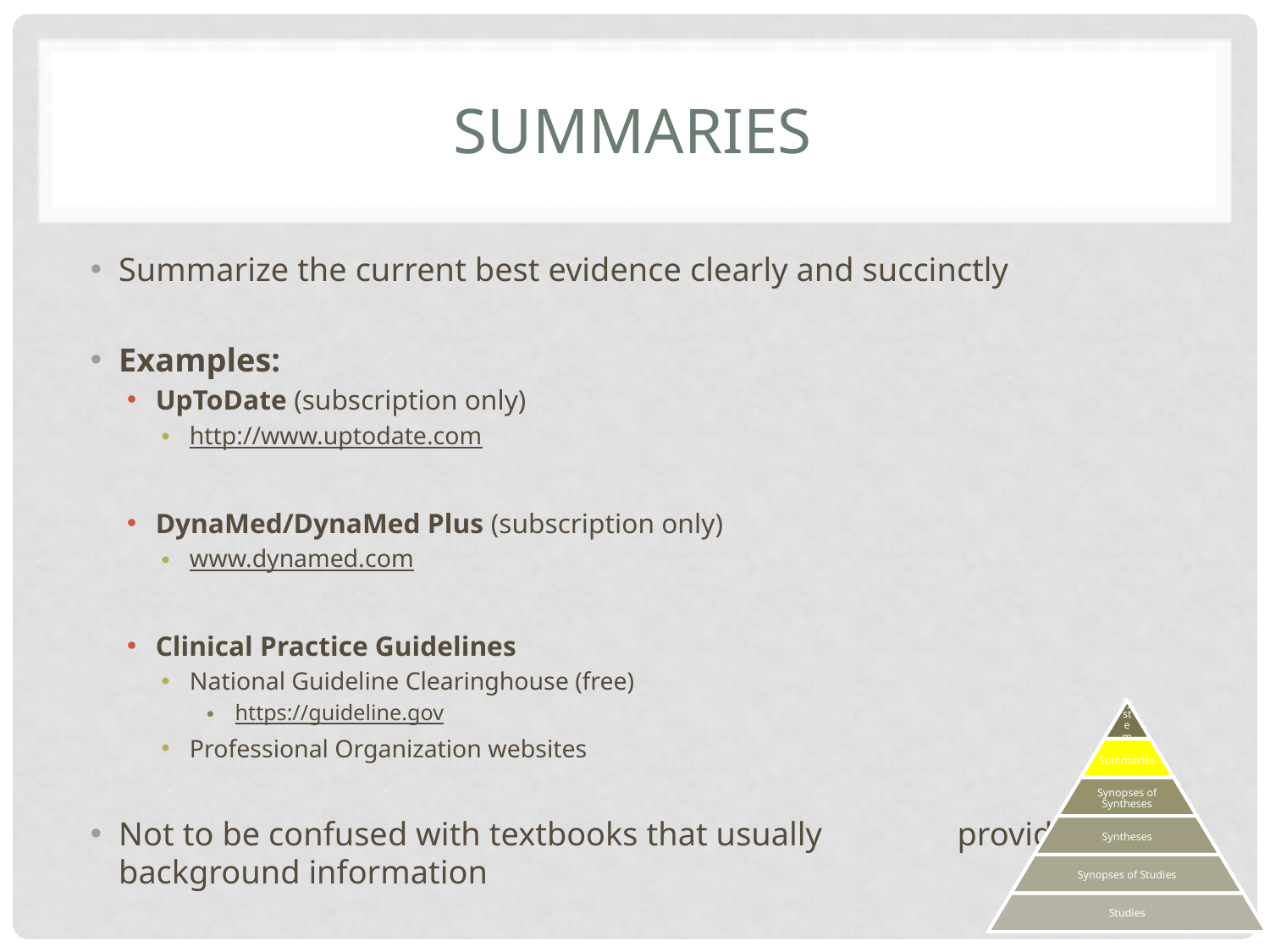

# Summaries
Summarize the current best evidence clearly and succinctly
Examples:
UpToDate (subscription only)
http://www.uptodate.com
DynaMed/DynaMed Plus (subscription only)
www.dynamed.com
Clinical Practice Guidelines
National Guideline Clearinghouse (free)
https://guideline.gov
Professional Organization websites
Not to be confused with textbooks that usually provide background information

## Slide 30
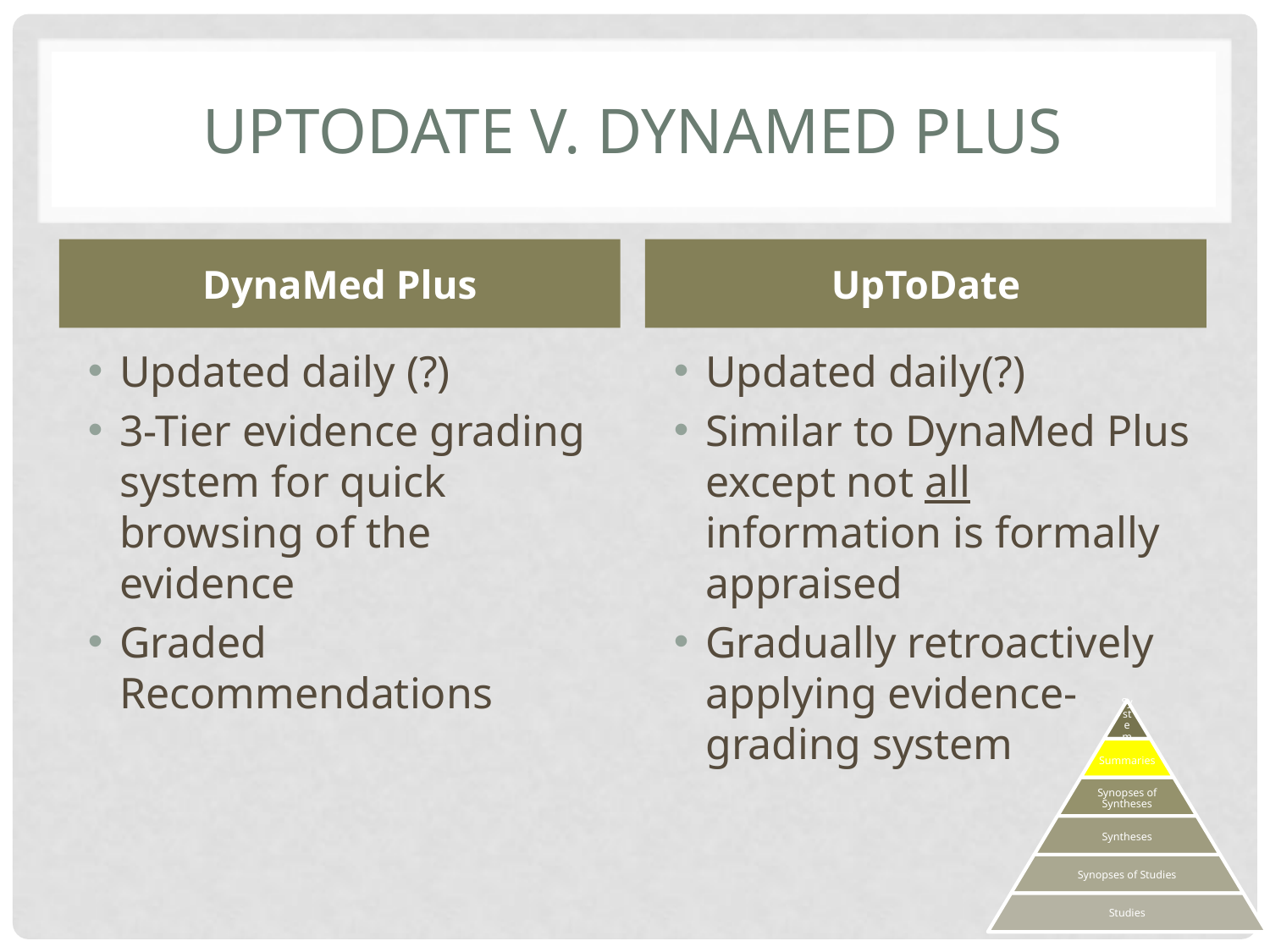

# UpToDate v. DynaMed Plus
DynaMed Plus
UpToDate
Updated daily (?)
3-Tier evidence grading system for quick browsing of the evidence
Graded Recommendations
Updated daily(?)
Similar to DynaMed Plus except not all information is formally appraised
Gradually retroactively applying evidence-grading system

## Slide 31
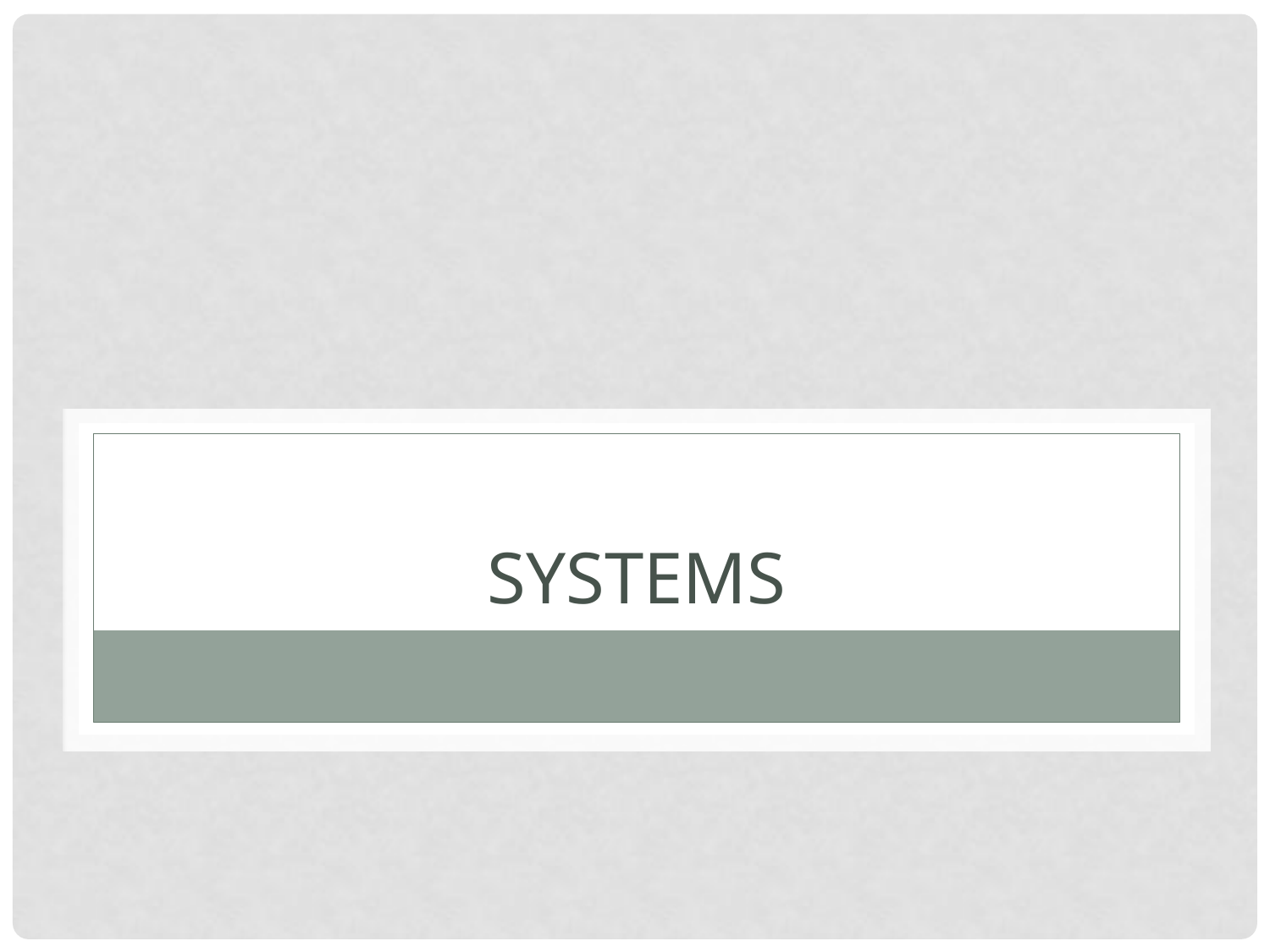

# systems

## Slide 32
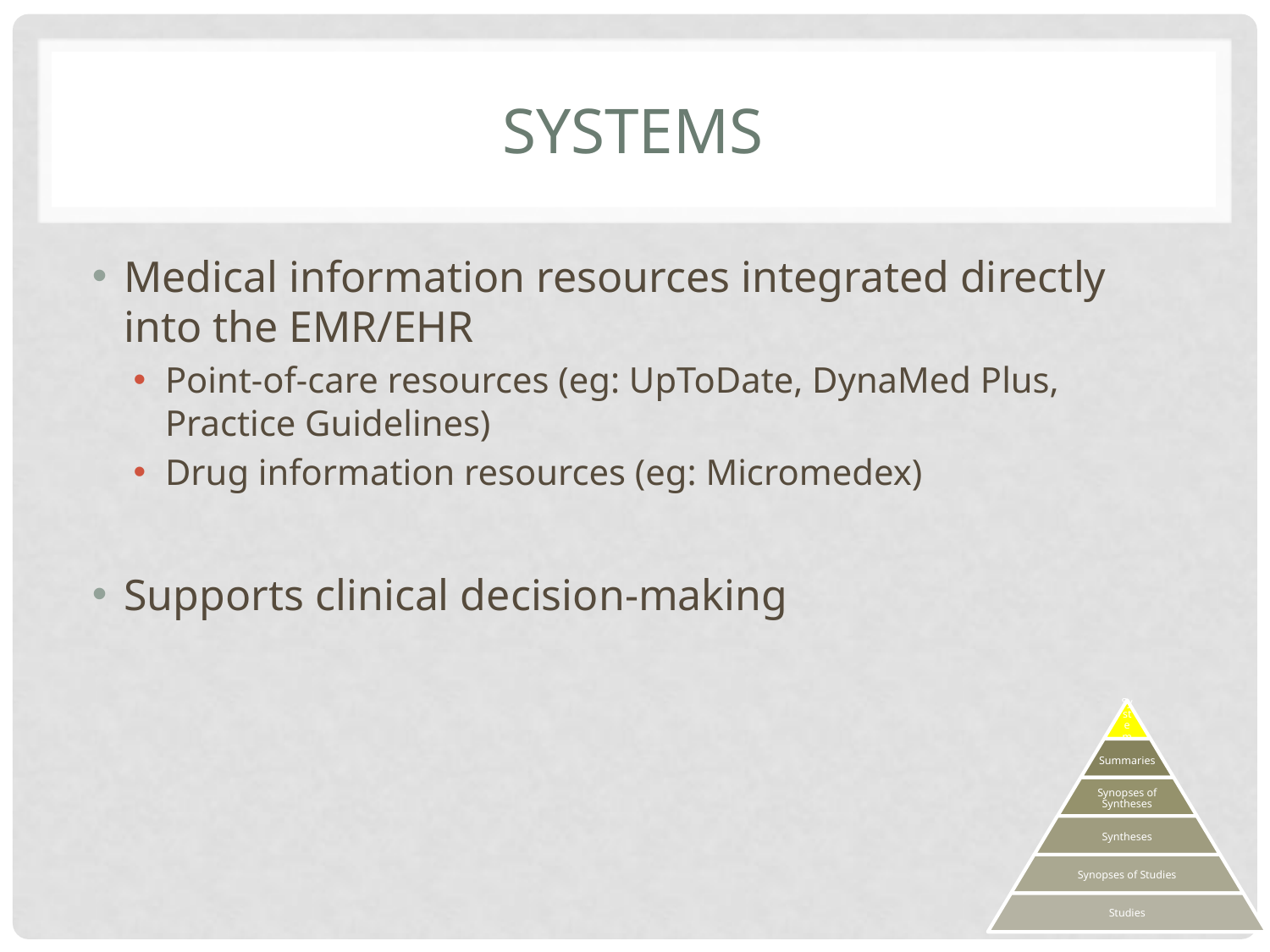

# Systems
Medical information resources integrated directly into the EMR/EHR
Point-of-care resources (eg: UpToDate, DynaMed Plus, Practice Guidelines)
Drug information resources (eg: Micromedex)
Supports clinical decision-making

## Slide 33
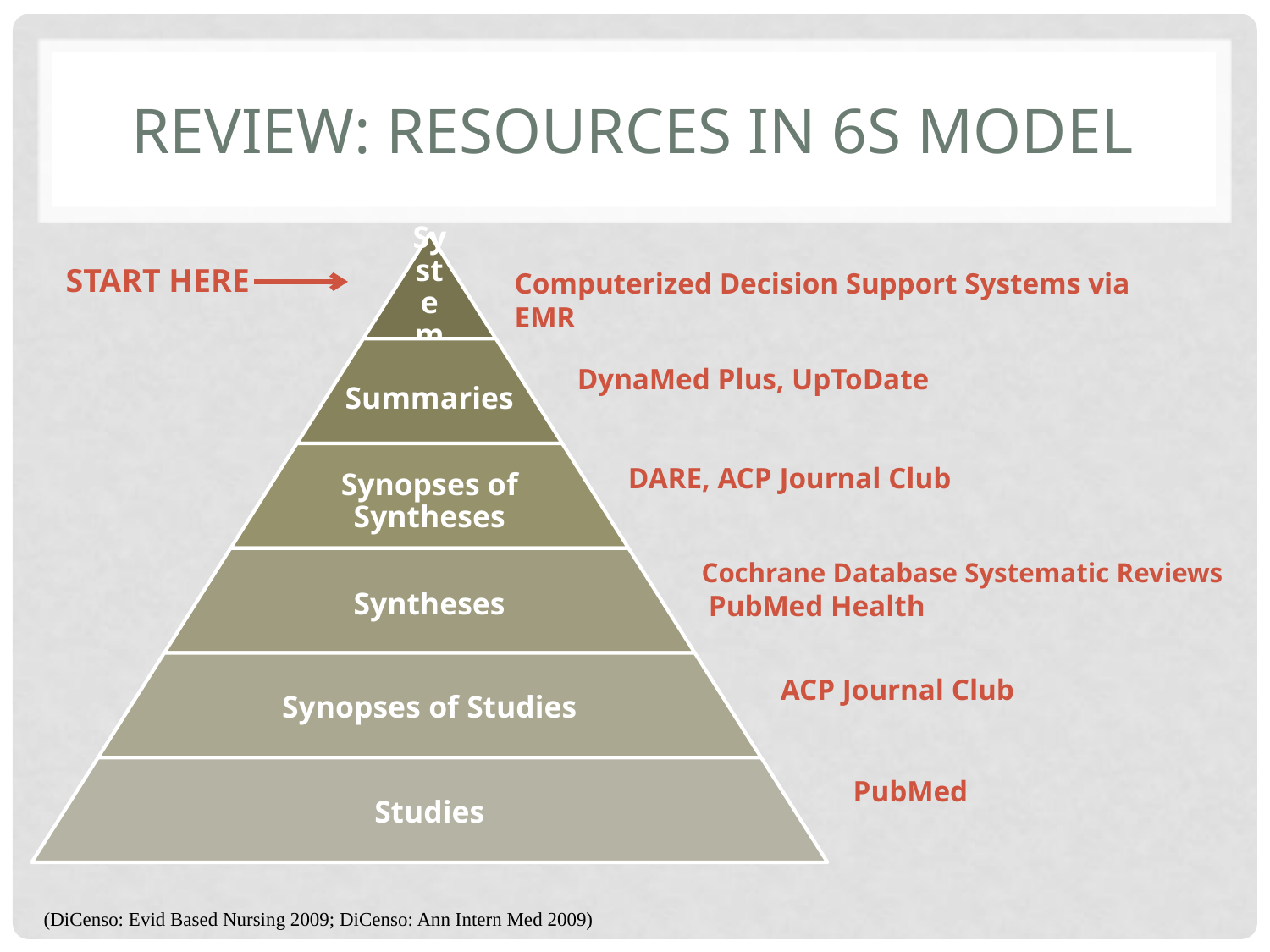

# REVIEW: Resources in 6S Model
START HERE
Computerized Decision Support Systems via EMR
DynaMed Plus, UpToDate
DARE, ACP Journal Club
Cochrane Database Systematic Reviews PubMed Health
 ACP Journal Club
PubMed
(DiCenso: Evid Based Nursing 2009; DiCenso: Ann Intern Med 2009)

## Slide 34
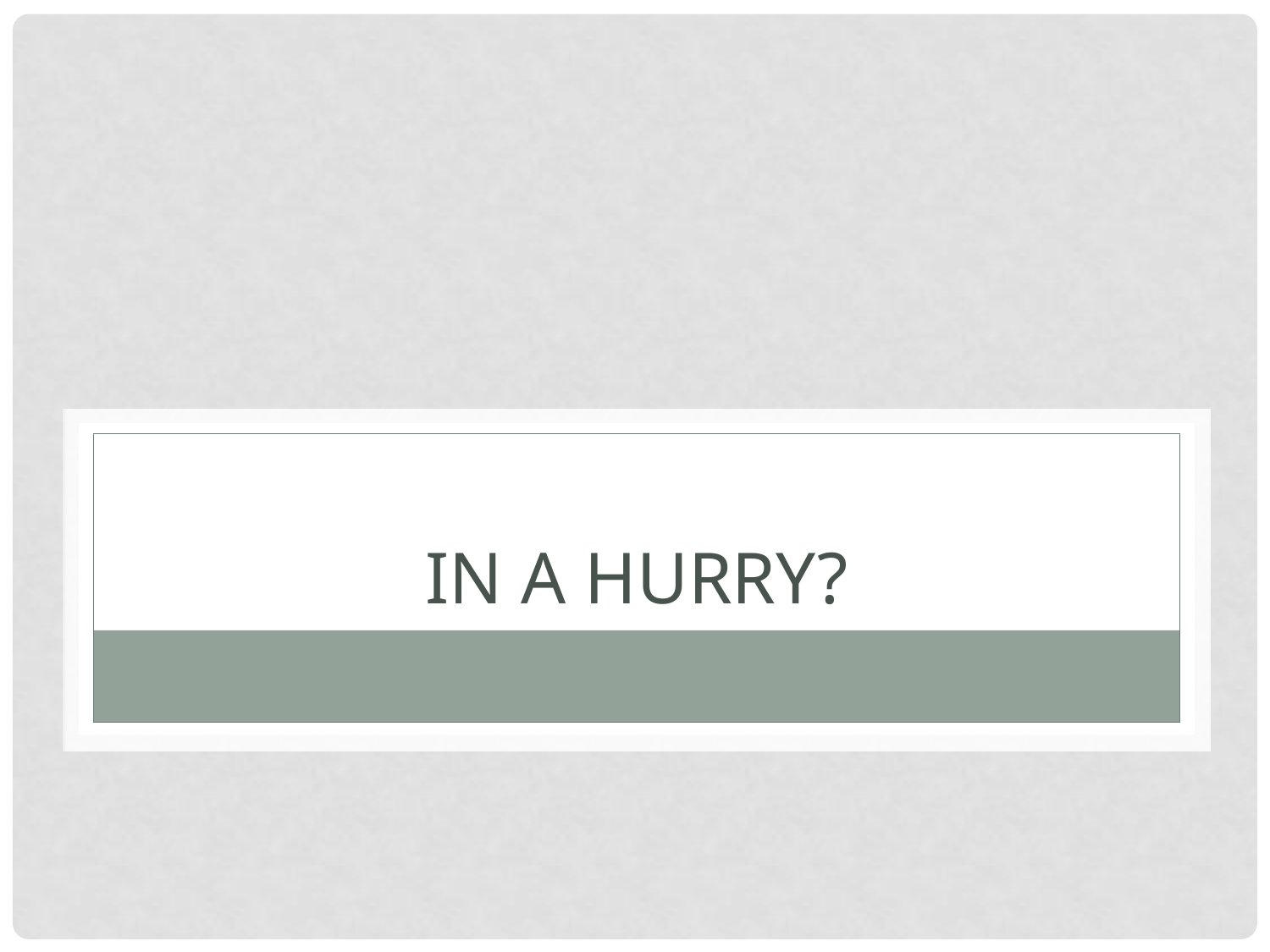

# In a hurry?

## Slide 35
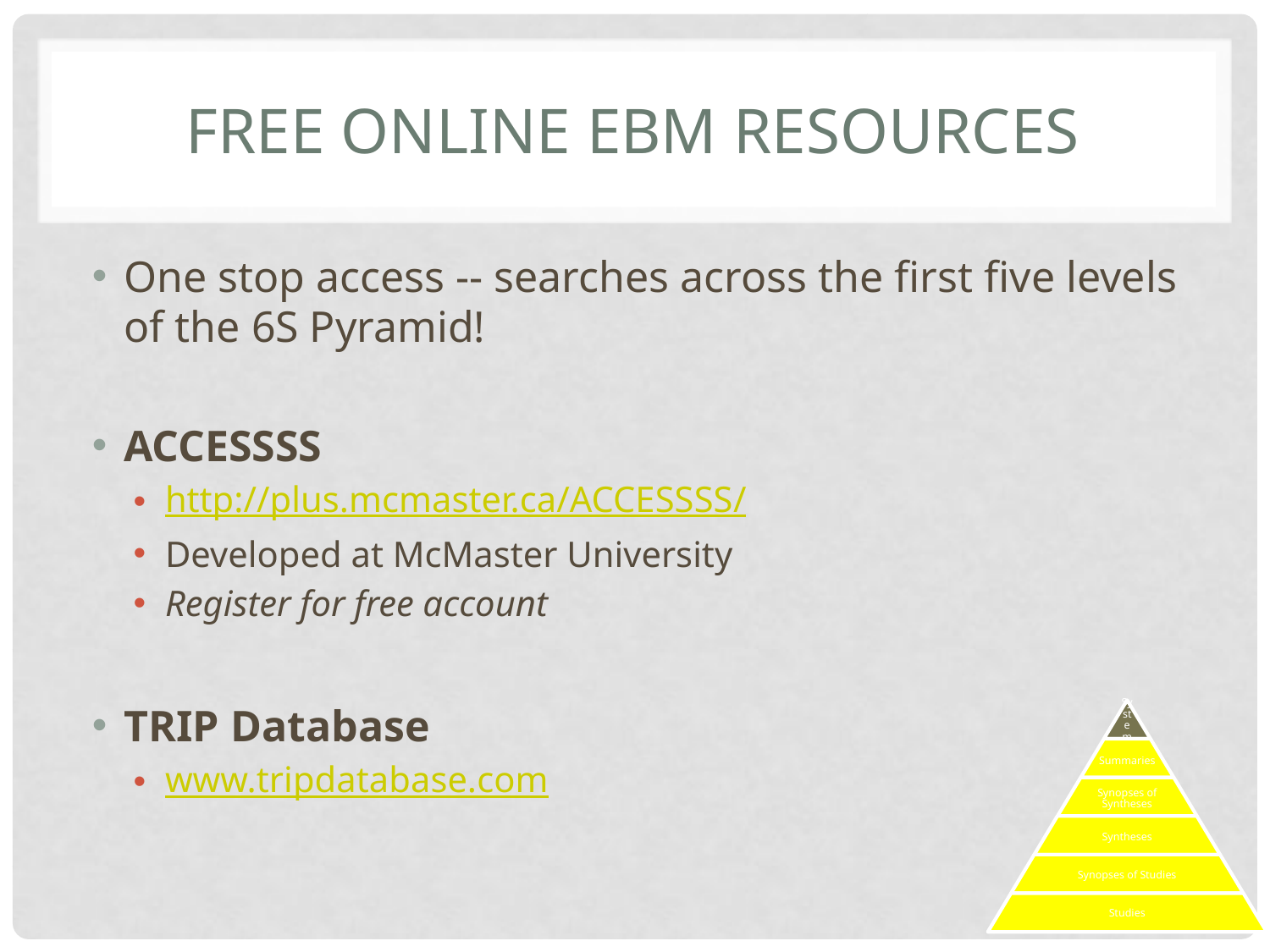

# Free Online EBM Resources
One stop access -- searches across the first five levels of the 6S Pyramid!
ACCESSSS
http://plus.mcmaster.ca/ACCESSSS/
Developed at McMaster University
Register for free account
TRIP Database
www.tripdatabase.com

## Slide 36
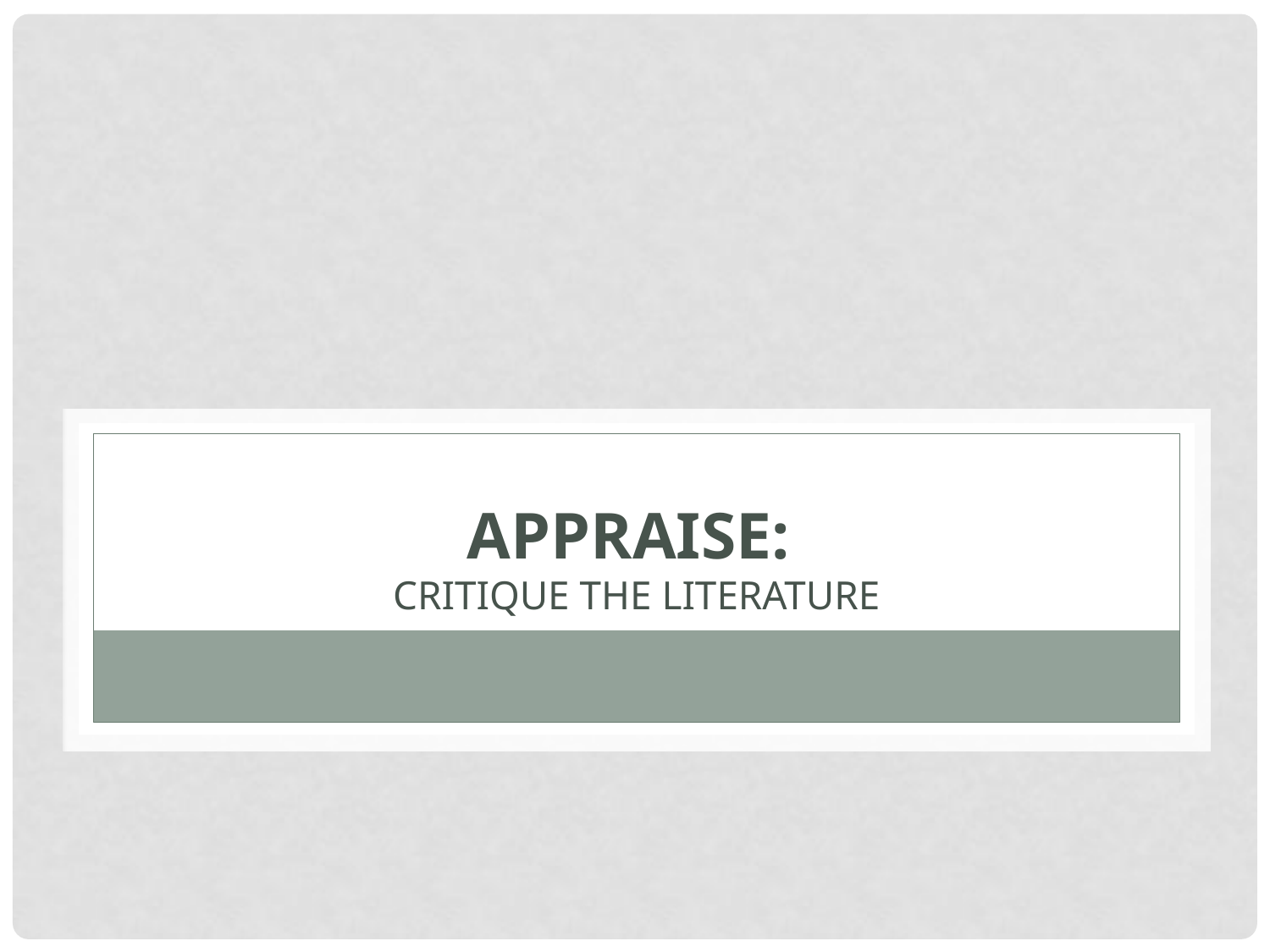

# APPRAISE: CRITIQUE the literature

## Slide 37
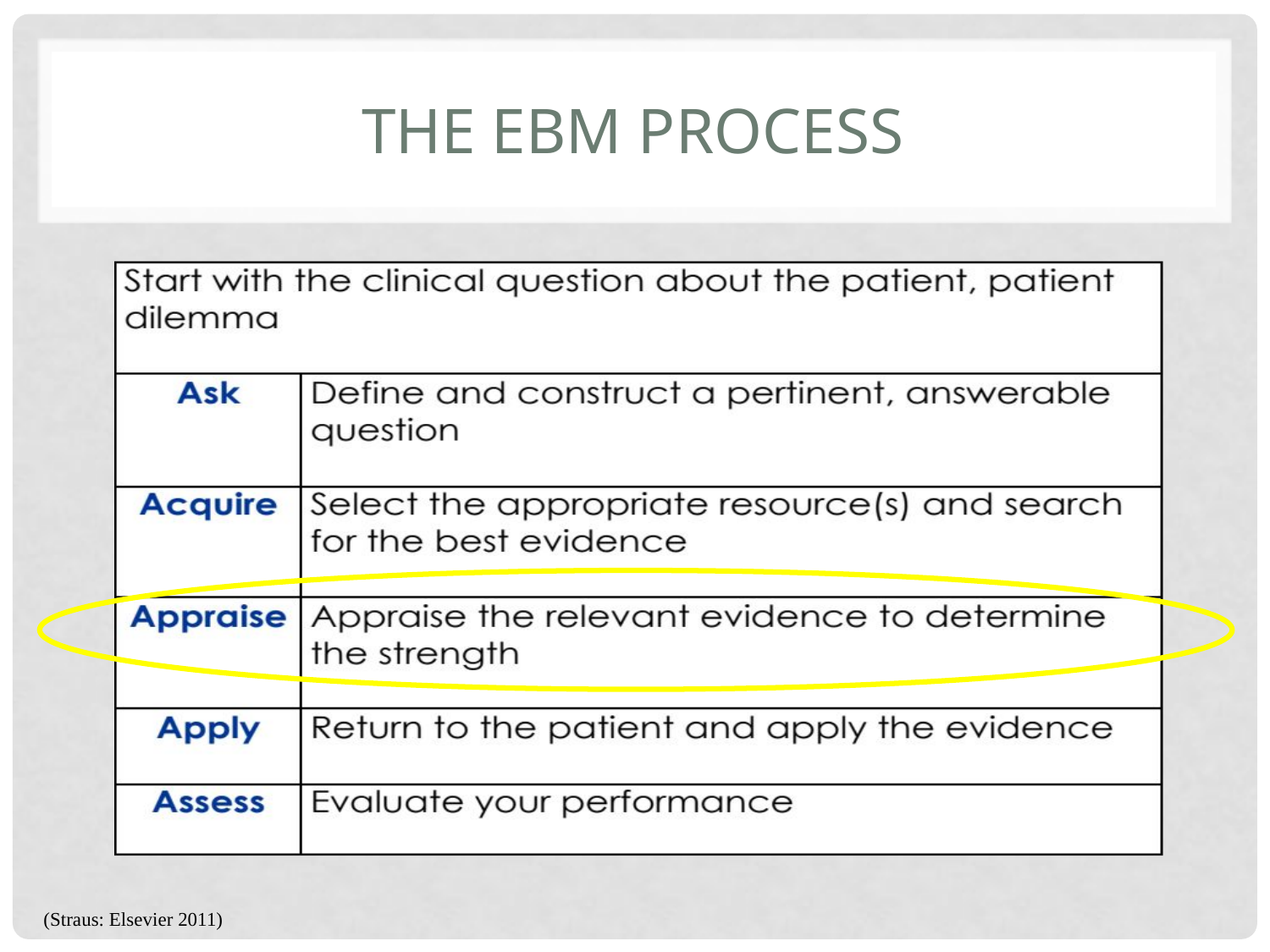

# The EBM Process
(Straus: Elsevier 2011)

## Slide 38
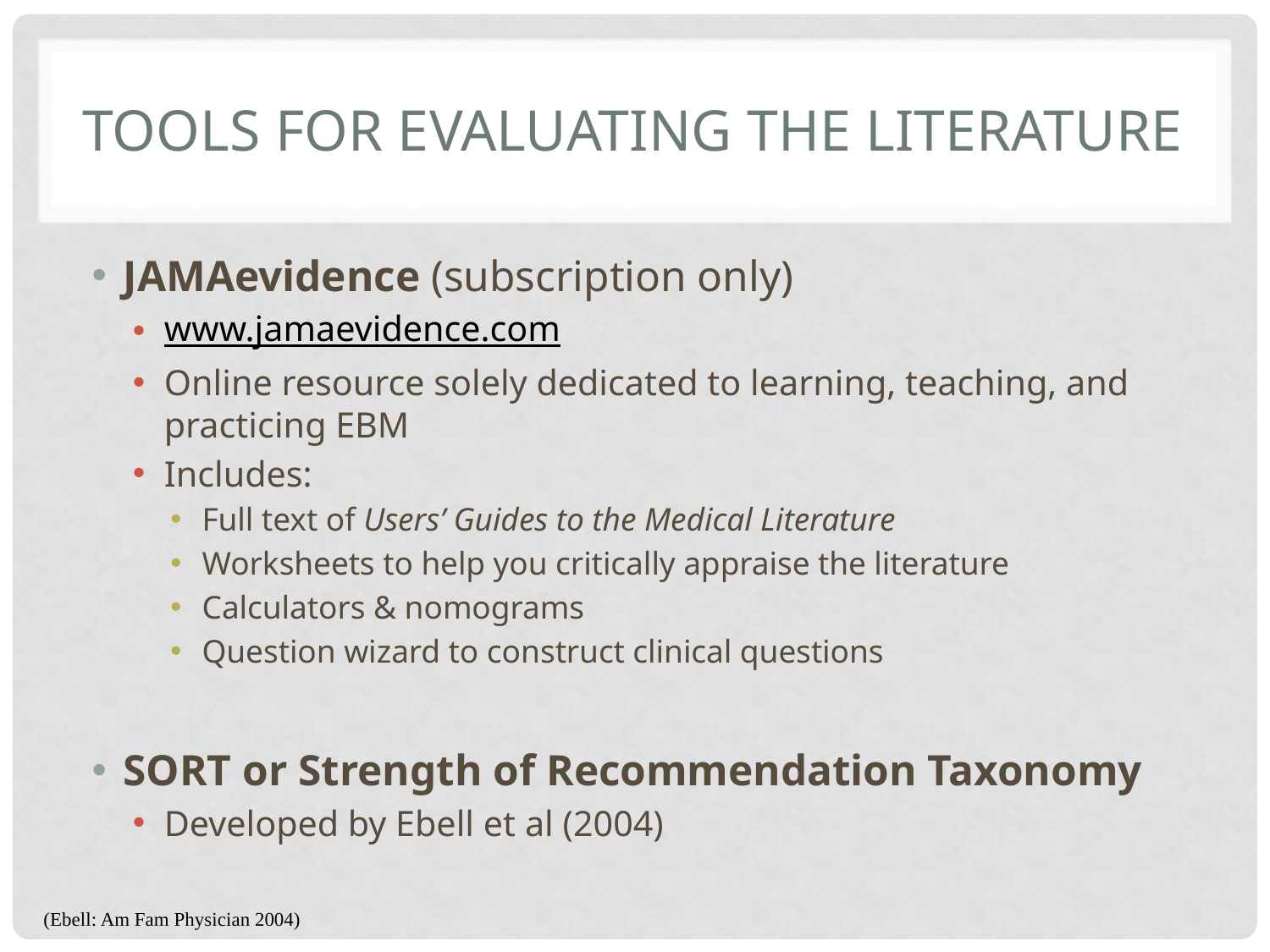

# Tools for Evaluating the Literature
JAMAevidence (subscription only)
www.jamaevidence.com
Online resource solely dedicated to learning, teaching, and practicing EBM
Includes:
Full text of Users’ Guides to the Medical Literature
Worksheets to help you critically appraise the literature
Calculators & nomograms
Question wizard to construct clinical questions
SORT or Strength of Recommendation Taxonomy
Developed by Ebell et al (2004)
(Ebell: Am Fam Physician 2004)

## Slide 39
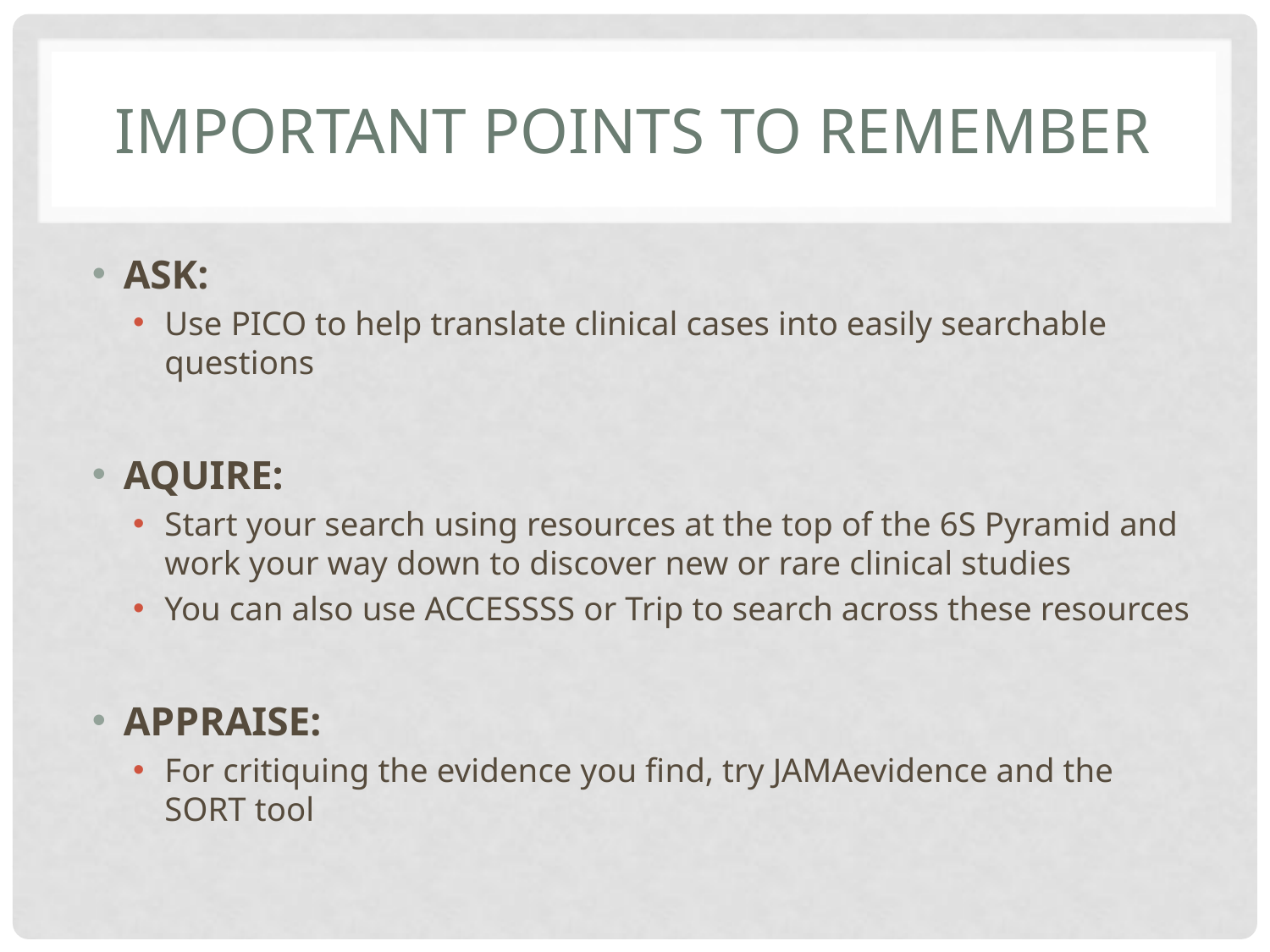

# Important Points to Remember
ASK:
Use PICO to help translate clinical cases into easily searchable questions
AQUIRE:
Start your search using resources at the top of the 6S Pyramid and work your way down to discover new or rare clinical studies
You can also use ACCESSSS or Trip to search across these resources
APPRAISE:
For critiquing the evidence you find, try JAMAevidence and the SORT tool

## Slide 40
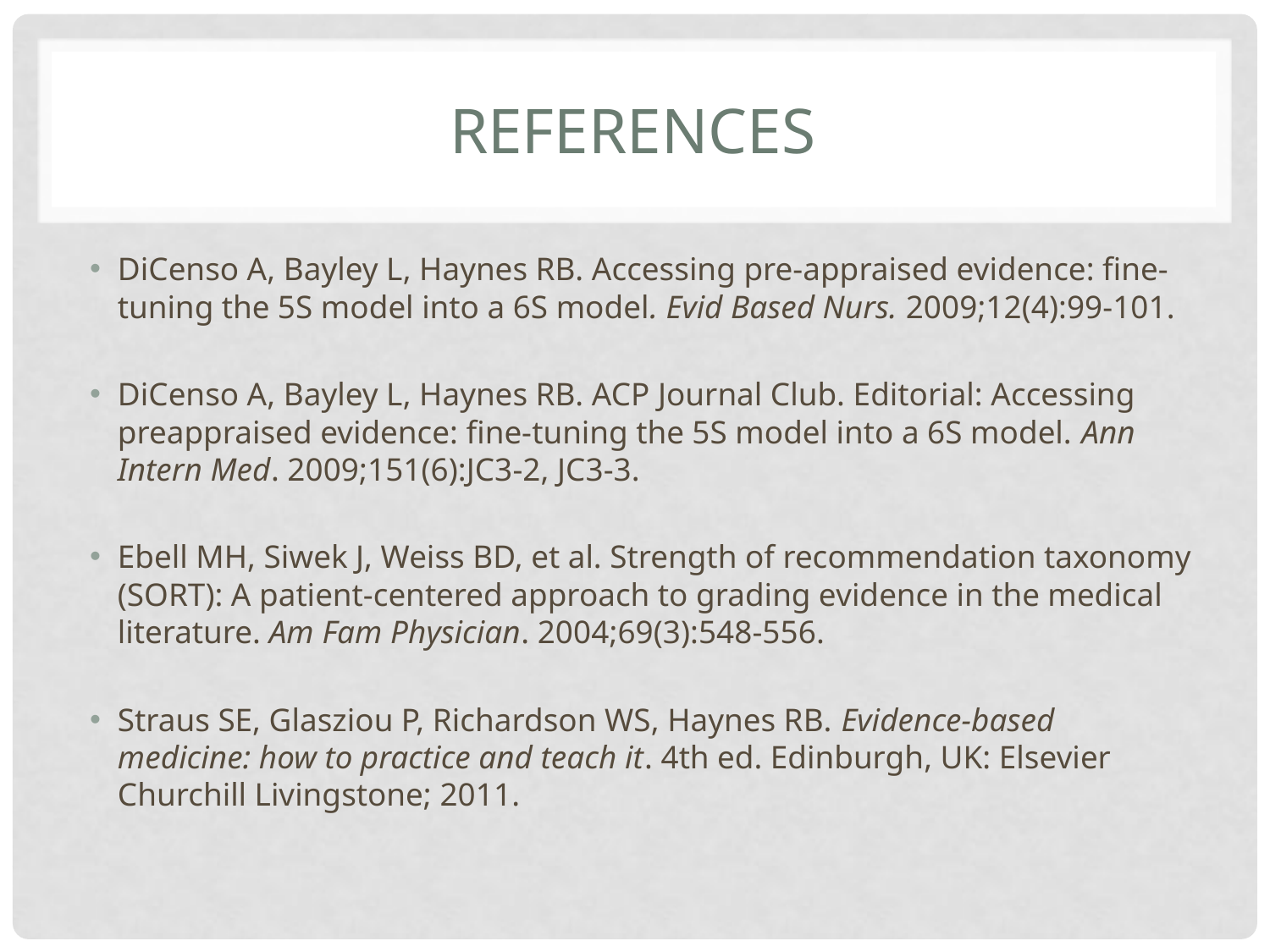

# References
DiCenso A, Bayley L, Haynes RB. Accessing pre-appraised evidence: fine-tuning the 5S model into a 6S model. Evid Based Nurs. 2009;12(4):99-101.
DiCenso A, Bayley L, Haynes RB. ACP Journal Club. Editorial: Accessing preappraised evidence: fine-tuning the 5S model into a 6S model. Ann Intern Med. 2009;151(6):JC3-2, JC3-3.
Ebell MH, Siwek J, Weiss BD, et al. Strength of recommendation taxonomy (SORT): A patient-centered approach to grading evidence in the medical literature. Am Fam Physician. 2004;69(3):548-556.
Straus SE, Glasziou P, Richardson WS, Haynes RB. Evidence-based medicine: how to practice and teach it. 4th ed. Edinburgh, UK: Elsevier Churchill Livingstone; 2011.
