# Supplementary material for: An Integrated, Case-Based Approach to Teaching Medical Students How to Locate the Best Available Evidence for Clinical Care
Source: MedEdPORTAL. 2017 Jan 19;13:10531. doi: 10.15766/mep_2374-8265.10531 (PMC6342155; doi:10.15766/mep_2374-8265.10531)
Supplement: Supplementary file 1 — A. Locating the Best Available Evidence Lecture-Text.docx B. Locating the Best Available Evidence Lecture.pptx C. Lab Facilitator Guide.docx D. Lab Review Questions.pptx E. Lab Worksheet Case 1-Blank.docx F. Lab Worksheet Case 1-Answer Key.docx G. Lab Worksheet Case 2-Blank.docx H. Lab Worksheet Case 2-Answer Key.docx I. Case Presentation Evaluation Rubric.docx [file mep-13-10531-s001.zip › C. Lab Facilitator Guide.docx]

**Lab Facilitator Guide**

**Locating the Best Available Evidence Lab**

| **TIME** | **ACTIVITY** |
| --- | --- |
| **Lab - Hour 1** | |
| **5 minutes** | **OVERVIEW OF LAB**   1. Ask all students to get into their assigned teams 2. Ask all students to download and open lab worksheet (Appendix D) from the course management system 3. Read lab introduction    - 1. In this lab, you will simulate conducting the first two steps of the evidence-based medicine process by 1) formulating a clear clinical question and 2) gathering the evidence from various evidence-based resources for one patient case and your team case presentation project.      2. This lab will prepare you not only for the coming sessions, especially, but also for your clerkships, residencies, and careers where you will be expected to find, evaluate, and present evidence for patient cases and journal clubs. 4. Ask students if they have any questions about the session |
| **5 minutes** | **LARGE GROUP VERBAL QUIZ**   1. Appendix C includes 3 quiz questions, which can either be displayed on PowerPoint slides or transferred to a Word document and passed out. As a large group, go through each review question. These questions review the major concepts the students should have learned from reviewing the online tutorial or videos OR attending lecture and act as a quick refresher before starting the lab. |
| **25 minutes** | **TEAMS WORK THROUGH CASE 1**   1. Encourage students to ask the facilitator questions as they arise regarding particular resources. Just as in other labs, this provides an opportunity for informal teaching moments with individual students or groups. |
| **15 minutes** | **LARGE GROUP REVIEW OF CASE 1**   1. Ask students if they have any lingering questions and/or share any trends you were seeing in student questions during the work time 2. Go through Case 1 starting with PICO, calling on individual teams to present their answers. If you have time, you can have students come up and demonstrate how they searched to find the evidence. |
| **10 minutes** | **BREAK** |
| **Lab - Hour 2** | |
| **45 minutes** | **EBM CASE PRESENTATION WORK TIME OR CASE 2**   1. Depending on how this session is integrated with other EBM content, the second half of the lab could either:    1. Give students time as a team to work on their EBM Case Presentation, which they will present as an oral presentation at the end of the course. Teams should discuss a clinical case they would like to tackle and work through the first two steps of the EBM process, ASK and ACQUIRE.   **OR**   - 1. Teams work through Case 2 provided in Appendix F and follow the same time structure as above. |
| **5 minutes** | **CONCLUSION**   1. Ask students if there are any lingering questions 2. Instruct students to take the last 5 minutes to complete/review their team document and submit ONE assignment per group to the course management system. Students should be able to finish and submit in class, but are given until 11:55pm that night to submit. |
