# Supplementary material for: An Integrated, Case-Based Approach to Teaching Medical Students How to Locate the Best Available Evidence for Clinical Care
Source: MedEdPORTAL. 2017 Jan 19;13:10531. doi: 10.15766/mep_2374-8265.10531 (PMC6342155; doi:10.15766/mep_2374-8265.10531)
Supplement: Supplementary file 1 — A. Locating the Best Available Evidence Lecture-Text.docx B. Locating the Best Available Evidence Lecture.pptx C. Lab Facilitator Guide.docx D. Lab Review Questions.pptx E. Lab Worksheet Case 1-Blank.docx F. Lab Worksheet Case 1-Answer Key.docx G. Lab Worksheet Case 2-Blank.docx H. Lab Worksheet Case 2-Answer Key.docx I. Case Presentation Evaluation Rubric.docx [file mep-13-10531-s001.zip › D. Lab Review Questions.pptx]

## Slide 1
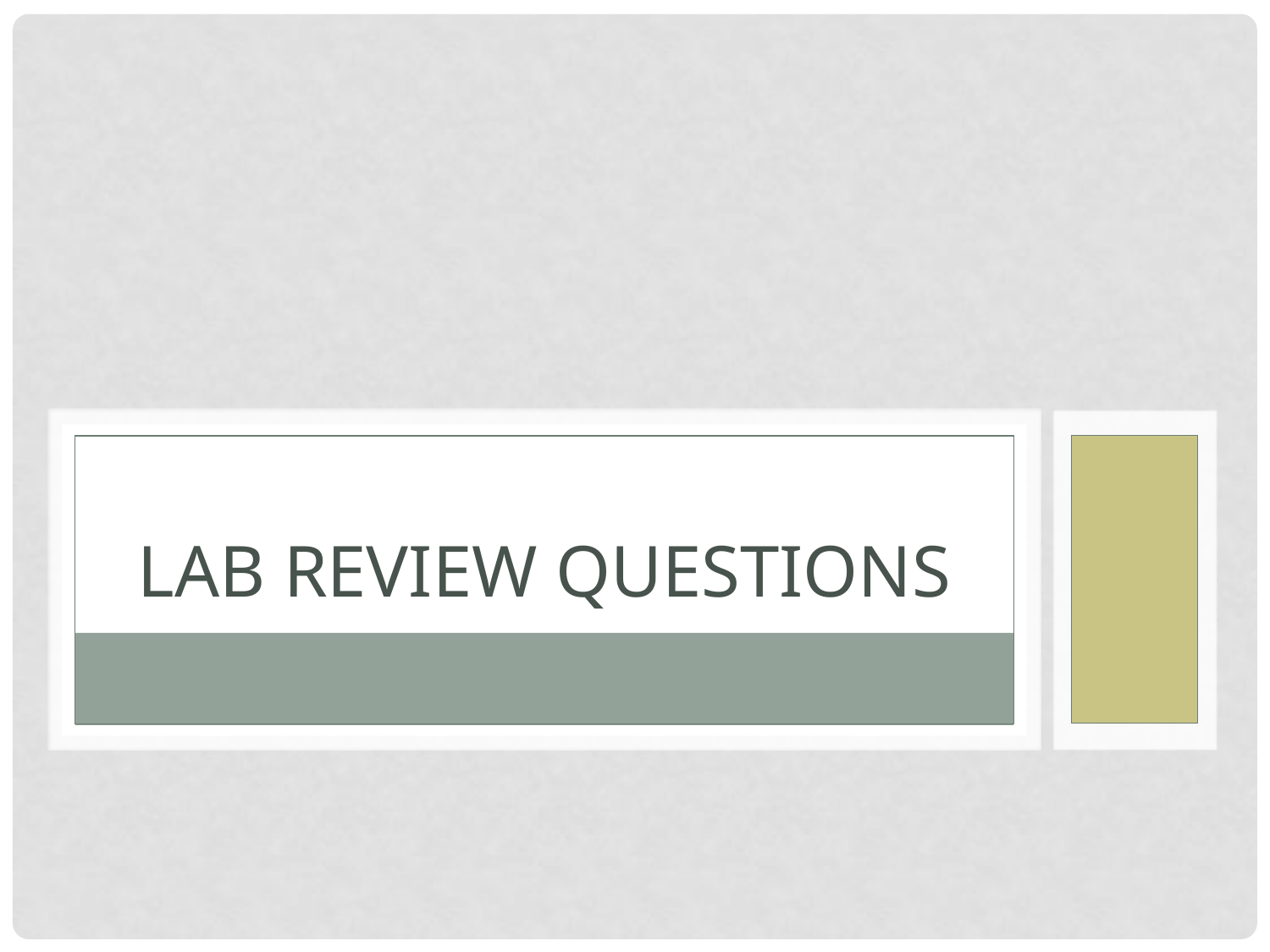

# Lab Review Questions

## Slide 2
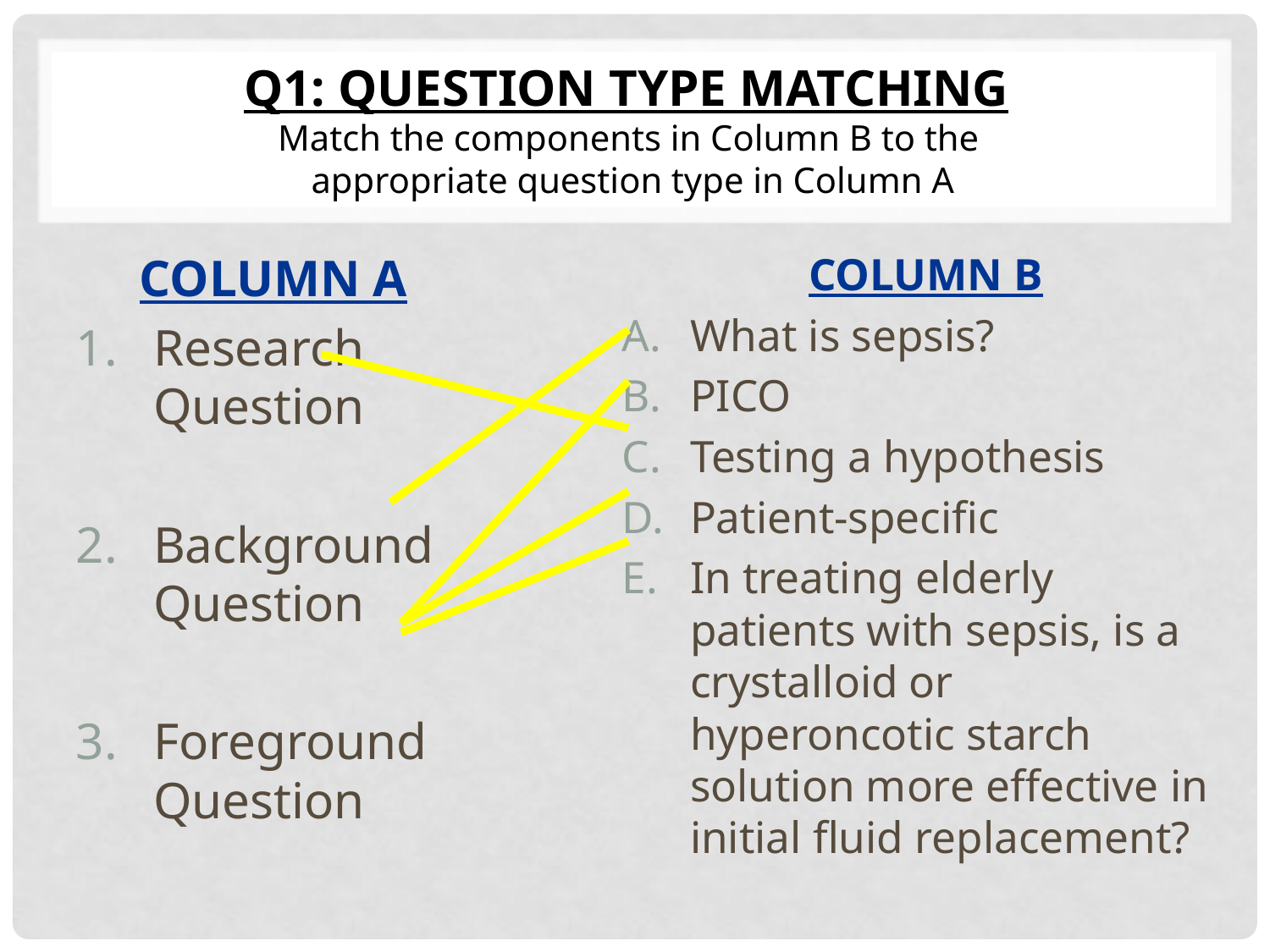

# Q1: Question Type Matching Match the components in Column B to the appropriate question type in Column A
COLUMN A
Research Question
Background Question
Foreground Question
COLUMN B
What is sepsis?
PICO
Testing a hypothesis
Patient-specific
In treating elderly patients with sepsis, is a crystalloid or hyperoncotic starch solution more effective in initial fluid replacement?

## Slide 3
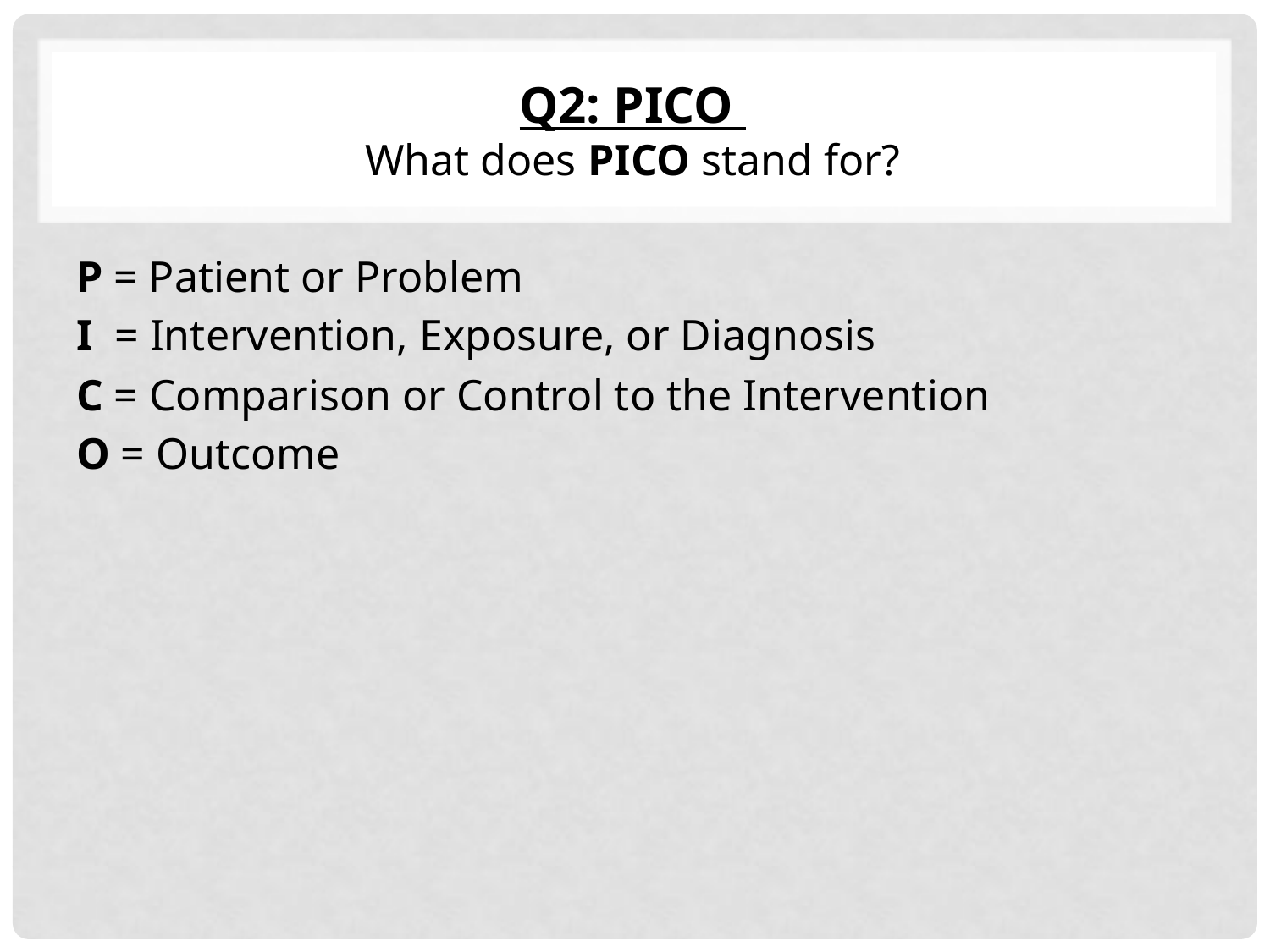

# Q2: PICO What does PICO stand for?
P = Patient or Problem
I = Intervention, Exposure, or Diagnosis
C = Comparison or Control to the Intervention
O = Outcome

## Slide 4
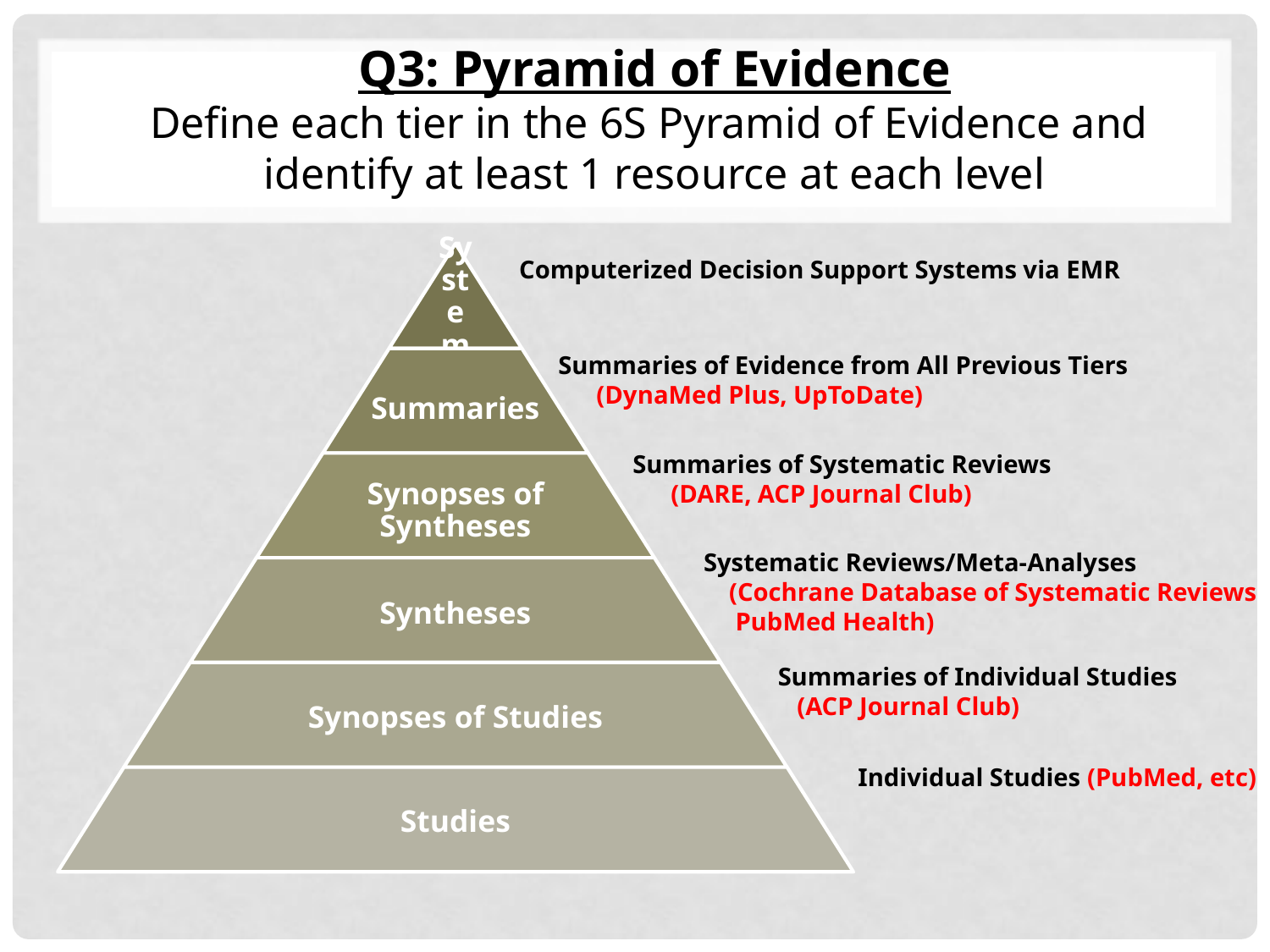

# Q3: Pyramid of Evidence Define each tier in the 6S Pyramid of Evidence and identify at least 1 resource at each level
Computerized Decision Support Systems via EMR
Summaries of Evidence from All Previous Tiers
 (DynaMed Plus, UpToDate)
Summaries of Systematic Reviews
 (DARE, ACP Journal Club)
Systematic Reviews/Meta-Analyses
 (Cochrane Database of Systematic Reviews
 PubMed Health)
Summaries of Individual Studies
 (ACP Journal Club)
Individual Studies (PubMed, etc)
