# Supplementary material for: An Integrated, Case-Based Approach to Teaching Medical Students How to Locate the Best Available Evidence for Clinical Care
Source: MedEdPORTAL. 2017 Jan 19;13:10531. doi: 10.15766/mep_2374-8265.10531 (PMC6342155; doi:10.15766/mep_2374-8265.10531)
Supplement: Supplementary file 1 — A. Locating the Best Available Evidence Lecture-Text.docx B. Locating the Best Available Evidence Lecture.pptx C. Lab Facilitator Guide.docx D. Lab Review Questions.pptx E. Lab Worksheet Case 1-Blank.docx F. Lab Worksheet Case 1-Answer Key.docx G. Lab Worksheet Case 2-Blank.docx H. Lab Worksheet Case 2-Answer Key.docx I. Case Presentation Evaluation Rubric.docx [file mep-13-10531-s001.zip › E. Lab Worksheet Case 1-Blank.docx]

Case Project Team Members:_____________________

_____________________

_____________________

_____________________

_____________________

**Locating the Best Available Evidence Lab**

In this lab, you will simulate conducting the first two steps of the evidence-based medicine process by 1) formulating a clear clinical question and 2) gathering the evidence from various evidence-based resources for one patient case and your team case presentation project. This lab will prepare you not only for the upcoming sessions of this course, but also for your clerkships, residency, and careers where you will be expected to find, evaluate, and present evidence for patient cases and journal clubs.


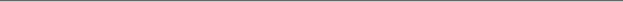


**CASE 1:** A 47-year-old male patient comes in for his annual physical. Overall, he is healthy and active with no prior history of heart disease. However, his father just recently suffered a stroke and he questions whether he is doing everything he can to protect his heart. His father now takes aspirin on a daily basis for secondary prevention of another attack and he wonders if he should as well.

| **STEP 1: ASK**  **PICO Analysis** Take 1 - 2 minutes to complete your PICO analysis individually and then as a team discuss and agree on your final PICO components and develop your clinical question. | |
| --- | --- |
| **INDIVIDUAL** | **TEAM** |
| **P**atient/Problem:  **I**ntervention:  **C**omparison, if applicable:  **O**utcome: | **P**atient/Problem:  **I**ntervention:  **C**omparison, if applicable:  **O**utcome: |
| **What is your clinical question based on your PICO analysis?** | |
| **What type of clinical question is this (highlight your answer in yellow)?**  🞏 Therapy/Prevention 🞏 Diagnosis 🞏 Etiology/Harm 🞏 Prognosis | |

**STEP 2: ACQUIRE - GATHER THE EVIDENCE** Individually, take 15 – 20 minutes to search each of the following resources for the evidence to answer the clinical question you developed above. Once you have completed your searches individually, discuss your findings as a team and provide the best answer below.

For this case, start searching at the bottom of the 6S Pyramid and work your way up. Copy and paste the evidence you find in each resource into the following table and jot down any observations concerning the strengths and weakness of the resource, including ***ease of use, quality of evidence you find, your confidence in locating the best evidence using this resource, and number of results you get***.

| **Resource** | **Evidence You Found** | **Your Observations** | | |
| --- | --- | --- | --- | --- |
|  |  | **Strengths** | **Weaknesses** | |
| **EXAMPLE: DynaMed Plus** | According to the National Asthma Education & Prevention Program: “inhaled steroids in ED reduces hospital admission rates in patients with acute asthma (level 1 [likely reliable] evidence), but additional benefit uncertain in patients receiving systemic corticosteroids”  “insufficient evidence for firm conclusions regarding use of inhaled corticosteroids in addition to or instead of oral corticosteroids after ED discharge for acute asthma”  *(From Topic Summary: Inhaled corticosteroids for acute asthma in children)* | - Easy to search -- single search box - All topics are critically appraised & evidence-based, so confidence in evidence found is high - Small set of search results can be read quickly & efficiently | - Not as comprehensive (only 3200 topics) as other Summary resources, such as UpToDate - Expert physician recommendations not provided | |
| PubMed  Clinical Queries |  |  |  | |
| Cochrane Database of Systematic Reviews |  |  |  | |
| DARE (via PubMed Health) |  |  |  | |
| DynaMed Plus |  |  |  | |
| UpToDate |  |  |  | |
| **Based on the evidence you found, what is the answer to your clinical question?** Describe how your team reached this conclusion including: **1)** were there any discrepancies in the evidence you found across the resources? **AND 2)** in your opinion, what was the best resource for locating the evidence taking into consideration how the following criteria influenced your decision: ease of use, quality of evidence you found, your confidence in locating the best evidence using this resource, and the number of results you retrieved. | | | |  |

**TEAM CASE PRESENTATION:** The second half of this lab gives you time as a team to work on your EBM Case Presentation, which your team will present as an oral presentation at the end of the course. Using the case presented in yesterday’s introductory session, discuss what aspect of the case you would like to tackle and work through the first two steps of the EBM process, ASK and ACQUIRE, for your team’s case project. Your team may approach the case from any perspective (therapy, diagnosis, prognosis, etc).

| **STEP 1: ASK**  **PICO Analysis** As a team, complete your PICO analysis |
| --- |
| **P**atient/Problem:  **I**ntervention:  **C**omparison, if applicable:  **O**utcome: |
| **What is your clinical question based on your PICO analysis?** |
| **What type of clinical question is this (highlight your answer in yellow)?**  🞏 Therapy/Prevention 🞏 Diagnosis 🞏 Etiology/Harm 🞏 Prognosis |

**STEP 2: ACQUIRE - GATHER THE EVIDENCE** Assign one resource to each team member. Take 5 minutes to search your resource for the evidence to answer the clinical question your team developed above. Take 10 – 15 minutes to present the evidence you found to your team.

| **Resource** | **Evidence You Found** |
| --- | --- |
|  |  |
| UpToDate |  |
| DynaMed Plus |  |
| DARE (via CRD) |  |
| Cochrane Database of Systematic Reviews |  |
| PubMed Clinical Queries |  |

| **You will eventually need to appraise the evidence you found in order to reach a conclusion, but just based on the evidence you have collected, what are your initial findings and observations about your clinical question related to the case?** |
| --- |
