# Supplementary material for: An Integrated, Case-Based Approach to Teaching Medical Students How to Locate the Best Available Evidence for Clinical Care
Source: MedEdPORTAL. 2017 Jan 19;13:10531. doi: 10.15766/mep_2374-8265.10531 (PMC6342155; doi:10.15766/mep_2374-8265.10531)
Supplement: Supplementary file 1 — A. Locating the Best Available Evidence Lecture-Text.docx B. Locating the Best Available Evidence Lecture.pptx C. Lab Facilitator Guide.docx D. Lab Review Questions.pptx E. Lab Worksheet Case 1-Blank.docx F. Lab Worksheet Case 1-Answer Key.docx G. Lab Worksheet Case 2-Blank.docx H. Lab Worksheet Case 2-Answer Key.docx I. Case Presentation Evaluation Rubric.docx [file mep-13-10531-s001.zip › F. Lab Worksheet Case 1-Answer Key.docx]

Case Project Team Members:_____________________

_____________________

_____________________

_____________________

_____________________

**Locating the Best Available Evidence Lab KEY**

In this lab, you will simulate conducting the first two steps of the evidence-based medicine process by 1) formulating a clear clinical question and 2) gathering the evidence from various evidence-based resources for one patient case and your team case presentation project. This lab will prepare you not only for the upcoming sessions of this course, but also for your clerkships, residency, and careers where you will be expected to find, evaluate, and present evidence for patient cases and journal clubs.


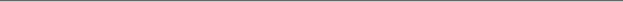


**CASE 1:** A 47-year-old male patient comes in for his annual physical. Overall, he is healthy and active with no prior history of heart disease. However, his father just recently suffered a stroke and he questions whether he is doing everything he can to protect his heart. His father now takes aspirin on a daily basis for secondary prevention of another attack and he wonders if he should as well.

| **STEP 1: ASK**  **PICO Analysis** Take 1 - 2 minutes to complete your PICO analysis individually and then as a team discuss and agree on your final PICO components | |
| --- | --- |
| **INDIVIDUAL** | **TEAM** |
| **P**atient/Problem:  Middle-aged male with no history of heart disease  **I**ntervention:  Aspirin for primary prevention of stroke  **C**omparison, if applicable:  None OR no aspirin  **O**utcome:  Effective prevention of stroke | **P**atient/Problem:  **I**ntervention:  **C**omparison, if applicable:  **O**utcome: |
| **What is your clinical question based on your PICO analysis?**  How effective is aspirin compared to no aspirin in the primary prevention of heart disease? | |
| **What type of clinical question is this (highlight your answer in yellow)?**  🞏 Therapy/Prevention 🞏 Diagnosis 🞏 Etiology/Harm 🞏 Prognosis | |

**STEP 2: ACQUIRE - GATHER THE EVIDENCE** Individually, take 15 – 20 minutes to search each of the following resources for the evidence to answer the clinical question you developed above. Once you have completed your searches individually, discuss your findings as a team and provide the best answer.

For this case, start searching at the bottom of the 6S Pyramid and work your way up. Copy and paste the evidence you find in each resource into the following table and jot down any observations concerning the strengths and weakness of the resource, including ***ease of use, quality of evidence you find, your confidence in locating the best evidence using this resource, and number of results you get***. Make sure to cite the individual articles or the topic summaries you get your evidence from as they should be findable by someone else.

| **Resource** | **Evidence You Found** | **Your Observations** | | |
| --- | --- | --- | --- | --- |
|  |  | **Strengths** | **Weaknesses** |  |
| PubMed  Clinical Queries | Various original studies and systematic reviews of the literature do NOT support use of aspirin for primary prevention of most cardiovascular events, but may reduce myocardial infarction.  **Sample Articles:**  Brotons C et al. A systematic review of aspirin in primary prevention: is it time for a new approach? Am J Cardiovasc Drugs. 2015;15(2):113-33.   - “Available data on aspirin in primary prevention suggest a modest benefit for patients at high risk of CVD, and a promising benefit for those at risk of cancer. Future studies should help to elucidate whether the benefit of aspirin outweighs risk in appropriate patient groups.”   Hissett J et al. Effects of changing guidelines on prescribing aspirin for primary prevention of cardiovascular events. J Am Board Fam Med. 2014; 27(1):78-86.   - “Even with new evidence against the use of aspirin for primary prevention, it is difficult to change beliefs about the effectiveness and safety of aspirin, as reflected in the behavior of physicians and patients.”   Sutcliffe P et al. Aspirin for prophylactic use in the primary prevention of cardiovascular disease and cancer: a systematic review and overview of reviews. Health Technol Assess. 2013; 17(43):1-253.   - “We have found that there is a fine balance between benefits and risks from regular aspirin use in primary prevention of CVD.” | - Easy to use - More specific to clinical questions than simply searching PubMed - Ability to narrow to specific clinical question type | - Difficult to come to any conclusion based on individual studies (low confidence in locating the best evidence) - Must judge quality of evidence and study methodology – time consuming - Still many results to parse through (762 results with a search of aspirin AND primary prevention AND stroke) |  |
| Cochrane Database of Systematic Reviews | Not much luck with Cochrane on finding relevant systematic reviews. Much more lunch with PubMed Clinical Queries > Systematic Reviews.  Most recent Cochrane Review seems to be from 2004. | - Quality of evidence is higher than individual studies - Fairly easy to use – just need to remember to go into Search Limits and check Cochrane Reviews | - Fewer results - Systematic review articles are long and time-consuming to read |  |
| DARE (via PubMed Health) | *UPDATE FOR 2015: A few new systematic reviews were published in 2014 that may have new information, but no commentaries were available in DARE – probably because DARE is no longer adding summaries.*  “This review concluded that the use of aspirin for the prevention of cardiac events needed to be weighed against an increase in major bleeds. There was no justification in advocating the routine use of aspirin.” (From Review: [Aspirin in the primary and secondary prevention of vascular disease: collaborative meta-analysis of individual participant data from randomized trials](http://www.crd.york.ac.uk/crdweb/ShowRecord.asp?AccessionNumber=12009105092&UserID=0) 2009)  “This review concluded that aspirin prevented deaths, myocardial infarction and ischaemic stroke and increased hemorrhagic stroke and major bleeding when used in the primary prevention of cardiovascular disease.” (From Review: [Effect of aspirin on mortality in the primary prevention of cardiovascular disease](http://www.crd.york.ac.uk/crdweb/ShowRecord.asp?AccessionNumber=12011004166&UserID=0) 2011) | - Easy to search - Expert commentary on systematic reviews is quick and easy to read - Fewer results is easier to parse through, but may be limiting - Freely available online via University of York CRD | - Not a synopsis of synthesis for every systematic review or meta-analysis published |  |
| DynaMed Plus | Use is strongly recommended by the United States Preventive Services Task Force and other organizations based on patient age, gender and risk, especially:   - “men aged 45-79 years if potential reduction in risk of myocardial infarction outweighs potential harm to due gastrointestinal hemorrhage ([USPSTF Grade A recommendation](http://web.a.ebscohost.com/dynamed/detail?vid=3&sid=9a8222a7-dd9f-4dd6-aa5a-faffc04435fb%40sessionmgr4002&hid=4212&bdata=JnNpdGU9ZHluYW1lZC1saXZlJnNjb3BlPXNpdGU%3d#uspstfgrade))” - “women aged 55-79 years if potential reduction in risk of myocardial infarction outweighs potential harm to due gastrointestinal hemorrhage ([USPSTF Grade A recommendation](http://web.a.ebscohost.com/dynamed/detail?vid=3&sid=9a8222a7-dd9f-4dd6-aa5a-faffc04435fb%40sessionmgr4002&hid=4212&bdata=JnNpdGU9ZHluYW1lZC1saXZlJnNjb3BlPXNpdGU%3d#uspstfgrade))” - **“For primary prevention of cardiovascular disease in patients with hypertension, aspirin reduces myocardial infarction (MI), increases bleeding, and does not reduce strokes or total cardiovascular events (**[level 1 [likely reliable] evidence](http://www.epnet.com/dynamed/levels.php)**)”**   Actual evidence of efficacy varies by disease type and existing conditions, such as hypertension and diabetes (eg: strong evidence for reducing MI, but not stroke or total cardiovascular event).  Aspirin also increases risk for GI bleeding. *(From Summary: Aspirin for primary prevention of cardiovascular disease – updated April 2015)* | - Very easy to search with single search box - All topics are critically appraised and evidence-based - Integration of 3-tier grading system allows for quick analysis and judgment of the evidence - Combines evidence across the four lower tiers of the pyramid | - Not as comprehensive as UpToDate with only 3200 topics versus 10,000 - Mobile app is difficult to download |  |
| UpToDate | BOTTOM LINE: “A decision regarding aspirin use for primary prevention (of both cancer and cardiovascular disease events) should be made with the patient, after presenting an understandable summary of the likely patient-specific benefits and risks.”  Findings from several meta-analyses suggest that aspirin produces:   - “A 20 percent relative risk reduction in non-fatal myocardial infarction (MI) (OR 0.80, 95% CI 0.67-0.96). - No significant impact on non-fatal stroke (including ischemic and hemorrhagic stroke) - No significant impact on CVD mortality. - A 54 percent increase in the relative risk of non-fatal extracranial bleeding (RR 1.54, 95% CI 01.30-1.82)   (From Summary: Aspirin in the primary prevention of cardiovascular disease and cancer – updated March 2015) | - Very easy to search with single search box - Fairly universal in all hospital environments - Use of GRADE system provides quick and easy analysis of recommendations - Combines evidence across the four lower tiers of the pyramid | - Not all information has been critically appraised |  |

| **Based on the evidence you found, what is the answer to your clinical question?** Describe how your team reached this conclusion including: **1)** were there any discrepancies in the evidence you found across the resources? **AND 2)** in your opinion, what was the best resource for locating the evidence taking into consideration how the following criteria influenced your decision: ease of use, quality of evidence you found, your confidence in locating the best evidence using this resource, and the number of results you retrieved.  Answer to Clinical Question: Depending on the patient’s age, family history, existing chronic diseases, and gender of patient, use of aspirin for primary prevention of cardiovascular disease and stroke may outweigh risks of GI bleeding. It really comes down to the clinician’s judgment based on the individual patient.  There is no correct answer when describing how their team reached their conclusion. It is just meant to help them think through some criteria for evaluating an EBM source based on the evidence, grading systems, ease of use, etc. |
| --- |
