# Supplementary material for: An Integrated, Case-Based Approach to Teaching Medical Students How to Locate the Best Available Evidence for Clinical Care
Source: MedEdPORTAL. 2017 Jan 19;13:10531. doi: 10.15766/mep_2374-8265.10531 (PMC6342155; doi:10.15766/mep_2374-8265.10531)
Supplement: Supplementary file 1 — A. Locating the Best Available Evidence Lecture-Text.docx B. Locating the Best Available Evidence Lecture.pptx C. Lab Facilitator Guide.docx D. Lab Review Questions.pptx E. Lab Worksheet Case 1-Blank.docx F. Lab Worksheet Case 1-Answer Key.docx G. Lab Worksheet Case 2-Blank.docx H. Lab Worksheet Case 2-Answer Key.docx I. Case Presentation Evaluation Rubric.docx [file mep-13-10531-s001.zip › G. Lab Worksheet Case 2-Blank.docx]

**Locating the Best Available Evidence Lab - Sample Case 2**

In this lab, you will simulate conducting the first two steps of the evidence-based medicine process by 1) formulating a clear clinical question and 2) gathering the evidence from various evidence-based resources for one patient case and your team case presentation project. This lab will prepare you not only for the upcoming sessions of this course, but also for your clerkships, residency, and careers where you will be expected to find, evaluate, and present evidence for patient cases and journal clubs.


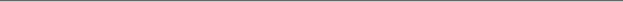


**CASE 2:** A 13-month-old infant has a suspected allergy to peanuts after two incidents of rash, diarrhea, and vomiting only hours after eating peanut butter. Her mother didn’t introduce peanuts into her diet until she was one-year-old and can think of no other diet or environmental changes since these incidents started and she wants a skin test performed to confirm. You are not sure if skin tests are an appropriate diagnostic method for food allergies and wonder if a blood test would be more effective.

| **STEP 1: ASK**  **PICO Analysis** Complete your PICO analysis |
| --- |
| **P**atient/Problem:  **I**ntervention:  **C**omparison, if applicable:  **O**utcome: |
| **What is your clinical question based on your PICO analysis?** |
| **What type of clinical question is this (highlight your answer in yellow)?**  🞏 Therapy/Prevention 🞏 Diagnosis 🞏 Etiology/Harm 🞏 Prognosis |

**STEP 2: ACQUIRE - GATHER THE EVIDENCE** Search each of the following resources for the evidence to answer the clinical question you developed above and document the evidence you found in the table.

| **Resource** | **Evidence You Found** |
| --- | --- |
|  |  |
| UpToDate |  |
| DynaMed Plus |  |
| DARE (via PubMed Health) |  |
| Cochrane Database of Systematic Reviews |  |
| PubMed Clinical Queries |  |

| **Based on the evidence your team found in the various resources, what is the answer to your clinical question?** **If your diagnostic tests are inconclusive,** **what other recommendations do you have for the infant’s mother based on your findings?** |
| --- |
