# Supplementary material for: An Integrated, Case-Based Approach to Teaching Medical Students How to Locate the Best Available Evidence for Clinical Care
Source: MedEdPORTAL. 2017 Jan 19;13:10531. doi: 10.15766/mep_2374-8265.10531 (PMC6342155; doi:10.15766/mep_2374-8265.10531)
Supplement: Supplementary file 1 — A. Locating the Best Available Evidence Lecture-Text.docx B. Locating the Best Available Evidence Lecture.pptx C. Lab Facilitator Guide.docx D. Lab Review Questions.pptx E. Lab Worksheet Case 1-Blank.docx F. Lab Worksheet Case 1-Answer Key.docx G. Lab Worksheet Case 2-Blank.docx H. Lab Worksheet Case 2-Answer Key.docx I. Case Presentation Evaluation Rubric.docx [file mep-13-10531-s001.zip › H. Lab Worksheet Case 2-Answer Key.docx]

**Locating the Best Available Evidence Lab - Sample Case 2 KEY**

In this lab, you will simulate conducting the first two steps of the evidence-based medicine process by 1) formulating a clear clinical question and 2) gathering the evidence from various evidence-based resources for one patient case and your team case presentation project. This lab will prepare you not only for the upcoming sessions of this course, but also for your clerkships, residency, and careers where you will be expected to find, evaluate, and present evidence for patient cases and journal clubs.


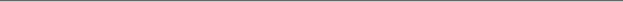


**CASE 2:** A 13-month-old infant has a suspected allergy to peanuts after two incidents of rash, diarrhea, and vomiting only hours after eating peanut butter. Her mother didn’t introduce peanuts into her diet until she was one-year-old and can think of no other diet or environmental changes since these incidents started and she wants a skin test performed to confirm. You are not sure if skin tests are an appropriate diagnostic method for food allergies and wonder if a blood test would be more effective.

| **STEP 1: ASK**  **PICO Analysis** Complete your PICO analysis |
| --- |
| **P**atient/Problem:  Infant with suspected peanut allergy  **I**ntervention:  Skin test  **C**omparison, if applicable:  Blood test  **O**utcome:  Successful diagnosis of allergy |
| **What is your clinical question based on your PICO analysis?**  Is a blood test or skin test more effective in diagnosing an infant with a suspected peanut allergy? |
| **What type of clinical question is this (highlight your answer in yellow)?**  🞏 Therapy/Prevention 🞏 Diagnosis 🞏 Etiology 🞏 Prognosis |

**STEP 2: ACQUIRE - GATHER THE EVIDENCE** Search each of the following resources for the evidence to answer the clinical question you developed above and document the evidence you found in the table.

| **Resource** | **Evidence You Found** |
| --- | --- |
|  |  |
| UpToDate | “A positive test for specific IgE antibodies (skin prick test or in vitro test) is usually sufficient to establish the diagnosis for a suspected IgE-mediated reaction in the setting of an unequivocal history.”  “Double-blind, placebo-controlled oral food challenges (DBPCFC) are the gold standard for the diagnosis of food allergy. A clinician-supervised oral food challenge is required if the history and IgE test results do not clearly indicate an allergy.”  *(From Summary: Peanut, tree nut, and seed allergy: Diagnosis)* |
| DynaMed Plus | “immediate-reacting allergy skin tests and in vitro immunoglobulin E antibody tests can be used to screen for food allergy   - skin testing   - **immediate reaction suggests presence of food-specific** **IgE antibodies and indicates likelihood of allergy**   - negative test rules out allergy - in vitro testing of food-specific IgE antibodies   - less sensitive than skin testing   - safer for child with history of life-threatening systemic allergic-like reactions - sufficient for presumptive diagnosis of food allergy if positive skin test or IgE food-specific antibodies and history consistent with allergic reaction to food commonly associated with anaphylaxis”   **“only food challenge can confirm reaction to particular food”** *(From Topic Summary: Food allergy)* |
| DARE (via PubMed Health) | Notes on Search:   - peanut allergy AND diagnosis yields 0 results; food allergy AND diagnosis yields 4 - Most recent systematic review from 2013 “The diagnosis of food allergy: a systematic review and meta-analysis” has no commentary yet   “There were no statistically significant differences between skin prick tests compared with food challenge (AUC 0.87, 95% CI 0.81 to 0.93; 13 studies) or serum food-specific IgE tests compared with food challenge (AUC 0.84, 95% CI 0.78 to 0.91; 11 studies) for all food allergies.” *(From “*[*Diagnosing and managing common food allergies: a systematic review*](http://www.crd.york.ac.uk/CRDWeb/ShowRecord.asp?AccessionNumber=12010003094&UserID=0#.UyMdpF6nSAc)*”).* Expert commentary suggests elements of bias present in the systematic review including publication bias, language filters bias, difficult to ascertain validity assessment of studies included in the review. |
| Cochrane Database of Systematic Reviews | Notes on Search: peanut allergy AND diagnosis, food allergy AND diagnosis yield no results; general search for food allergy yields 13 – none are related to diagnosis, only therapy and prevention |
| PubMed Clinical Queries | Peanut allergy AND skin test AND blood test yields 57 results when limited to Diagnosis and Broad – several studies look at efficacy of using skin versus IgE tests  Note: PubMed also does not list any systematic reviews via Clinical Queries |

| **Based on the evidence your team found in the various resources, what is the answer to your clinical question?** **If your diagnostic tests are inconclusive,** **what other recommendations do you have for the infant’s mother based on your findings?**  Both a skin prick test and in vitro blood test should be conducted to test for immediate reaction to food specific IgE antibodies. The best recommendation to the infant’s mother if these two tests are inconclusive is a referral to a allergy specialist who would perform an oral food challenge, which is the gold standard for diagnosis of a food allergy. |
| --- |
