# Supplementary material for: An Integrated, Case-Based Approach to Teaching Medical Students How to Locate the Best Available Evidence for Clinical Care
Source: MedEdPORTAL. 2017 Jan 19;13:10531. doi: 10.15766/mep_2374-8265.10531 (PMC6342155; doi:10.15766/mep_2374-8265.10531)
Supplement: Supplementary file 1 — A. Locating the Best Available Evidence Lecture-Text.docx B. Locating the Best Available Evidence Lecture.pptx C. Lab Facilitator Guide.docx D. Lab Review Questions.pptx E. Lab Worksheet Case 1-Blank.docx F. Lab Worksheet Case 1-Answer Key.docx G. Lab Worksheet Case 2-Blank.docx H. Lab Worksheet Case 2-Answer Key.docx I. Case Presentation Evaluation Rubric.docx [file mep-13-10531-s001.zip › I. Case Presentation Evaluation Rubric.docx]

**Team Case Presentation Rubric**

The oral presentation provides an opportunity for you to teach, share, and demonstrate how and what you have learned throughout this course. You have 5-8 minutes for your presentation.

Each team will be evaluated in the areas presented below. All team members will receive the same grade for the group presentation, which will be an average of multiple independent faculty ratings.

| **Components of Group Oral Presentation** | **Fails to Meet Expectations**  **(0 points)** | **Below Expectations**  **(7 points)** | **Meets Expectations**  **(8 points)** | **Exceeds Expectations**  **(10 points)** |
| --- | --- | --- | --- | --- |
|  |  |  |  |  |
| Case: Presentation of the salient details of the clinical case (relative to the PICO question). |  |  |  |  |
|  |  |  |  |  |
| PICO: A clear well-built clinical question derived from the clinical case and based on the components of PICO. |  |  |  |  |
|  |  |  |  |  |
| Search: Description of the searches performed, including PubMed strategies and other EBM resources (i.e. Cochrane Library, DynaMed, UpToDate, etc.). References presented in AMA style at the end of the presentation. |  |  |  |  |
|  |  |  |  |  |
| Summary: Summary of the evidence that answers the clinical question. 3-5 bullet points of key evidence (noting the strength of each piece of the evidence with SORT or other grading system, if applicable). |  |  |  |  |
|  |  |  |  |  |
| Conclusion: Provides a clinical solution/bottom line for patient care based on the clinical case (relative to the PICO question) |  |  |  |  |
|  |  |  |  |  |
| Quality of Presentation: Including adequate preparation, pace of delivery, eye contact, clarity of speech, within required presentation length, professionalism of PowerPoint slides (i.e. no typos, grammatically correct, consistency of appearance, etc.) |  |  |  |  |
|  |  |  |  |  |
